# Integrated Genotoxicity Testing of three anti-infective drugs using the TGx-DDI transcriptomic biomarker and high-throughput CometChip^®^ assay in TK6 cells

**DOI:** 10.3389/ftox.2022.991590

**Published:** 2022-09-23

**Authors:** Julie K. Buick, Andrea Rowan-Carroll, Rémi Gagné, Andrew Williams, Renxiang Chen, Heng-Hong Li, Albert J. Fornace, Christy Chao, Bevin P. Engelward, Roland Frötschl, Heidrun Ellinger-Ziegelbauer, Syril D. Pettit, Jiri Aubrecht, Carole L. Yauk

**Affiliations:** ^1^ Environmental Health Science and Research Bureau, Health Canada, Ottawa, ON, Canada; ^2^ Department of Oncology, Lombardi Comprehensive Cancer Center, Georgetown University Medical Center, Washington, DC, United States; ^3^ Department of Biochemistry and Molecular and Cellular Biology, Georgetown University, Washington, DC, United States; ^4^ Department of Chemical Engineering, Massachusetts Institute of Technology, Cambridge, MA, United States; ^5^ Department of Biological Engineering, Massachusetts Institute of Technology, Cambridge, MA, United States; ^6^ Federal Institute for Drugs and Medical Devices (BfArM), Bonn, Germany; ^7^ Bayer AG, Pharmaceuticals, Preclinical Development, Wuppertal, Germany; ^8^ Health and Environmental Sciences Institute, Washington, DC, United States; ^9^ Department of Biology, University of Ottawa, Ottawa, ON, Canada

**Keywords:** genetic toxicology, TGx-28.65 genomic biomarker, toxicogenomics, nitrofurantoin, metronidazole, novobiocin

## Abstract

Genotoxicity testing relies on the detection of gene mutations and chromosome damage and has been used in the genetic safety assessment of drugs and chemicals for decades. However, the results of standard genotoxicity tests are often difficult to interpret due to lack of mode of action information. The TGx-DDI transcriptomic biomarker provides mechanistic information on the DNA damage-inducing (DDI) capability of chemicals to aid in the interpretation of positive *in vitro* genotoxicity data. The CometChip^®^ assay was developed to assess DNA strand breaks in a higher-throughput format. We paired the TGx-DDI biomarker with the CometChip^®^ assay in TK6 cells to evaluate three model agents: nitrofurantoin (NIT), metronidazole (MTZ), and novobiocin (NOV). TGx-DDI was analyzed by two independent labs and technologies (nCounter^®^ and TempO-Seq^®^). Although these anti-infective drugs are, or have been, used in human and/or veterinary medicine, the standard genotoxicity testing battery showed significant genetic safety findings. Specifically, NIT is a mutagen and causes chromosome damage, and MTZ and NOV cause chromosome damage in conventional *in vitro* tests. Herein, the TGx-DDI biomarker classified NIT and MTZ as non-DDI at all concentrations tested, suggesting that NIT’s mutagenic activity is bacterial specific and that the observed chromosome damage by MTZ might be a consequence of *in vitro* test conditions. In contrast, NOV was classified as DDI at the second highest concentration tested, which is in line with the fact that NOV is a bacterial DNA-gyrase inhibitor that also affects topoisomerase II at high concentrations. The lack of DNA damage for NIT and MTZ was confirmed by the CometChip^®^ results, which were negative for all three drugs except at overtly cytotoxic concentrations. This case study demonstrates the utility of combining the TGx-DDI biomarker and CometChip^®^ to resolve conflicting genotoxicity data and provides further validation to support the reproducibility of the biomarker.

## Introduction

Conventional toxicological test methods are not sufficient to fully address current risk assessment requirements as they are resource-intensive, low-throughput, and often lack mechanistic context (National Research Council, 2007). The vision for toxicity testing in the 21st century is to replace older, inadequate toxicity tests with modern *in silico* and *in vitro* testing alternatives that use human-relevant models in high-throughput (HT) designs, aligned with risk assessment needs (Adeleye et al., 2015; Choudhuri et al., 2018; Krewski et al., 2020). New approach methodologies (NAMs) are being developed to capitalize on advances in both biological sciences and computational approaches to address these needs in all subdisciplines of toxicology.

Genotoxicity assessment is critical to evaluating the toxic potential of drugs and chemicals, as genetic changes such as mutations, chromosome damage, and subsequent genomic instability, can lead to cancer and inherited genetic disease (Phillips and Arlt, 2009). The standard test battery typically includes the Ames bacterial reverse mutation assay, an *in vitro* mammalian genotoxicity test (e.g., chromosome aberrations (CA), micronuclei (MN) and/or gene mutations) and/or an *in vivo* rodent genotoxicity assay (e.g., CA, MN and/or transgene mutations) (Lynch et al., 2011; Dearfield et al., 2017; Galloway, 2017; Turkez et al., 2017). These tests measure a single endpoint, are prone to producing irrelevant results with respect to human cancer risk, and provide limited mode of action (MoA) information (Nesslany, 2017; Turkez et al., 2017). Therefore, the development of new mechanism-based *in vitro* genotoxicity tests in human cells is essential for better elucidation of human risk following exposure to genotoxic agents.

Transcriptomics, which analyzes genome wide gene expression changes, has been extensively explored for its potential to revolutionize toxicity testing. High-throughput transcriptomic biomarkers that predict specific adverse outcomes have the potential to provide a streamlined approach to quickly identify key events in MoAs and potential hazards (Krewski et al., 2020). We developed a 64-gene transcriptomic biomarker (called TGx-DDI) from the global gene expression profiles of a training set of 28 well-characterized DNA damage-inducing (DDI) and non-DDI reference chemicals in human TK6 cells (Buick et al., 2015; Li et al., 2015; Li et al., 2017). The TGx-DDI biomarker classifies agents as DDI or non-DDI based on the specific expression profiles of these biomarker genes. The biomarker has been validated in the presence and absence of metabolic activation (i.e., rat liver S9), in two different human cell lines (i.e., TK6 cells and HepaRG™ cells), and using a number of different gene expression technologies, including DNA microarray, quantitative PCR arrays, HT digital detection (i.e., nCounter^®^) and RNA sequencing (i.e., AmpliSeq and TempO-Seq^®^) (Buick et al., 2015; Li et al., 2015; Li et al., 2017; Corton et al., 2018; Cho et al., 2019; Li et al., 2019; Buick et al., 2020; Buick et al., 2021). In addition, a HT screening approach has recently been reported using a direct-lysate nCounter^®^ approach (Chen et al., 2022). Two workflows have been proposed to apply the TGx-DDI genomic biomarker for genotoxicity assessment for pharmaceutical drug development and for industrial and environmental chemicals (Li et al., 2017). For industrial and environmental chemicals, the TGx-DDI biomarker can be used in a HT strategy to identify, characterize, and prioritize chemicals that may result in DNA damage, whereas, for drug candidates, the biomarker has been proposed as part of the “weight of evidence” in assessing the relevance of results of the genotoxicity testing battery.

The main objective of the present study was to apply an integrated testing strategy consisting of the TGx-DDI biomarker and the HT CometChip^®^ assay in human TK6 cells for evaluating genotoxic hazards of three well-known pharmaceuticals with complex findings in standard genotoxicity tests. Specifically, the TGx-DDI biomarker was used to assess the potential of case study pharmaceuticals to induce a DNA damage response and the CometChip^®^ assay was used as a follow-up test to assess DNA strand breaks. A secondary objective was to further validate the TGx-DDI biomarker by confirming the reproducibility of TGx-DDI classifications across laboratories and technologies (i.e., NanoString nCounter^®^ and BioSpyder TempO-Seq^®^).

Three anti-infective agents, nitrofurantoin (NIT), metronidazole (MTZ), and novobiocin (NOV), were selected as case study chemicals. While these anti-infective drugs are currently, or have previously been, used in human and/or veterinary medicines, significant genetic safety findings have been demonstrated using standard genotoxicity tests. Notably, NIT is a mutagen and causes chromosome damage, while MTZ and NOV cause chromosome damage in conventional *in vitro* tests. See Materials and Methods for a detailed description of the compounds. This case study provides proof of principle for application of the TGx-DDI biomarker for providing insight into conflicting genotoxic results for drug candidates, as well as in compound screening and prioritization for drug development. Moreover, this case study provides additional validation for the TGx-DDI biomarker using different gene expression technologies across different laboratories.

## Materials and methods

### Case study chemical selection and rationale

Case Study Drug #1: NIT is a nitrofuran derivative antibiotic that is used to treat urinary tract infections (UTIs) and has been used for over 60 years (Muller et al., 2017). Common brand names include Furadantin, Macrobid and Macrodantin (Prescribers’ Digital Reference Network, 2021). NIT has been shown to be mutagenic in the Ames assay due to activation by bacterial nitroreductases, which is not relevant for mammalian cells (Olive and McCalla, 1977; Ni et al., 1987; Mokdad-Bzeouich et al., 2015). NIT has also been shown to be weakly mutagenic in mammalian cells (Gao et al., 1989; Slapsyte et al., 2002), and in the kidneys of Big Blue transgenic mice (Quillardet et al., 2006). Additionally, NIT can induce chromosome damage (Parodi et al., 1983; Thompson, 1986). Despite experimental evidence of genotoxicity, the long-term antimicrobial treatment with NIT is generally considered safe (Uhari et al., 1996); thus, NIT provides an excellent model compound to evaluate the human relevance of the conflicting genotoxicity findings using the TGx-DDI biomarker.

Case Study Drug #2: MTZ is an antibacterial and antiprotozoal agent used to treat anaerobic bacterial infections and protozoal infections in human and veterinary applications. It is also prescribed off-label in the treatment of rosacea and Crohn’s disease, as a post-surgical prophylactic agent, and in the treatment of *Helicobacter pylori* infection (National Center for Biotechnology Information, 2022). Common brand names include Flagyl, MetroCream, and Vandazole (Prescribers’ Digital Reference Network, 2021). MTZ has demonstrated mutagenic activity in the Ames bacterial reverse mutation assay, both induced by the drug itself (Melo and Ferreira, 1990) and by the urine of treated patients (Speck et al., 1976; Connor et al., 1977). In mammalian cells and in laboratory animals, conflicting evidence exists regarding the genotoxicity of MTZ (Buschini et al., 2009). Human studies have failed to demonstrate the potential for genetic damage. Due to the conflicting experimental data for the mutagenicity and genotoxicity of MTZ, this agent was selected as a case study chemical to provide further clarity on its genetic safety.

Case Study Drug #3: NOV, also known as albamycin, streptonivcin, or cathomycin, is a narrow-spectrum antibiotic drug. It is used in veterinary applications for the treatment of bovine mastitis in lactating and dry-off cows. NOV was also widely used as a human therapeutic (Committee for Veterinary Medicinal Products, 1999). NOV interacts directly with the B subunit (GyrB) of the bacterial DNA gyrase enzyme (Maxwell, 1993; Heide, 2009a) and it is a non-specific inhibitor of DNA topoisomerase II (DNA topoII) enzyme (Savoldi-Barbosa et al., 1999). NOV has been shown to cause concentration-dependent DNA fragmentation that preceded apoptosis in primary cultures of mouse thymocytes, by inhibiting DNA-rejoining activity of the enzyme (Onishi et al., 1993). While NOV administration initially demonstrated little toxicity (Kirby et al., 1956), it has since been withdrawn from the market for human therapeutic use due to poor pharmacological properties and liver toxicity (Shirude and Hameed, 2012). As a result, NOV was selected as a case study chemical to investigate its potential genotoxic hazard to clarify its genetic safety.

### Chemicals

Nitrofurantoin (NIT; CAS no. 67-20-9), metronidazole (MTZ; CAS no. 443-48-1), and novobiocin sodium salt (NOV; CAS no. 1476-53-5) were purchased from Sigma-Aldrich, Inc. (St. Louis, Missouri, United States). NIT (lot no. MKCC9622), MTZ (lot no. MKBZ3056V), and NOV (lot no. 2998909) were 100%, >99%, and 96.0% pure, respectively. Identical lot numbers were used across all laboratories. Dimethyl sulfoxide (DMSO; 0.125% for 2-250 μM, 0.25% for 500 μM, and 0.5% for 1,000 µM), acetic acid (HOAc; 0.0625% for 2-250 μM, 0.25% for 500 μM, and 0.5% for 1,000 µM) and water (H_2_O; 0.125% for 2-250 μM, 0.25% for 500 μM, and 0.5% for 1,000 µM) were used as vehicle controls for NIT, MTZ, and NOV, respectively. Caffeine (CA; 2 mM) was used as a negative control. Bleomycin (BL; 10 µM) and ionizing radiation (IR; 4 Gy) were used as positive controls for the NanoString nCounter^®^ and BioSpyder TempO-Seq^®^ experiments. Hydrogen peroxide (H_2_O_2_; 25-100 µM) was used as a positive control for the CometChip^®^ experiments.

### TK6 cell culture and chemical exposure

Human TK6 cells are the recommended cell line for TGx-DDI biomarker analysis. Extensive validation of TGx-DDI performance has been completed using TK6 cells, which were selected because they are widely used in genotoxicity testing and have an intact p53 response pathway (Li et al., 2019). In this study, TK6 cells (ATCC, Manassas, Virginia, United States) were exposed to ten concentrations of each test chemical in triplicate, as follows: 2.0, 3.9, 7.8, 15.6, 31.3, 62.5, 125, 250, 500, and 1,000 μM, in addition to matched vehicle controls. The maximum concentrations were determined based on guidance for top concentration selection for mammalian cell assays in the ICH Guideline S2 (R1) on Genotoxicity Testing and Data Interpretation for Pharmaceuticals Intended for Human Use (ICH, 2012). Cells were exposed to the extended concentration range of each chemical for 4 h in parallel 96-well plates for cytotoxicity analysis and 6-well plates for the collection of cells for gene expression analysis using nCounter^®^ and TempO-Seq^®^ for TGx-DDI classification at Georgetown University (GU). For the evaluation of DNA damage by CometChip^®^, a sterile 12-well reagent reservoir was used for cellular exposures at the Massachusetts Institute of Technology (MIT). One 12-well reservoir was used for each test chemical, and each well in this reservoir contained cells exposed to one of the ten chemical concentrations or matched vehicle controls. Then, treated cells were loaded into duplicate wells of the CometChip^®^ for DNA damage assessment. TK6 cells, a spontaneously transformed human lymphoblastoid cell line, were cultured in suspension in RPMI 1640 supplemented with 10% fetal bovine serum and treated with case study chemicals, as described previously (Li et al., 2015; Li et al., 2017). Specifically, exponentially growing cells were treated at a density of 4–5 × 10^5^ cells per mL in parallel 96-well plates. Following the 4 h exposure, cells were washed and collected for RNA extraction for subsequent gene expression analysis using NanoString nCounter^®^ and BioSpyder TempO-Seq^®^ gene expression analysis.

### MTT cell viability assay

The MTT assay (Cayman Chemical, Ann Arbor, MI, United States) was used to quantify cellular metabolic activity as an indicator of cell viability and was conducted at GU. TK6 cells were exposed for 4 h, as described in the previous section, then cells were washed and re-incubated in fresh media for a 20 h recovery period for cytotoxicity assessment. The cytotoxicity of the vehicle was tested and the concentration of vehicle that caused minimal (less than 10%) cytotoxicity was used. In the cases that a more concentrated vehicle was needed due to the solubility of the chemical, the corresponding vehicle controls were included as the reference. The MTT assay was performed in triplicate at the 24 h time point (4 h exposure, plus 20 h recovery) for all 10 concentrations plus the solvent controls, following the manufacturer’s instructions. Absorbance was measured at 570 nm using a microplate reader. The cytotoxicity threshold was 50%, in line with the guidance provided in OECD Test Guideline 487 for *In Vitro* Mammalian Cell MN Test and the ICH Guideline S2 (R1) on Genotoxicity Testing and Data Interpretation for Pharmaceuticals Intended for Human Use (ICH, 2012; OECD, 2016). Chemical conditions that surpassed the cytotoxicity threshold were excluded from the nCounter^®^ and TempO-Seq^®^ analyses.

### Total RNA extraction and purification

RNA extraction was performed using the protocol accompanying the TRIzol™ reagent (Invitrogen, part of ThermoFisher Scientific, Waltham, MA United States), which was then purified via an RNA cleanup step using an RNeasy column according to the manufacturer’s instructions (Qiagen, Germantown, MD United States). Purified RNA was quantified and analyzed for quality using a NanoDrop™ spectrophotometer and an Agilent BioAnalyzer, respectively.

### TGx-DDI nCounter^®^ assay

The TGx-DDI nCounter^®^ assay was conducted at GU using 100 ng of high quality, purified RNA. All non-cytotoxic concentrations of each test chemical were included in this analysis, in addition to the corresponding vehicle controls for each treatment. The highest two concentrations of MTZ and NIT were excluded (i.e., 500 μM and 1,000 µM), in addition to the highest concentration of NOV (i.e., 1,000 µM) due to levels of cytotoxicity exceeding the threshold of 50% cell death. The nCounter^®^ assay was performed in triplicate for each treatment and matched control. Caffeine (CA; 2 mM) served as a negative control, while bleomycin (BL; 10 µM) and ionizing radiation (IR; 4 Gy) were used as positive controls. nCounter^®^ experiments were conducted as previously reported (Li et al., 2017) and full details of the nCounter^®^ methods have been previously reported (Geiss et al., 2008). The nCounter^®^ experiments were conducted following the instructions outlined in the NanoString nCounter^®^ XT Assay User Manual (MAN-10023-11 July 2016). Briefly, optimized sequences for the TGx-DDI biomarker genes, plus eight housekeeping genes, including *G6PD*, *GUSB*, *HPRT1*, *LDHA*, *NONO*, *PGK1*, *PPIH*, and *TFRC*, were chosen based on stability and detectable expression levels and were included in a custom-designed CodeSet manufactured by NanoString. Unique barcodes were counted for each target, and the data were exported for analysis. The counts for each target were analyzed for quality control and normalization using nSolver™ Analysis version 4.0. Normalized data were exported and subjected to further analysis, which is described below in the Statistical and Bioinformatic Analyses for TGx-DDI Classification section.

### TempO-Seq^®^ library preparation and S1500+ targeted transcriptome sequencing

TempO-Seq^®^ gene expression analysis was conducted on all non-cytotoxic concentrations of exposed and control TK6 cell lysates at Health Canada. The 500 μM and 1,000 μM concentrations of MTZ and NIT were omitted, in addition to the 1,000 μM concentration of NOV, as these concentrations surpassed the cytotoxicity threshold of 50% cell death. The 2 μM concentrations were also eliminated in order to run the positive and negative TempO-Seq^®^ assay controls. Libraries were prepared in a 96-well plate format using the TempO-Seq^®^ Human Tox + Surrogate Panel reagent kit (BioSpyder Technologies, Carlsbad, CA, United States), following the company’s protocol. Human Brain Total RNA and qPCR Human Reference Total RNA (Takara Bio, CA, United States) were included as positive assay controls and a no-lysate control (1X TempO-Seq^®^ Lysis Buffer only) was included as a negative assay control (two replicates per control). The same positive and negative controls were included for TempO-Seq^®^ analysis as for nCounter^®^ analysis (i.e., 2 mM CA as a negative control; 10 μM BL and 4 Gy IR as positive controls). In brief, 100 ng of total RNA (in a 2 μl volume) from exposed and control cells were hybridized with the targeted Human S1500+ Tox Panel detector oligo (DO) probe mix (v1.1; 2,977 probes) in 1X TempO-Seq^®^ Lysis Buffer for 10 min at 70°C followed by a temperature gradient with a ramp rate of 0.5^°^C/min to 45°C over a 50 min period, followed by a nuclease digestion at 37°C for 90 min to remove excess, unbound, or incorrectly bound DOs enzymatically. Ligation of the DO pairs bound to adjacent target sequences was then completed with a 60 min incubation at 37°C, immediately followed with a 15 min enzyme denaturation at 80°C to generate a pool of amplification templates. All amplification templates (i.e., 10 μl of ligated DOs) were transferred to its corresponding well of the 96-well PCR Pre-Mix and Primers plate. Amplification proceeded with a CFX96 Real-Time PCR Detection System (Bio-Rad, Mississauga, ON, Canada) to attach a unique sequence tag and the sequencing adaptors to each sample using the following PCR program settings: 37°C for 10 min, 95°C for 2 min; 6 cycles of 95°C for 30 s, 54°C for 30 s, 72°C for 120 s; 16 cycles of 95°C for 30 s; 72°C for 2 min; 72°C for 1 min. The TempO-Seq^®^ libraries were pooled (5 μl of all 96 samples) and purified using the Macherey-Nagel NucleoSpin^®^ Gel and PCR Clean-Up kit (Clontech Laboratories Inc., Bethlehem, PA, United States), according to the manufacturer’s instructions for PCR cleanup with three modifications outlined in the TempO-Seq^®^ Assay User Guide (v2.1, 2017). The pooled, purified TempO-Seq^®^ libraries were sequenced using a NextSeq^®^ 500/550 High Output flow cell (v2 kit, 75 cycles) on an Illumina NextSeq^®^ 500 Sequencing platform (Illumina, San Diego, CA, United States).

### Sequencing data preprocessing, alignment, and quality control

Sequencing data have been archived in the National Center for Biotechnology Information (NCBI) Gene Expression Omnibus (GEO) database under accession number GSE196373. Raw sequencing data were analyzed at Health Canada and assigned to respective sample files by demultiplexing them with blc2fastq v2.20.0.422. They were then trimmed for quality control using fastp (v0.20.0). The subsequent FASTQ files were aligned to the TempO-Seq^®^ Human Tox + Surrogate Panel reference sequences (2,977 probes) from BioSpyder using their purpose-built analysis pipeline (TempO-SeqR, v3.0) to generate a table of counts (one per gene per sample). Briefly, the TempO-SeqR pipeline used STAR v2.7.8a to perform alignment of raw reads to the reference sequences. Subsequently, the qCount function of the QuasR package (v1.30.0) was used to produce a gene X sample count matrix of raw counts from the BAM files output by STAR.

Quality control of all study samples was performed on the count matrix using several methods to measure consistency and remove low quality samples, using criteria outlined in Harrill et al. (2021) as a guideline. Samples were removed from the study if they clustered as singletons at a dissimilarity of 0.1 using 1-Spearman correlation using complete linkage. We used a cut-off of 10% of the median number of reads to remove samples that had insufficient sequencing depth. We eliminated any samples outside of Tukey’s Outer Fence (3X interquartile range) for: 1) the number of probes capturing the top 80% of the signal; 2) the Gini coefficient (which measures inequality in distributions); and 3) the number of active probes (those with at least 5 mapped reads). Based on these metrics and suboptimal sequencing depth, nine experimental samples were removed (NIT: one replicate each of DMSO solvent control, 2 μM, 3.9 μM, 15.6 μM; MTZ: one replicate of 2 μM and two replicates of 250 μM; NOV: one replicate each of 2 μM and 125 μM).

### Statistical and bioinformatic analyses for TGx-DDI classification

To account for sequence-to-sequence variability in read depth between the samples, read counts were normalized using DESeq2 (v1.30.1) (Love et al., 2014) using the counts () function in R (R, 2020). Samples with poor data quality were identified through visualization using boxplots and hierarchical cluster analysis. This resulted in the exclusion of nine samples from the TGx-DDI classification analysis due to suboptimal sequencing depth (identified in previous section), or the fact that they clustered as singletons. The TGx-DDI genomic biomarker was used to classify chemicals as DDI or non-DDI using statistical modeling and bioinformatics tools. Detailed analytical information can be found in Yauk et al. (2016) and Buick et al. (2017). Gene Symbols for TGx-DDI biomarker genes that had multiple probes were averaged. Hierarchical clustering was accomplished using the hclust () function in R (www.r-project.org). Agglomerative clustering was based on average linkage with Euclidean distances (Becker et al., 1988). DDI vs. non-DDI classifications were determined using the Nearest Shrunken Centroids (NSC) method (Tibshirani et al., 2002) in the pamr function of R (www.bioconductor.org), as previously described (Yauk et al., 2016; Buick et al., 2017; Li et al., 2017). Briefly, the standardized centroid (SC) was calculated by applying the NSC method for DDI and non-DDI test chemicals in the training set and is the mean expression level for each gene in a class divided by its within-class standard deviation. For each DDI and non-DDI chemical, the SC is shrunken in the direction of the overall centroid to create the NSC. Experimental samples (i.e., exposed and control) were then classified by comparing their gene expression profile to the class of NSCs and then assigned to a class closest to it in squared distance so that the probability of class membership was >0.90 (Li et al., 2015; Li et al., 2017).

Three distinct analyses were performed to classify the compounds as DDI and non-DDI using the TGx-DDI genomic biomarker, including NSC probability analysis (PA; visualized using heatmaps), principal component analysis (PCA), and hierarchical clustering (HC), as outlined in Yauk et al. (2016) and Buick et al. (2017). PCA was completed using the prcomp () function in R (Venables and Ripley, 2002), where the training set data (Li et al., 2015) was used to estimate the principal components (PC). These PC loadings were applied to the data generated with the three test agents. Samples with PC1 <0 were classified as DDI; otherwise, the sample was classified as non-DDI. For the HC analysis, samples clustering with the DDI training compounds were classified as DDI and, similarly, samples clustering with the non-DDI agents were classified as non-DDI. Samples that clustered as singletons (i.e., that cluster on their own) were classified as unknown. A scatterplot generated using data from the TGx-DDI training set and experimental chemicals was generated to visualize the outcomes. Classification was determined as follows: a chemical was classified as DDI if it resulted in a positive call in any one of three classification analyses (NSC PA heatmaps, PCA, or HC); whereas, a chemical was classified as non-DDI if it was negative in all of the three analyses (Yauk et al., 2016; Buick et al., 2017; Buick et al., 2020).

### CometChip^®^ assay

The HT alkaline CometChip^®^ assay was conducted at MIT and was used to assess DNA damage (i.e., single-strand breaks) across the concentration range for each chemical in 96-well plates following a 4 h chemical exposure (Wood et al., 2010). For all CometChip^®^ data, results reflect three independent experiments. Analysis was done at 4 h to align with the gene expression analysis experimental design. Control and exposed TK6 cells were loaded into the CometChip^®^ wells and were allowed to settle by gravitational forces into the microwells, as previously described (Wood et al., 2010; Buick et al., 2021). Briefly, the cells were encased in a 1% low melting point agarose overlay and then lysed in cold lysis buffer (2.5 M NaCl, 100 mM EDTA, 10 mM Tris, pH 10 with 1% Triton X-100 and 10% DMSO) overnight at 4°C. After cell lysis, the CometChip^®^ was equilibrated in alkaline electrophoresis buffer (300 mM NaOH and 1 mM EDTA) for 40 min and then separated for 50 min with a 300 mA current at 4°C. After electrophoresis, the chip was neutralized twice for 15 min in 0.4 M Tris, pH 7.4 at 4°C and then equilibrated overnight in 20 mM Tris, pH 7.4 at 4°C. Following equilibration, the chip was stained in 0.1X SYBR Gold for 30 min and then destained for >1 h in 20 mM Tris, pH 7.4 at 4°C. Once destained, comet images were captured using a Nikon 80i microscope at 4× magnification for all 96 wells on the chip. The tiff images were taken and analyzed using in-house software, as previously described (Wood et al., 2010). All chemical conditions and controls were run in parallel.

### Statistical analysis of CometChip^®^ data

Median % tail DNA was analysed using one-way analysis of variance (ANOVA). The normality assumption was tested using the Anderson-Darling statistic (Stephens, 1986) and the common variance assumption was verified using the Fligner-Killeen test of homogeneity of variances (Conover et al., 1981). If either assumption was not met, the rank transformation was applied and the nonparametric one-way ANOVA was performed (Conover and Iman, 1981). Pairwise contrasts to corresponding vehicle controls were conducted using the *t*-test. The subsequent *p*-values were then FWER (Family-Wise Error Rate) adjusted using the Dunnett’s method.

## Results

### Cytotoxicity assessment and selection of concentrations for testing

**FIGURE 1:**
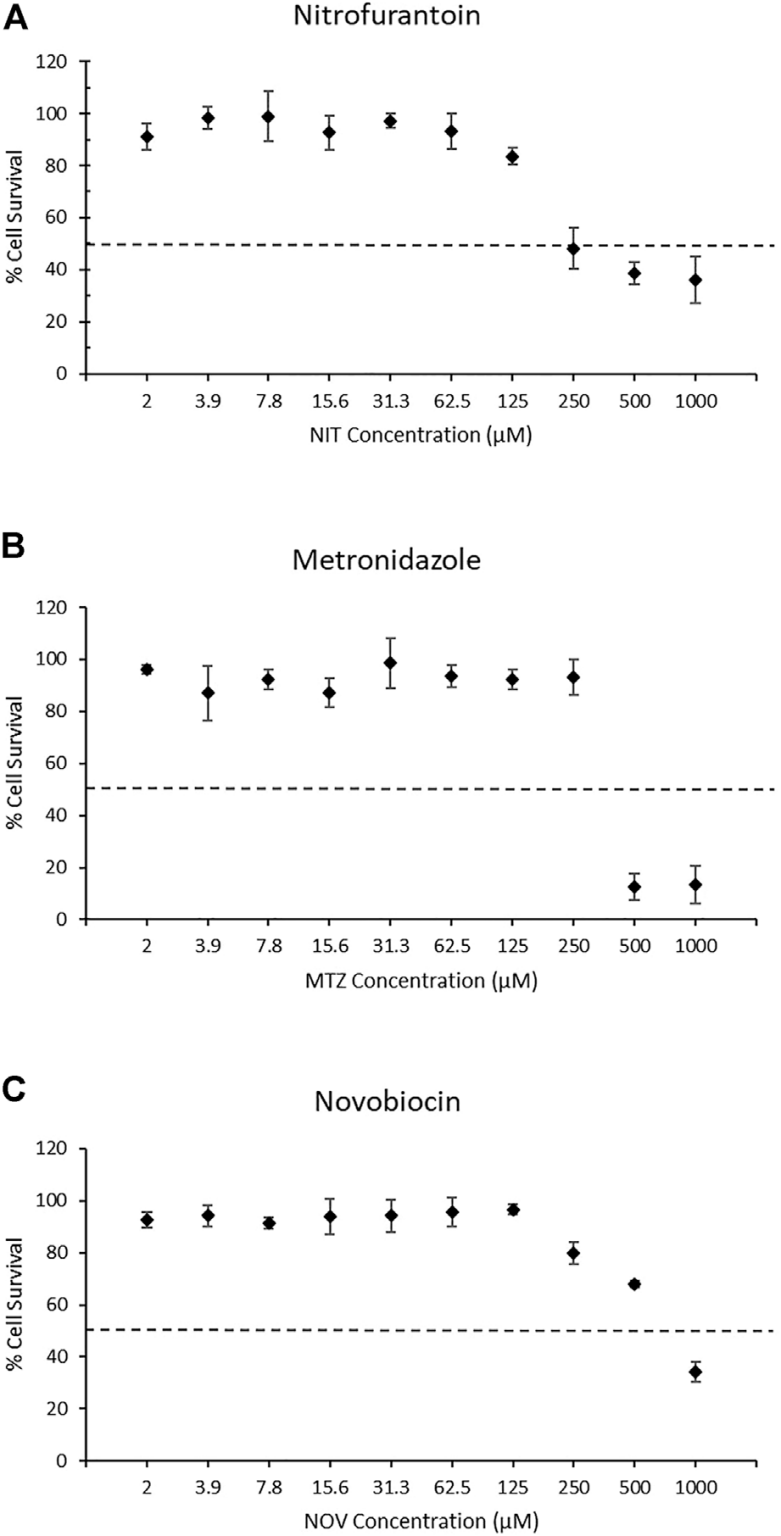
MTT assay results for nitrofurantoin (NIT) **(A)**, metronidazole (MTZ) **(B)**, and novobiocin (NOV) **(C)**. MTT measures cellular metabolic activity as an indicator of cell viability and cytotoxicity for solvent controls and treated TK6 cells (*n* = 3). The dashed line represents the cytotoxicity threshold of 50% cell viability relative to each chemical’s matched vehicle control. Dimethyl sulfoxide, acetic acid and water were used as vehicle controls for NIT, MTZ, and NOV, respectively. Error bars represent the coefficient of variation. Cytotoxic concentrations were eliminated from the gene expression analysis for TGx-DDI classification (i.e., 500 and 1,000 μM for NIT and MTZ, and 1,000 μM for NOV). All concentrations were run for CometChip^®^ analysis.

To select the appropriate concentration range for detection of the DNA damage response using the TGx-DDI biomarker and DNA damage using the CometChip^®^ assay, we first evaluated cytotoxicity using the colorimetric MTT assay following a 4 h exposure + 20 h recovery to ten concentrations of each drug in triplicate (2 μM–1,000 μM). In this assay, cellular metabolic activity provides an indication of cell viability and cytotoxicity in treated TK6 cells (Figure 1). The two highest concentrations of NIT and MTZ (500 and 1,000 μM) caused declines in viability beyond the threshold of 50% cytotoxicity (Figures 1A,B, respectively). Only the highest concentration of NOV tested (1,000 μM) caused a reduction in cell viability in excess of the cytotoxicity cut-off of 50% (Figure 1C). The 250 μM concentration of NIT, in addition to the 250 μM and the 500 μM concentrations of NOV, caused declines in cell viability but did not surpass the 50% threshold. The remaining concentrations tested for all three pharmaceutical agents did not cause any notable declines in cell viability (Figure 1). All concentrations of test chemicals were used for CometChip^®^ analysis, including those causing excessive cytotoxicity. The drug treatments exceeding the cytotoxicity limits were excluded from TGx-DDI analysis by nCounter^®^ and TempO-Seq^®^ (i.e., 500 and 1,000 μM for NIT and MTZ, and 1,000 μM for NOV) (Ellinger-Ziegelbauer et al., 2009).

### Evaluation of the DNA damage response using the TGx-DDI biomarker

**FIGURE 2:**
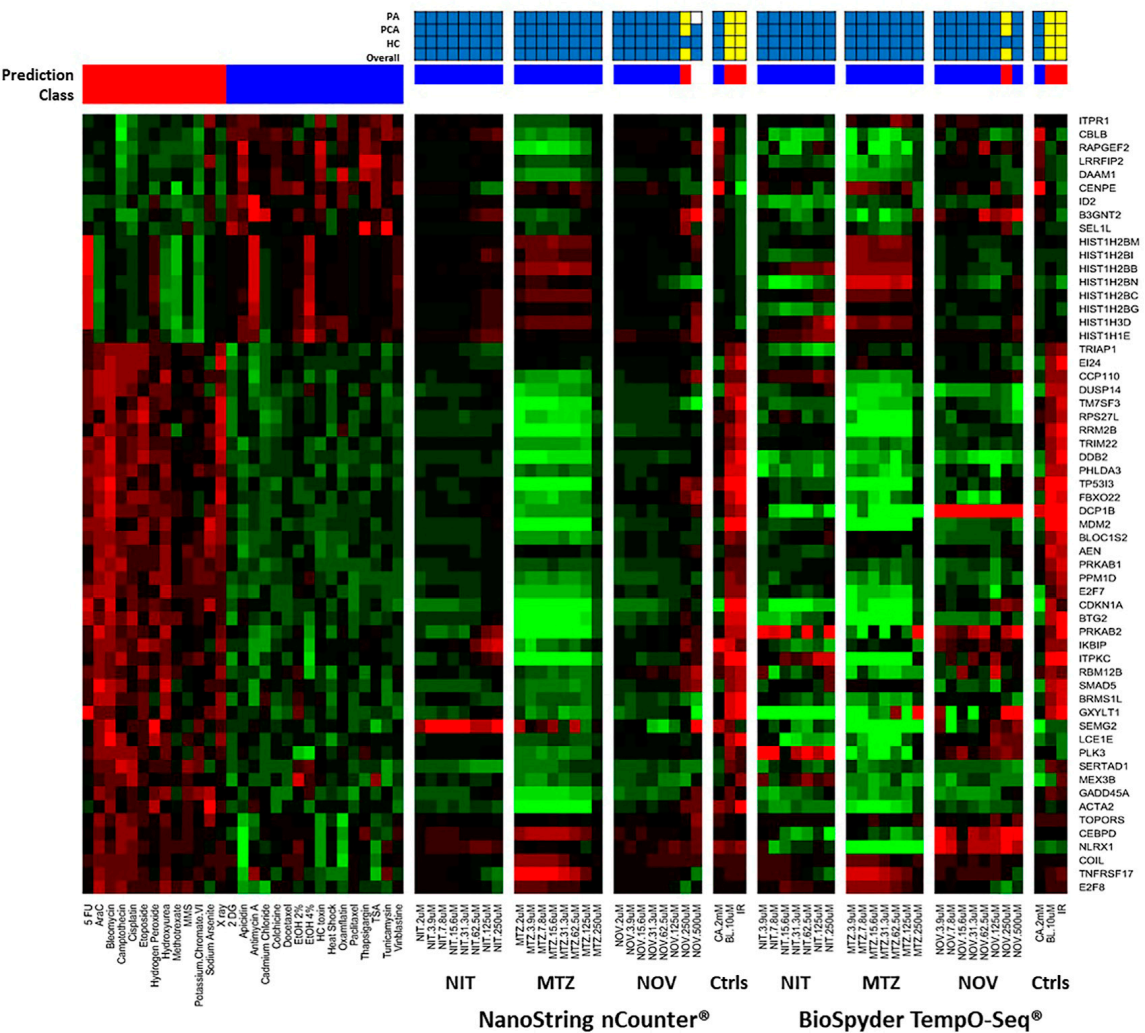
TGx-DDI classification by nCounter^®^ and TempO-Seq^®^ analysis for nitrofurantoin (NIT), metronidazole (MTZ), and novobiocin (NOV). The heatmap on the left depicts the gene expression profiles of the 28 reference chemicals used to generate the biomarker. The test chemicals assessed using nCounter^®^ and TempO-Seq^®^ gene expression technologies in human TK6 cells are shown in the subsequent heatmaps (columns). Gene Symbols corresponding to the GenBank accession numbers for the TGx-DDI biomarker genes are on the right y-axis. The colour scale indicates gene expression fold changes relative to control: up-regulated genes are red, down-regulated genes are green, and genes that are not altered are black. TGx-DDI classification probabilities for all treatment conditions are shown using red (DDI) and blue (non-DDI) bars above each heatmap. Caffeine (CA), bleomycin (BL) and ionizing radiation (IR) were used as negative and positive controls, respectively. Cytotoxic concentrations were not analyzed. The grids above the heatmaps indicate the results of the three different TGx-DDI analyses: Probability Analysis (PA, based on Nearest Shrunken Centroid Analysis), Principal Component Analysis (PCA) and Hierarchical Clustering (HC). The overall call is DDI if any one of these analyses yields a DDI call. Yellow boxes indicate a positive DDI classification, blue denotes a negative non-DDI classification and white signifies an unclassified response (i.e., does not yield a DDI or non-DDI call). Sample size was *n* = 3, except for NIT DMSO solvent control, 2 μM NIT, 3.9 μM NIT, 15.6 μM NIT, 2 μM MTZ, 2 μM NOV, and 125 μM NOV that had an n = 2 and 250 μM MTZ that had an *n* = 1.

We used two different gene expression methods to measure the TGx-DDI biomarker genes: NanoString nCounter^®^ digital quantification and BioSpyder TempO-Seq^®^ S1500+ sequencing (Figure 2). The negative assay controls (1X TempO-Seq^®^ Lysis buffer, no lysates) had low mapped read counts (i.e., <1,200) as expected and the positive assay controls (Human Reference Total RNA and Human Brain Total RNA) showed Pearson correlation coefficients between the replicates that were >0.98 for all pairwise comparisons (data not shown). The negative control (2 mM CA) classified as non-DDI using both gene expression technologies (Figure 2, Supplementary Figures 1D,2A, while the two positive controls (10 μM BL and 4 Gy IR) classified as DDI in both assays (Figure 2, Supplementary Figures 1D,2B-C). After outlier analysis, the final sample size was n = 3, except for NIT DMSO solvent control, 2 μM NIT, 3.9 μM NIT, 15.6 μM NIT, 2 μM MTZ, 2 μM NOV, and 125 μM NOV that had an *n* = 2 and 250 μM MTZ that had an *n* = 1.

A three-pronged analysis that includes NSC PA, PCA, and HC was used for classification (Li et al., 2017). If a drug had a positive outcome in one or more analyses, it was predicted to be DDI; whereas, a chemical that had a negative outcome in all three analyses was classified as non-DDI. The heatmap (Figure 2) shows the gene expression profiles and the TGx-DDI classifications for the three test compounds using nCounter^®^ and TempO-Seq^®^ gene expression analysis. Supplementary Figure 1A–C depict the PC analyses for NIT, MTZ, and NOV, respectively; whereas, the HC analyses are shown in Supplementary Figures 3A–H for NIT, Supplementary Figures 4A–H for MTZ, and Supplementary Figures 5A–I for NOV. The TGx-DDI biomarker classified all concentrations of NIT and MTZ as non-DDI using both gene expression technologies. NOV was also classified as non-DDI at all concentrations except 250, which was DDI using both nCounter^®^ and TempO-Seq^®^ technologies. The 500 μM concentration could not be classified (probability of class membership <0.9 for both DDI and non-DDI calls) using the TGx-DDI with nCounter^®^ data but was identified as non-DDI using TempO-Seq^®^ data. Overall, the TGx-DDI classification results were highly comparable across laboratories and technologies.

### Quantification of DNA strand breaks using the CometChip^®^ assay

**FIGURE 3:**
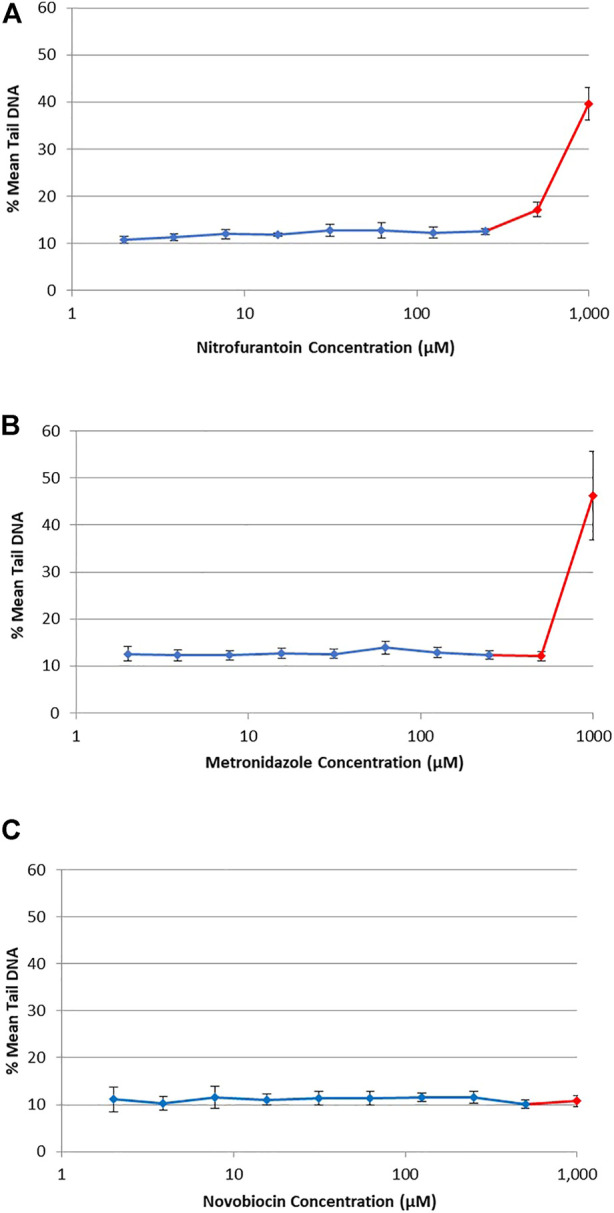
DNA damage in human TK6 cells measured using the alkaline CometChip^®^ assay. Cells were exposed to increasing concentrations of NIT **(A)**, MTZ **(B)**, and NOV **(C)** from 2 μM to 1,000 μM. Mean % tail DNA is shown following 4 h exposures. The data are expressed as mean % tail DNA ±SD (*n* = 3, for three experiments run in duplicate on different days). Cytotoxic concentrations are shown in red (>50% cell death compared to matched solvent control).

DNA damage (i.e., single-strand breaks) was quantified in human TK6 cells following a 4 h exposure to three antibiotic drugs using the alkaline CometChip^®^ assay (Figure 3). Hydrogen peroxide (25–100 μM), used as a positive control for the CometChip^®^ assay, showed a positive concentration-response (data not shown). Chemicals were considered positive if there was an increase in mean % tail DNA that was statistically significant compared to matched solvent control for non-cytotoxic test concentrations (*p* < 0.05). There was no accumulation of DNA damage observed at non-cytotoxic concentrations for NIT, MTZ, or NOV, compared to their matched vehicle controls (Figure 3A for NIT, Figure 3B for MTZ and Figure 3C for NOV, respectively). DNA damage was only observed for each test compound at overtly cytotoxic concentrations shown in red in Figure 3 (i.e., 500 μM and 1,000 μM for NIT and MTZ, and 1,000 μM for NOV). As cytotoxicity is associated with potential DNA damage and/or degradation, it is critical that the highest concentration tested using the Comet assay does not induce excessive cell death (Tice et al., 2000). As such, the positive CometChip^®^ results at overtly cytotoxic concentrations were not considered relevant in the final analysis. Overall, there was no evidence for single strand breaks following exposure to NIT, MTZ, or NOV, except at highly cytotoxic concentrations (>50% cell death).

## Discussion

The TGx-DDI transcriptomic biomarker was designed to improve test specificity and provide mechanistic context to enhance genotoxic hazard assessment of drug candidates for pharmaceutical development and of industrial and environmental chemicals. It can also be used to provide context to conflicting genotoxicity data to determine human relevance. The primary objective of this case study was to use the TGx-DDI biomarker to classify three well-known anti-infective agents (i.e., NIT, MTZ, and NOV) as DDI or non-DDI, followed by the CometChip^®^ assay to corroborate the TGx-DDI classifications, in an integrated test strategy to provide clarity on conflicting genotoxicity data to determine human relevance. A secondary objective was to further validate the TGx-DDI biomarker across gene expression technologies and laboratories to provide further evidence to the robustness and added value of this tool for modern toxicity testing.

NIT was predicted to be non-DDI at all non-cytotoxic test concentrations using the TGx-DDI biomarker with both nCounter^®^ and TempO-Seq^®^ data, and showed no evidence of DNA damage using the CometChip^®^ assay. NIT is an oral antibiotic of choice in the treatment of bladder infections caused by many Gram-negative (e.g., *Escherichia coli*) and some Gram-positive bacteria (e.g., *Staphylococcus saprophyticus*) with a long history of safe use in animals and humans, including pregnant women before 38 weeks of gestation (Muller et al., 2017). NIT was first introduced to the market in 1953 and is on the World Health Organization’s list of essential medicines (Blass, 2015; World Health Organization, 2019). It was ranked 192 out of the top 300 drugs of 2019 with an estimated almost three million prescriptions in the US alone (ClinCalc DrugStats Database, 2019). NIT is converted by the action of bacterial nitroreductases to reactive intermediates which can inactivate or alter bacterial ribosomal proteins and other macromolecules. This inhibits vital biochemical processes in bacterial cells such as DNA, RNA, protein, and cell wall synthesis, and aerobic energy metabolism (Race et al., 2005; Roldán et al., 2008). NIT is mutagenic in the Ames assay due to activation by bacterial nitroreductases (not relevant in mammalian cells), which calls into question the human relevance of these mutagenicity results (Olive and McCalla, 1977; Ni et al., 1987; Mokdad-Bzeouich et al., 2015). NIT has also shown mutagenicity in mammalian cells, including CHO cells and human lymphocytes (Gao et al., 1989; Slapsyte et al., 2002) and in the kidney of Big Blue transgenic mice (Quillardet et al., 2006). Additionally, NIT induced sister chromatid exchanges (SCEs), CAs and MN in the bone marrow of rodents (Parodi et al., 1983; Thompson, 1986). However, there is inadequate and limited evidence as to the carcinogenic potential of NIT in humans and animals, respectively, especially when considering the long history of human use. Indeed, the International Agency for Research on Cancer (IARC) concluded that NIT’s carcinogenicity to humans is not classifiable (i.e., group 3) (IARC, World Health Organization, 1990). In humans, there is no evidence for increased SCE in lymphocytes in adult patients with UTI treated with NIT (Sardaş et al., 1990); however, there is some evidence of a significant increase in SCE rates in lymphocytes of pediatric patients whose blood was sampled before and after treatment (Slapsyte et al., 2002). Despite the conflicting evidence for the genetic safety of NIT, the relevance of the experimental test systems and conditions have been questioned, as long-term antimicrobial treatment with NIT is generally considered safe after more than 60 years of human use (Uhari et al., 1996). Thus, this integrated test strategy combining the TGx-DDI biomarker and the CometChip^®^ assay mechanistically supports the genetic safety of NIT for human use and highlights the irrelevant nature of the conflicting genotoxicity.

MTZ was also classified as non-DDI at all non-cytotoxic concentrations using the TGx-DDI biomarker (with both gene expression technologies), which was confirmed using the CometChip^®^ assay. MTZ is an antibacterial and antiprotozoal agent used to treat anaerobic bacterial infections, such as endocarditis and bacterial vaginosis, as well as protozoal infections, such as trichomoniasis, amebiasis, and giardiasis, in human and veterinary applications. MTZ was used commercially as of 1960 and is also on the World Health Organization’s list of essential medicines (Li and Corey, 2013; World Health Organization, 2019). Of the top 300 drugs of 2019, MTZ was ranked 138th with more than four-and-a-half million prescriptions estimated in the US alone (ClinCalc DrugStats Database, 2019). Un-ionized MTZ readily enters the cell by passive diffusion and is activated in the cytoplasm of susceptible anaerobic organisms and cells. MTZ is selective for anaerobic bacteria as these organisms have the ability to reduce MTZ to its active form intracellularly. Following nitroreductive biotransformation, MTZ can yield DNA-damaging reactive species (Martelli et al., 1990). Reduced MTZ and free radicals can interact with DNA and inhibit DNA synthesis and degradation, resulting in bacterial cell death (Prescribers’ Digital Reference Network, 2021). MTZ has demonstrated mutagenic activity in the Ames bacterial reverse mutation assay, both induced by the drug itself (Melo and Ferreira, 1990) and by the urine of treated patients (Speck et al., 1976; Connor et al., 1977). In mammalian cells, conflicting evidence exists as to the genotoxicity of MTZ. Some studies show that MTZ exposure leads to loss of DNA helix content, DNA strand breakage, unscheduled DNA synthesis and SCEs; whereas, other studies do not reveal any genotoxicity associated with MTZ (Connor et al., 1977; Lambert et al., 1979; Martelli et al., 1990; Reitz et al., 1991; Ré et al., 1997; Menéndez et al., 2001; Buschini et al., 2009). Conflicting evidence also exists following MTZ treatment for *in vivo* studies in that some demonstrate the genotoxic nature of MTZ, while others studies do not support the genotoxicity of MTZ or cannot be clearly interpreted (Buschini et al., 2009). Human studies have failed to sufficiently demonstrate the potential for genetic damage. However, there is evidence that MTZ’s biological activity may be reliant on anaerobic environments (Rosenkranz et al., 1976; Dobiás et al., 1994). While there is inadequate evidence to support the carcinogenic potential of MTZ in humans, sufficient evidence does exist in mice and rats. As such, MTZ is classified as possibly carcinogenic to humans (i.e., group 2B; (IARC, World Health Organization, 1990). Despite the conflicting genotoxic profile of MTZ, it is likely that relevance to humans is questionable given the long history of safe therapeutic use. This case study adds to the weight of evidence supporting the genetic safety of MTZ for human use.

NOV was classified as non-DDI for the 2 μM–125 μM concentrations using the TGx-DDI biomarker with the nCounter^®^ and TempO-Seq^®^ technologies. The 250 μM concentration classified as DDI using both technologies, although the PA and PCA positive TGx-DDI call at this concentration were borderline positive and NOV was non-DDI at all concentrations for the HC analysis. The 500 μM concentration was unclassified using the TGx-DDI biomarker with nCounter^®^ data and was non-DDI with TempO-Seq^®^ data. The CometChip^®^ assay was negative at all concentrations tested. NOV, also known as albamycin, streptonivcin, or cathomycin, is a narrow-spectrum aminocoumarin antibiotic drug that is mostly active against Gram-positive and certain Gram-negative bacteria (Committee for Veterinary Medicinal Products, 1999). NOV was widely used as a human therapeutic in the past; in the 1960s, this antibiotic was licensed for clinical use, but the oral NOV formula has since been taken off the market due to poor efficacy (Food and Drug Administration, 2011). It is still used in veterinary applications for the treatment of bovine mastitis in lactating and dry-off cows (Committee for Veterinary Medicinal Products, 1999). NOV is a bacterial DNA-gyrase inhibitor that also acts as a topoisomerase II (topo II) inhibitor at high concentrations (Maxwell, 1993; Heide, 2009a). NOV interacts directly with the B subunit (GyrB) of the enzyme and acts as a competitive inhibitor of the ATPase reaction catalysed by GyrB, which inhibits ATP-dependent supercoiling of DNA (Maxwell, 1993; Committee for Veterinary Medicinal Products, 1999; Heide, 2009b). Significantly higher concentrations (i.e., 1,000-fold) are required to similarly inhibit topo II, the equivalent enzyme in mammalian cells (Committee for Veterinary Medicinal Products, 1999). NOV also inhibits DNA repair synthesis in some human cells *in vitro* (e.g., lymphocytes and fibroblasts), whereas other mammalian cell lines are resistant (e.g., human keratinocytes and Chinese hamster ovary cells). Effects on DNA excision repair are the result of a non-specific effect on ATP metabolism (Committee for Veterinary Medicinal Products, 1999). It is a non-specific inhibitor of DNA topoisomerase II (DNA topoII), which may trigger a positive TGx-DDI signal at higher concentrations, as seen with the 250 μM concentration. Thus, our results indicate that this compound may be weakly DDI; the weak response may be due to a difference in the binding constant for topoII compared to other bacterial gyrase inhibitors (Savoldi-Barbosa et al., 1999). Indeed, two other bacterial gyrase inhibitors with topoII inhibitory activity (i.e., ciprofloxacin and norfloxacin) that were previously tested using the TGx-DDI biomarker yielded conflicting results with respect to classification. Specifically, ciprofloxacin classified as DDI in two of the three analyses; whereas, norfloxacin classified as non-DDI in all three analyses. Li et al. (2017) proposed that differences in affinity to mammalian topoII leads to positive results or conflicting results of these drugs only at higher concentrations. Furthermore, NOV has been shown to cause concentration-dependent DNA fragmentation that precedes apoptosis in primary cultures of mouse thymocytes, by inhibiting DNA-rejoining activity of the enzyme (Onishi et al., 1993). While NOV administration initially demonstrated little toxicity (Kirby et al., 1956), it has since been withdrawn from the market for human therapeutic use due to poor pharmacological properties and safety concerns regarding liver toxicity (Shirude and Hameed, 2012). Despite the experimental evidence demonstrating that NOV may be genotoxic in some test systems and conditions, the present integrated test strategy suggests that NOV may be weakly genotoxic only at high concentrations that are above therapeutic exposure levels.

Initial development and validation of the TGx-DDI genomic biomarker was completed in human TK6 cells using Agilent microarray technology (Buick et al., 2015; Li et al., 2015; Williams et al., 2015; Yauk et al., 2016; Li et al., 2017). The biomarker has since been validated using other gene expression platforms, including qPCR (Cho et al., 2019), NanoString nCounter^®^ digital counting (Li et al., 2017) and RNA-Sequencing (Buick et al., 2020; Buick et al., 2021). Further validation demonstrated the effectiveness of the TGx-DDI biomarker using metabolically competent human HepaRG™ cells (Buick et al., 2015; Buick et al., 2020; Buick et al., 2021). In this case study, we provide further confirmation as to the robustness of the TGx-DDI biomarker as it rendered nearly identical TGx-DDI classifications across gene expression technologies (i.e., nCounter^®^ vs. TempO-Seq^®^), and laboratories, as the nCounter^®^ work was completed at Georgetown University and the TempO-Seq^®^ work was completed at Health Canada.

In conclusion, this case study supports the utility of the TGx-DDI transcriptomic biomarker in conjunction with the CometChip^®^ assay in evaluating the human relevance of complex findings in genetic testing battery. The approach provides mechanistic insight from a transcriptomic-based NAM anchored against the measurement of conventional endpoints in a modernized assay format to inform the potential for adverse health effects. It demonstrates how these two genetic toxicology assays may be integrated into a single experimental design. In addition, this case study provides further evidence to the genetic safety of NIT, MTZ, and NOV for human and veterinary applications.

## Data Availability

The datasets presented in this study can be found in online repositories. The names of the repository/repositories and accession numbers can be found below: https://www.ncbi.nlm.nih.gov/geo/, GSE196373.

## Abbreviations

CA, Chromosome Aberrations; DDI, DNA Damage-Inducing; HESI, Health and Environmental Sciences Institute; HC, Hierarchical Clustering; HT, High-Throughput; MN, Micronuclei; MoA, Mode of Action; MTZ, Metronidazole; NIT, Nitrofurantoin; Non-DDI, Non-DNA Damage-Inducing; NOV, Novobiocin; NSC, Nearest Shrunken Centroids; PCA, Principal Component Analysis; SCE, Sister Chromatid Exchange; SSBs, Single-Strand Breaks; TGx, Toxicogenomics; UTI, Urinary Tract Infection.

## References

[B1] Adeleye Y. Andersen M. Clewell R. Davies M. Dent M. Edwards S. (2015). Implementing Toxicity Testing in the 21st Century (TT21C): Making safety decisions using toxicity pathways, and progress in a prototype risk assessment. Toxicology 332, 102–111. 10.1016/j.tox.2014.02.007 24582757

[B2] Becker R. A. Chambers J. M. Wilks A. R. (1988). The new S language: A programming environment for data analysis and graphics. Wadsworth & Brooks/Cole.

[B3] Blass B. E. (2015). Basic principles of drug discovery and development. Elsevier.

[B4] Buick J. K. Moffat I. Williams A. Swartz C. D. Recio L. Hyduke D. R. (2015). Integration of metabolic activation with a predictive toxicogenomics signature to classify genotoxic versus nongenotoxic chemicals in human TK6 cells. Environ. Mol. Mutagen. 56, 520–534. 10.1002/em.21940 25733247 PMC4506226

[B5] Buick J. K. Williams A. Gagné R. Swartz C. D. Recio L. Ferguson S. S. (2020). Flow cytometric micronucleus assay and TGx-DDI transcriptomic biomarker analysis of ten genotoxic and non-genotoxic chemicals in human HepaRG™ cells. Genes Environ. 42, 5. 10.1186/s41021-019-0139-2 32042365 PMC7001283

[B6] Buick J. K. Williams A. Kuo B. Wills J. W. Swartz C. D. Recio L. (2017). Integration of the TGx-28.65 genomic biomarker with the flow cytometry micronucleus test to assess the genotoxicity of disperse orange and 1, 2, 4-benzenetriol in human TK6 cells. Mutat. Res. 806, 51–62. 10.1016/j.mrfmmm.2017.09.002 29017062

[B7] Buick J. K. Williams A. Meier M. J. Swartz C. D. Recio L. Gagné R. (2021). A modern genotoxicity testing paradigm: Integration of the high-throughput CometChip® and the TGx-DDI transcriptomic biomarker in human HepaRG™ cell cultures. Front. Public Health 9, 694834. 10.3389/fpubh.2021.694834 34485225 PMC8416458

[B8] Buschini A. Ferrarini L. Franzoni S. Galati S. Lazzaretti M. Mussi F. (2009). Genotoxicity revaluation of three commercial nitroheterocyclic drugs: Nifurtimox, benznidazole, and metronidazole. J. Parasitol. Res. 2009, 463575. 10.1155/2009/463575 20981287 PMC2963127

[B9] Chen R. Lin Y. T. Fornace A. J. Li H. H. (2022). A high-throughput and highly automated genotoxicity screening assay. Altex 39 (1), 71–81. 10.14573/altex.2102121 34585733

[B10] Cho E. Buick J. K. Williams A. Chen R. Li H. H. Corton J. C. (2019). Assessment of the performance of the TGx-DDI biomarker to detect DNA damage-inducing agents using quantitative RT-PCR in TK6 cells. Environ. Mol. Mutagen. 60, 122–133. 10.1002/em.22257 30488505 PMC6588084

[B11] Choudhuri S. Patton G. W. Chanderbhan R. F. Mattia A. Klaassen C. D. (2018). From classical toxicology to Tox21: Some critical conceptual and technological advances in the molecular understanding of the toxic response beginning from the last quarter of the 20th century. Toxicol. Sci. 161, 5–22. 10.1093/toxsci/kfx186 28973688 PMC5837539

[B12] ClinCalc DrugStats Database (2019). The top 300 of 2019, 2021.

[B13] Committee for Veterinary Medicinal Products (1999). The European Agency for the Evaluation of Medicinal Products, Veterinary Medicines and Inspections: Novobiocin summary report. Available at: https://www.ema.europa.eu/en/documents/mrl-report/novobiocin-summary-report-committee-veterinary-medicinal-products_en.pdf .

[B14] Connor T. H. Stoeckel M. Evrard J. Legator M. S. (1977). The contribution of metronidazole and two metabolites to the mutagenic activity detected in urine of treated humans and mice. Cancer Res. 37, 629–633. 318924

[B15] Conover W. J. Iman R. L. (1981). Rank transformations as a bridge between parametric and nonparametric statistics. Am. Statistician 35 (3), 124–129. 10.2307/2683975

[B16] Conover W. J. Johnson M. E. Johnson M. M. (1981). A comparative study of tests for homogeneity of variances, with applications to the outer continental shelf bidding data. Technometrics 23 (4), 351–361. 10.1080/00401706.1981.10487680

[B17] Corton J. C. Williams A. Yauk C. L. (2018). Using a gene expression biomarker to identify DNA damage-inducing agents in microarray profiles. Environ. Mol. Mutagen. 59, 772–784. 10.1002/em.22243 30329178 PMC7875442

[B18] Dearfield K. L. Gollapudi B. B. Bemis J. C. Benz R. D. Douglas G. R. Elespuru R. K. (2017). Next generation testing strategy for assessment of genomic damage: A conceptual framework and considerations. Environ. Mol. Mutagen. 58, 264–283. 10.1002/em.22045 27650663

[B19] Dobiás L. Cerná M. Rössner P. Srám R. (1994). Genotoxicity and carcinogenicity of metronidazole. Mutat. Research/Reviews Genet. Toxicol. 317, 177–194. 10.1016/0165-1110(94)90001-9 7515153

[B20] Ellinger-Ziegelbauer H. Aubrecht J. Kleinjans J. C. Ahr H. J. (2009). Application of toxicogenomics to study mechanisms of genotoxicity and carcinogenicity. Toxicol. Lett. 186 (1), 36–44. 10.1016/j.toxlet.2008.08.017 18822359

[B21] Food and Drug Administration (2011). Determination that ALBAMYCIN (novobiocin sodium) capsule, 250 milligrams, was withdrawn from sale for reasons of safety or effectiveness. Available at: https://www.federalregister.gov/documents/2011/01/19/2011-1000/determination-that-albamycin-novobiocin-sodium-capsule-250-milligrams-was-withdrawn-from-sale-for .

[B22] Galloway S. M. (2017). International regulatory requirements for genotoxicity testing for pharmaceuticals used in human medicine, and their impurities and metabolites. Environ. Mol. Mutagen. 58, 296–324. 10.1002/em.22077 28299826

[B23] Gao N. Ni Y. C. Thornton-Manning J. R. Fu P. P. Heflich R. H. (1989). Mutagenicity of nitrofurantoin and furazolidone in Chinese hamster ovary cell strains. Mutat. Res. 225, 181–187. 10.1016/0165-7992(89)90117-6 2927439

[B24] Geiss G. K. Bumgarner R. E. Birditt B. Dahl T. Dowidar N. Dunaway D. L. (2008). Direct multiplexed measurement of gene expression with color-coded probe pairs. Nat. Biotechnol. 26 (3), 317–325. 10.1038/nbt1385 18278033

[B25] Harrill J. A. Everett L. J. Haggard D. E. Sheffield T. Bundy J. Willis C. M. (2021). High-throughput transcriptomics platform for screening environmental chemicals. Toxicol. Sci. 181 (1), 68–89. 10.1093/toxsci/kfab009 33538836 PMC10194851

[B26] Heide L. (2009a). Aminocoumarins mutasynthesis, chemoenzymatic synthesis, and metabolic engineering. Methods Enzymol. 459, 437–455. 10.1016/S0076-6879(09)04618-7 19362650

[B27] Heide L. (2009b). The aminocoumarins: Biosynthesis and biology. Nat. Prod. Rep. 26, 1241–1250. 10.1039/b808333a 19779639

[B28] International Agency for Research on Cancer (IARC), World Health Organization (1990). IARC monographs on the evaluation of carcinogenic risks to humans: Pharmaceutical drugs, 50. Available at: https://publications.iarc.fr/68 .

[B29] International Conference on Harmonisation (ICH) (2012). “Guidance on genotoxicity testing and data interpretation for pharmaceuticals intended for human use,” in ICH harmonised tripartite guideline. S2(R1). Available from: https://database.ich.org/sites/default/files/S2_R1_Guideline.pdf . 22675782

[B30] Kirby W. M. Hudson D. G. Noyes W. D. (1956). Clinical and laboratory studies of novobiocin, a new antibiotic. AMA. Arch. Intern. Med. 98, 1–7. 10.1001/archinte.1956.00250250007001 13338887

[B31] Krewski D. Andersen M. E. Tyshenko M. G. Krishnan K. Hartung T. Boekelheide K. (2020). Toxicity testing in the 21st century: Progress in the past decade and future perspectives. Arch. Toxicol. 94, 1–58. 10.1007/s00204-019-02613-4 31848664

[B32] Lambert B. Lindblad A. Ringborg U. (1979). Absence of genotoxic effects of metronidazole and two of its urinary metabolites on human lymphocytes *in vitro* . Mutat. Res. 67 (3), 281–287. 10.1016/0165-1218(79)90022-3 481453

[B33] Li H. H. Chen R. Hyduke D. R. Williams A. Frotschl R. Ellinger-Ziegelbauer H. (2017). Development and validation of a high-throughput transcriptomic biomarker to address 21st century genetic toxicology needs. Proc. Natl. Acad. Sci. U. S. A. 114, E10881–E10889. 10.1073/pnas.1714109114 29203651 PMC5754797

[B34] Li H. H. Hyduke D. R. Chen R. Heard P. Yauk C. L. Aubrecht J. (2015). Development of a toxicogenomics signature for genotoxicity using a dose-optimization and informatics strategy in human cells. Environ. Mol. Mutagen. 56, 505–519. 10.1002/em.21941 25733355 PMC4506269

[B35] Li H. H. Yauk C. L. Chen R. Hyduke D. R. Williams A. Frötschl R. (2019). TGx-DDI, a transcriptomic biomarker for genotoxicity hazard assessment of pharmaceuticals and environmental chemicals. Front. Big Data 2, 36. 10.3389/fdata.2019.00036 33693359 PMC7931968

[B36] Li J. J. Corey E. J. (2013). Drug discovery: Practices, processes and perspectives. New Jersey, USA: John Wiley & Sons.

[B37] Love M. I. Huber W. Anders S. (2014). Moderated estimation of fold change and dispersion for RNA-seq data with DESeq2. Genome Biol. 15, 550. 10.1186/s13059-014-0550-8 25516281 PMC4302049

[B38] Lynch A. M. Sasaki J. C. Elespuru R. Jacobson-Kram D. Thybaud V. De Boeck M. (2011). New and emerging technologies for genetic toxicity testing. Environ. Mol. Mutagen. 52, 205–223. 10.1002/em.20614 20740635

[B39] Martelli A. Allavena A. Robbiano L. Mattioli F. Brambilla G. (1990). Comparison of the sensitivity of human and rat hepatocytes to the genotoxic effects of metronidazole. Pharmacol. Toxicol. 66, 329–334. 10.1111/j.1600-0773.1990.tb00758.x 2371238

[B40] Maxwell A. (1993). The interaction between coumarin drugs and DNA gyrase. Mol. Microbiol. 9, 681–686. 10.1111/j.1365-2958.1993.tb01728.x 8231802

[B41] Melo M. E. Ferreira L. C. (1990). Screening the mutagenic activities of commonly used antiparasite drugs by the Simultest, a simplified Salmonella/microsome plate incorporation assay. Rev. Inst. Med. Trop. Sao Paulo 32, 269–274. 10.1590/s0036-46651990000400006 2101520

[B42] Menéndez D. Rojas E. Herrera L. A. López M. C. Sordo M. Elizondo G. (2001). DNA breakage due to metronidazole treatment. Mutat. Res. 478, 153–158. 10.1016/s0027-5107(01)00136-1 11406179

[B43] Mokdad-Bzeouich I. Kilani-Jaziri S. Mustapha N. Bedoui A. Ghedira K. Chekir-Ghedira L. (2015). Evaluation of the antimutagenic, antigenotoxic, and antioxidant activities of Eriobotrya japonica leaves. Pharm. Biol. 53, 1786–1794. 10.3109/13880209.2015.1008145 25880139

[B44] Muller A. E. Verhaegh E. M. Harbarth S. Mouton J. W. Huttner A. (2017). Nitrofurantoin's efficacy and safety as prophylaxis for urinary tract infections: A systematic review of the literature and meta-analysis of controlled trials. Clin. Microbiol. Infect. 23, 355–362. 10.1016/j.cmi.2016.08.003 27542332

[B45] National Center for Biotechnology Information (NCBI) (2022). PubChem compound summary for CID 4173, metronidazole. Available at: https://pubchem.ncbi.nlm.nih.gov/compound/Metronidazole .

[B46] National Research Council (2007). “Toxicity testing in the 21st century: A vision and a strategy,” in Anonymous (Washington, D.C.: The National Academies Press).

[B47] Nesslany F. (2017). The current limitations of *in vitro* genotoxicity testing and their relevance to the *in vivo* situation. Food Chem. Toxicol. 106, 609–615. 10.1016/j.fct.2016.08.035 27591928

[B48] Ni Y. C. Heflich R. H. Kadlubar F. F. Fu P. P. (1987). Mutagenicity of nitrofurans in *Salmonella typhimurium* TA98, TA98NR and TA98/1, 8-DNP6. Mutat. Res. 192, 15–22. 10.1016/0165-7992(87)90120-5 3309641

[B49] Olive P. L. McCalla D. R. (1977). Cytotoxicity and DNA damage to mammalian cells by nitrofurans. Chem. Biol. Interact. 16, 223–233. 10.1016/0009-2797(77)90131-4 849625

[B50] Onishi Y. Azuma Y. Sato Y. Mizuno Y. Tadakuma T. Kizaki H. (1993). Topoisomerase inhibitors induce apoptosis in thymocytes. Biochim. Biophys. Acta 1175, 147–154. 10.1016/0167-4889(93)90017-j 8380339

[B51] Organisation for Economic Co-operation and Development (OECD) (2016). “Test No. 487: *In vitro* mammalian cell micronucleus test,” in OECD guidelines for the testing of chemicals (Paris: OECD Publishing).

[B52] Parodi S. Pala M. Russo P. Balbi C. Abelmoschi M. L. Taningher M. (1983). Alkaline DNA fragmentation, DNA disentanglement evaluated viscosimetrically and sister chromatid exchanges, after treatment *in vivo* with nitrofurantoin. Chem. Biol. Interact. 45, 77–94. 10.1016/0009-2797(83)90044-3 6872102

[B53] Phillips D. H. Arlt V. M. (2009). Genotoxicity: Damage to DNA and its consequences. EXS 99, 87–110. 10.1007/978-3-7643-8336-7_4 19157059

[B54] Prescribers’ Digital Reference (PDR) Network (2021). Prescribers’ digital reference (PDR) Network, LLC. New Jersey, USA: PDR Network.

[B55] Quillardet P. Arrault X. Michel V. Touati E. (2006). Organ-targeted mutagenicity of nitrofurantoin in Big Blue transgenic mice. Mutagenesis 21, 305–311. 10.1093/mutage/gel036 16895946

[B56] Race P. R. Lovering A. L. Green R. M. Ossor A. White S. A. Searle P. F. (2005). Structural and mechanistic studies of *Escherichia coli* nitroreductase with the antibiotic nitrofurazone. Reversed binding orientations in different redox states of the enzyme. J. Biol. Chem. 280, 13256–13264. 10.1074/jbc.M409652200 15684426

[B57] Ré J. L. De Méo M. P. Laget M. Guiraud H. Castegnaro M. Vanelle P. (1997). Evaluation of the genotoxic activity of metronidazole and dimetridazole in human lymphocytes by the comet assay. Mutat. Res. 375 (2), 147–155. 10.1016/s0027-5107(97)00010-9 9202725

[B58] Reitz M. Rumpf M. Knitza R. (1991). DNA single strand-breaks in lymphocytes after metronidazole therapy. Arzneimittelforschung. 41 (2), 155–156. 2043178

[B59] Roldán M. D. Pérez-Reinado E. Castillo F. Moreno-Vivián C. (2008). Reduction of polynitroaromatic compounds: The bacterial nitroreductases. FEMS Microbiol. Rev. 32, 474–500. 10.1111/j.1574-6976.2008.00107.x 18355273

[B60] Rosenkranz H. S. Jr Speck W. T. Stambaugh J. E. (1976). Mutagenicity of metronidazole: Structure-activity relationships. Mutat. Res. 38, 203–206. 10.1016/0165-1161(76)90191-6 778605

[B61] Sardaş S. Metin A. Gök S. Karakaya A. E. Aykol N. (1990). Sister chromatid exchanges in peripheral lymphocytes of urinary tract infection treated with nitrofurantoin. Int. Urol. Nephrol. 22, 513–517. 10.1007/BF02549738 2093691

[B62] Savoldi-Barbosa M. Sakamoto-Hojo E. T. Takahashi C. C. (1999). Influence of novobiocin on g-irradiation G0-lymphocytes as analyzed by cytogenetic endpoints. Genet. Mol. Biol. 22 (2), 217–223. 10.1590/s1415-47571999000200014

[B63] Shirude P. S. Hameed S. (2012). Nonfluoroquinolone-based inhibitors of mycobacterial type II topoisomerase as potential therapeutic agents for TB. Annu. Rep. Med. Chem. 47, 319–330. 10.1016/B978-0-12-396492-2.00021-7

[B64] Slapsyte G. Jankauskiene A. Mierauskiene J. Lazutka J. R. (2002). Cytogenetic analysis of peripheral blood lymphocytes of children treated with nitrofurantoin for recurrent urinary tract infection. Mutagenesis 17, 31–35. 10.1093/mutage/17.1.31 11752231

[B65] Speck W. T. Stein A. B. Rosenkranz H. S. (1976). Mutagenicity of metronidazole: Presence of several active metabolites in human urine. J. Natl. Cancer Inst. 56, 283–284. 10.1093/jnci/56.2.283 1255761

[B66] Stephens M. A. (1986). “Tests based on EDF statistics,” in Goodness-of-Fit techniques. Editors d'Agostino R. B. Stephens M. A. (New York: Marcel Dekker).

[B67] Thompson E. D. (1986). Comparison of *in vivo* and *in vitro* cytogenetic assay results. Environ. Mutagen. 8 (5), 753–767. 10.1002/em.2860080510 3769875

[B68] Tibshirani R. Hastie T. Narasimhan B. Chu G. (2002). Diagnosis of multiple cancer types by shrunken centroids of gene expression. Proc. Natl. Acad. Sci. U. S. A. 99, 6567–6572. 10.1073/pnas.082099299 12011421 PMC124443

[B69] Tice R. R. Agurell E. Anderson D. Burlinson B. Hartmann A. Kobayashi H. (2000). Single cell gel/comet assay: Guidelines for *in vitro* and *in vivo* genetic toxicology testing. Environ. Mol. Mutagen. 35 (3), 206–221. 10.1002/(sici)1098-2280(2000)35:3<206:aid-em8>3.0.co;2-j 10737956

[B70] Turkez H. Arslan M. E. Ozdemir O. (2017). Genotoxicity testing: Progress and prospects for the next decade. Expert Opin. Drug Metab. Toxicol. 13, 1089–1098. 10.1080/17425255.2017.1375097 28889778

[B71] Uhari M. Nuutinen M. Turtinen J. (1996). Adverse reactions in children during long-term antimicrobial therapy. Pediatr. Infect. Dis. J. 15, 404–408. 10.1097/00006454-199605000-00005 8724061

[B72] Venables W. N. Ripley B. D. (2002). Modern applied statistics with S. 4th ed. New York: Springer-Verlag.

[B73] Williams A. Buick J. K. Moffat I. Swartz C. D. Recio L. Hyduke D. R. (2015). A predictive toxicogenomics signature to classify genotoxic versus non-genotoxic chemicals in human TK6 cells. Data Brief. 5, 77–83. 10.1016/j.dib.2015.08.013 26425668 PMC4564388

[B74] Wood D. K. Weingeist D. M. Bhatia S. N. Engelward B. P. (2010). Single cell trapping and DNA damage analysis using microwell arrays. Proc. Natl. Acad. Sci. U. S. A. 107, 10008–10013. 10.1073/pnas.1004056107 20534572 PMC2890454

[B75] World Health Organization (2019). World health organization model list of essential medicines: 21st list 2019.

[B76] Yauk C. L. Buick J. K. Williams A. Swartz C. D. Recio L. Li H. H. (2016). Application of the TGx-28.65 transcriptomic biomarker to classify genotoxic and non-genotoxic chemicals in human TK6 cells in the presence of rat liver S9. Environ. Mol. Mutagen. 57, 243–260. 10.1002/em.22004 26946220 PMC5021161

