# Supplementary material for: Integrated Genotoxicity Testing of three anti-infective drugs using the TGx-DDI transcriptomic biomarker and high-throughput CometChip® assay in TK6 cells
Source: Front Toxicol. 2022 Sep 23;4:991590. doi: 10.3389/ftox.2022.991590 (PMC9540394; doi:10.3389/ftox.2022.991590)
Supplement: Supplementary file 1 [file DataSheet1.PDF]

Supplementary Figure 1A: Nitrofurantoin (NIT)

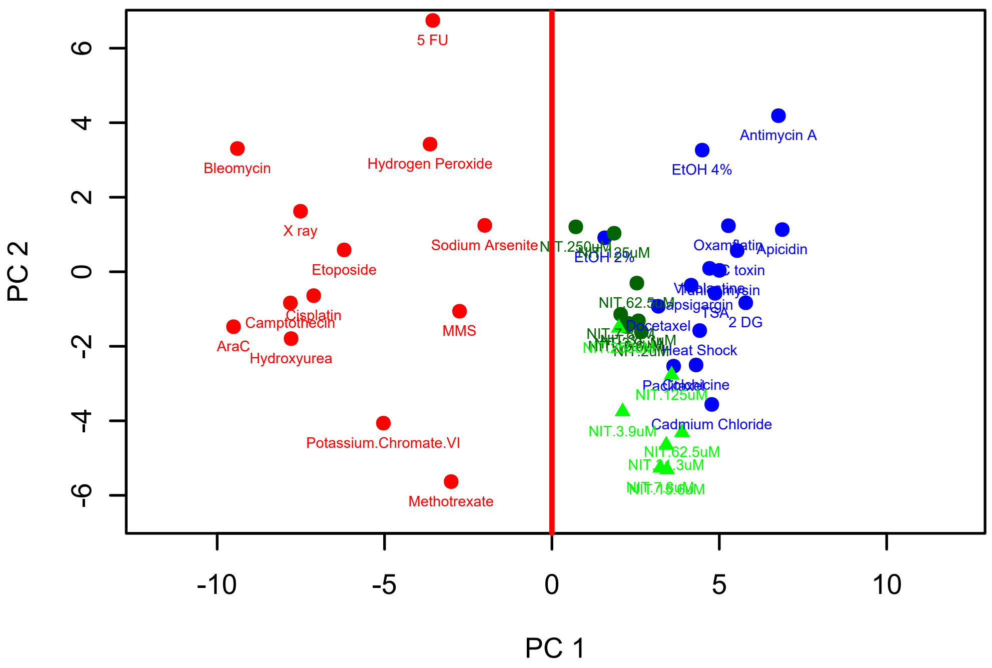

Supplementary Figure 1B: Metronidazole (MTZ)

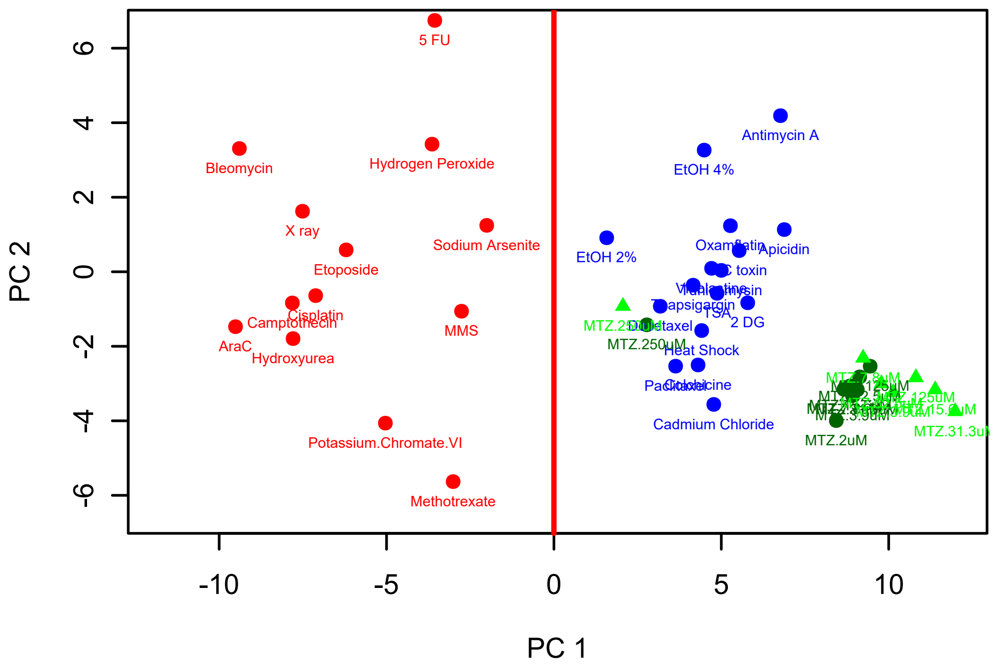

Supplementary Figure 1C: Novobiocin (NOV)

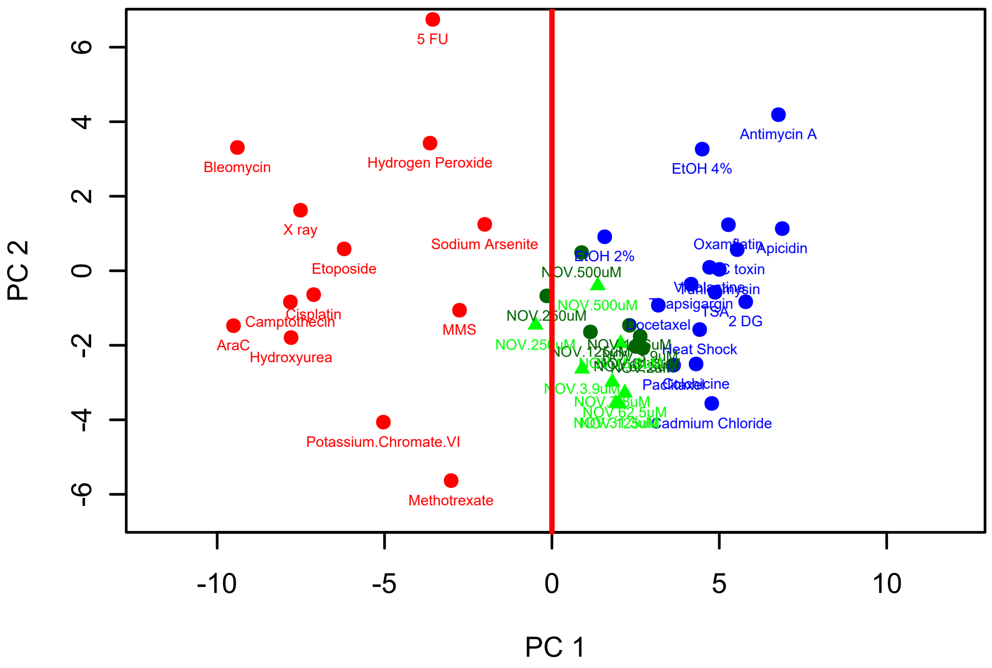

Supplementary Figure 1D: Controls (-ve CA; +ve BL, IR)

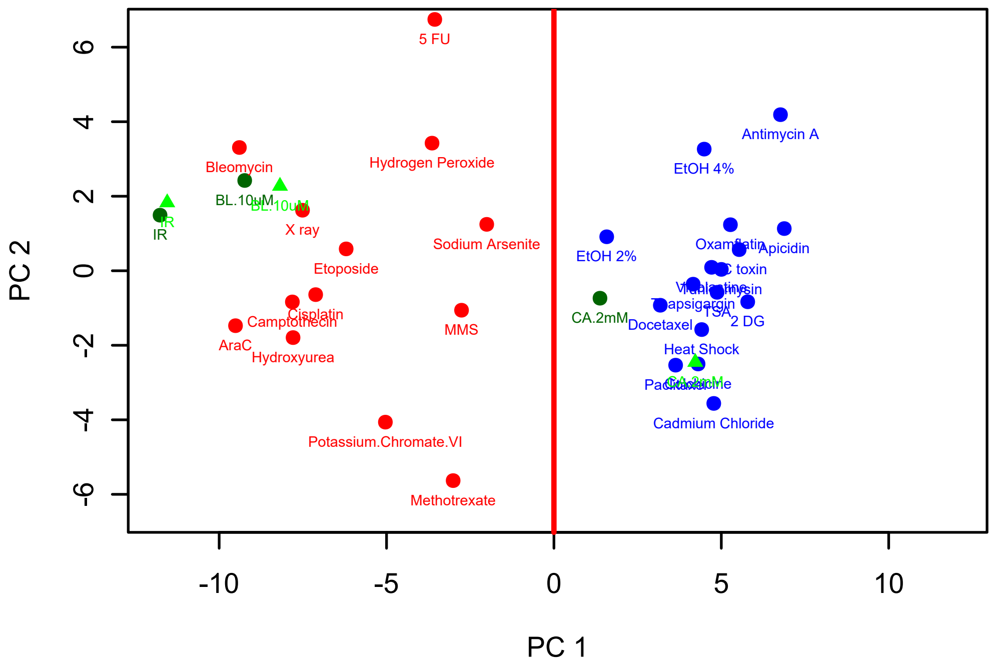

Supplementary Figure 1A-D: Principal component analysis (PCA) depicting TGx-DDI classification results for three anti-infective drugs shown in panels: (1A) nitrofurantoin (NIT), (1B) metronidazole (MTZ), and (1C) novobicin (NOV). The negative control (2 mM caffeine (CA)) and positive controls, including 10 µM bleomycin (BL) and 4 Gy ionizing radiation (IR) are shown in panel (1D).

PCA using the TGx-DDI biomarker for TK6 cells exposed to the training set of chemicals (red font = DDI training set; blue font = non-DDI training set) and to three test chemicals at increasing concentrations (2 µM - 250 µM for NIT and MTZ and 2 µM - 500 µM for NOV) 4 hr following exposure (green font = replicates of test agent; dark green font = NanoString nCounter® data and neon green font = BioSpyder TempO-Seq® data). The line drawn at 0 on the PCA plot divides the DDI and non-DDI agents and was used for classification.

Euclidean, Average Linkage (log2)

Supplementary Figure 2A(i): 2 mM CA (NanoString nCounter®)

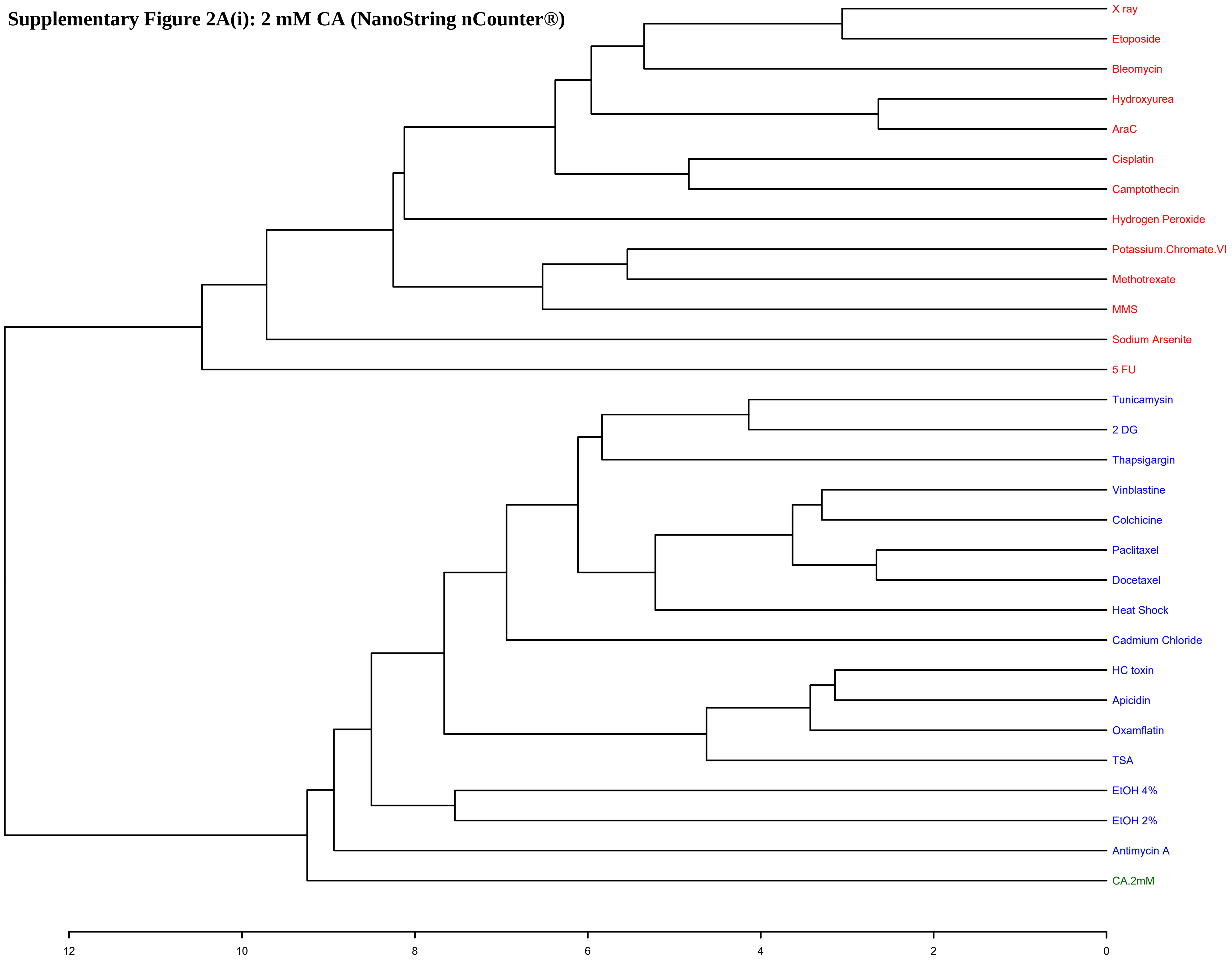

Euclidean, Average Linkage (log2)

Supplementary Figure 2A(ii): 2 mM CA (BioSpyder TempO-Seq®)

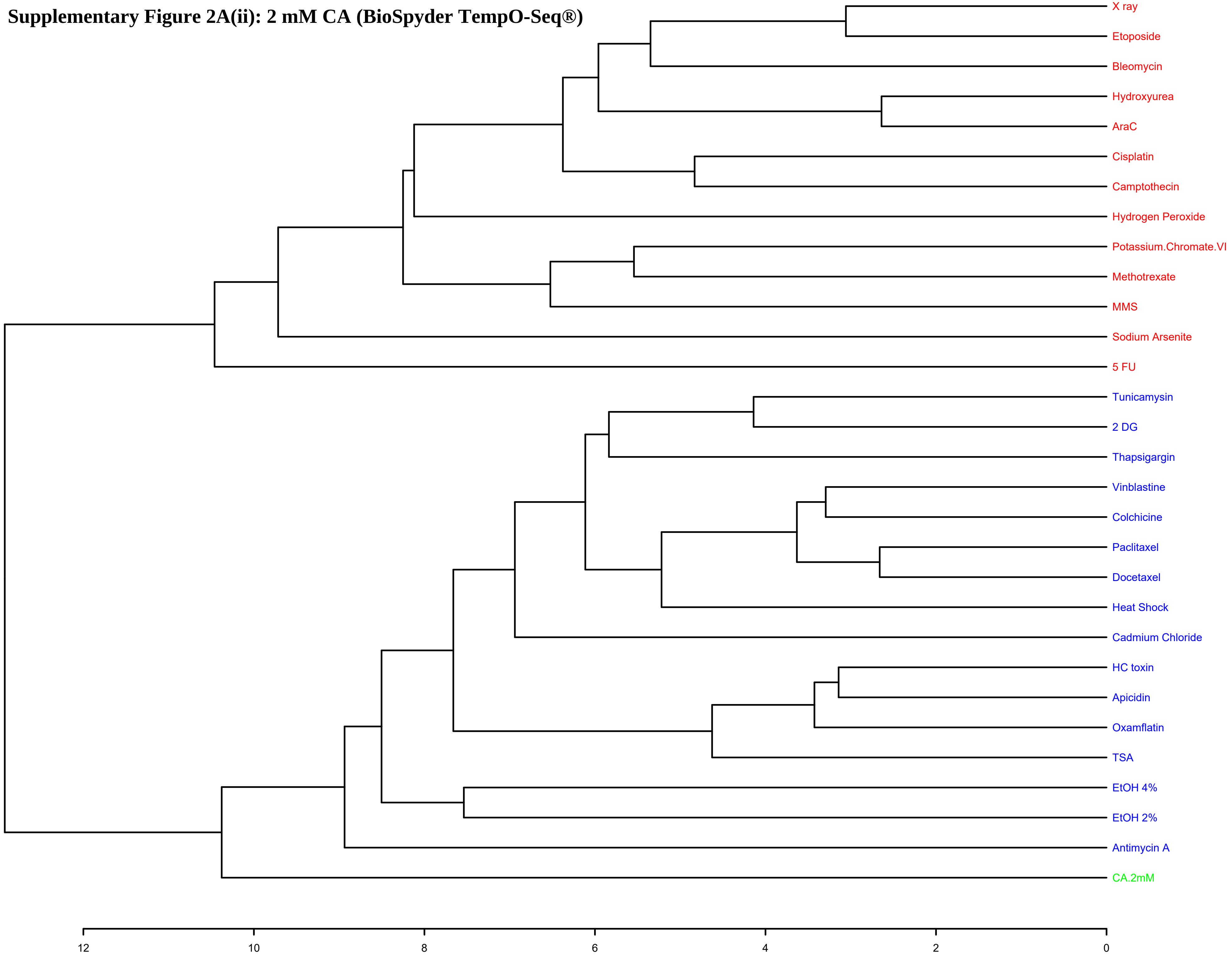

Euclidean, Average Linkage (log2)

Supplementary Figure 2B(i): 10 μM BL (NanoString nCounter®)

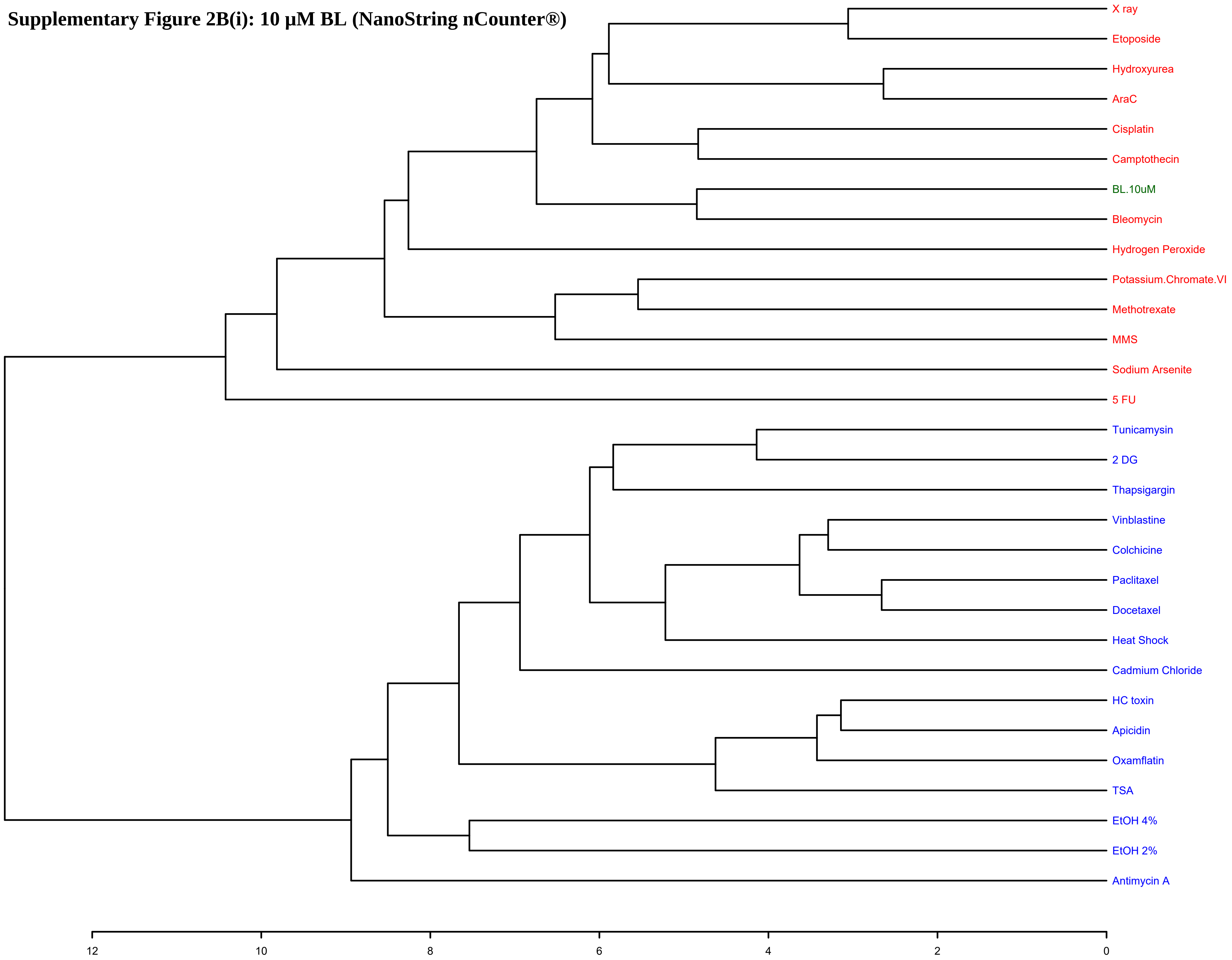

Euclidean, Average Linkage (log2)

Supplementary Figure 2B(ii): 10 µM BL (BioSpyder TempO-Seq®)

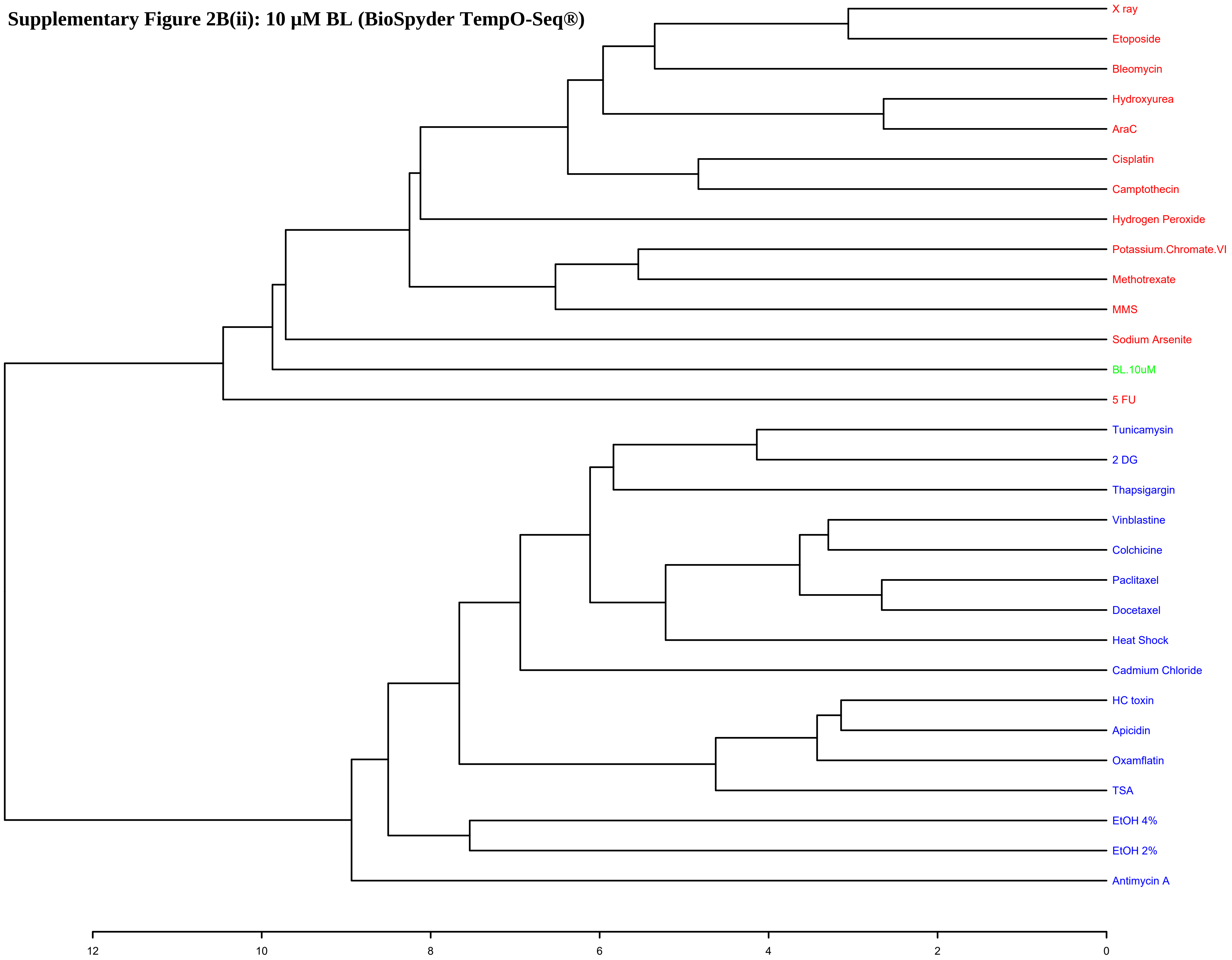

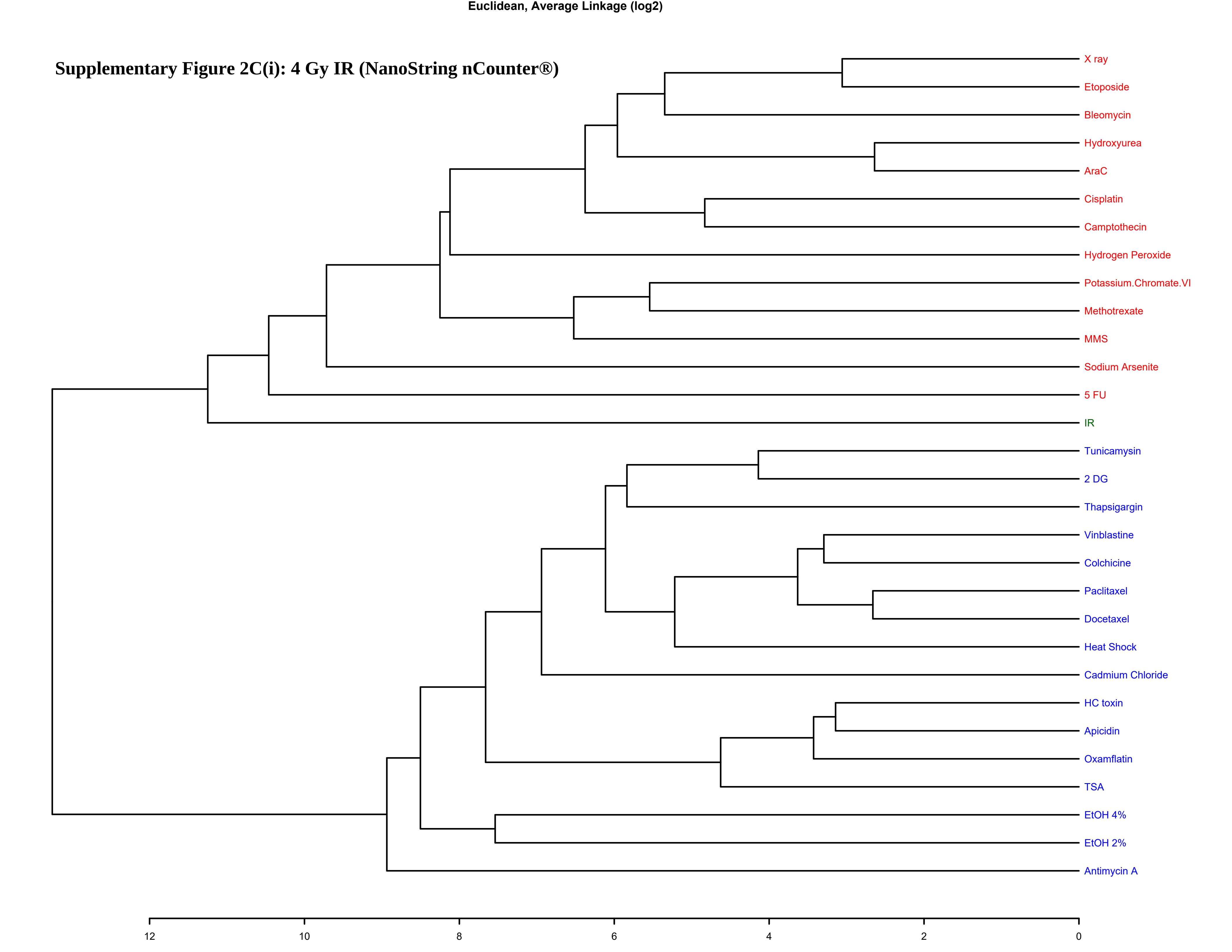
Euclidean, Average Linkage (log2)
Supplementary Figure 2C(i): 4 Gy IR (NanoString nCounter®)
X ray
Etoposide
Bleomycin
Hydroxyurea
AraC
Cisplatin
Camptothecin
Hydrogen Peroxide
Potassium.Chromate.VI
Methotrexate
MMS
Sodium Arsenite
5 FU
IR
Tunicamysin
2 DG
Thapsigargin
Vinblastine
Colchicine
Paclitaxel
Docetaxel
Heat Shock
Cadmium Chloride
HC toxin
Apicidin
Oxamflatin
TSA
EtOH 4%
EtOH 2%
Antimycin A
12
10
8
6
4
2
0

Euclidean, Average Linkage (log2)

Supplementary Figure 2C(ii): 4 Gy IR (BioSpyder TempO-Seq®)

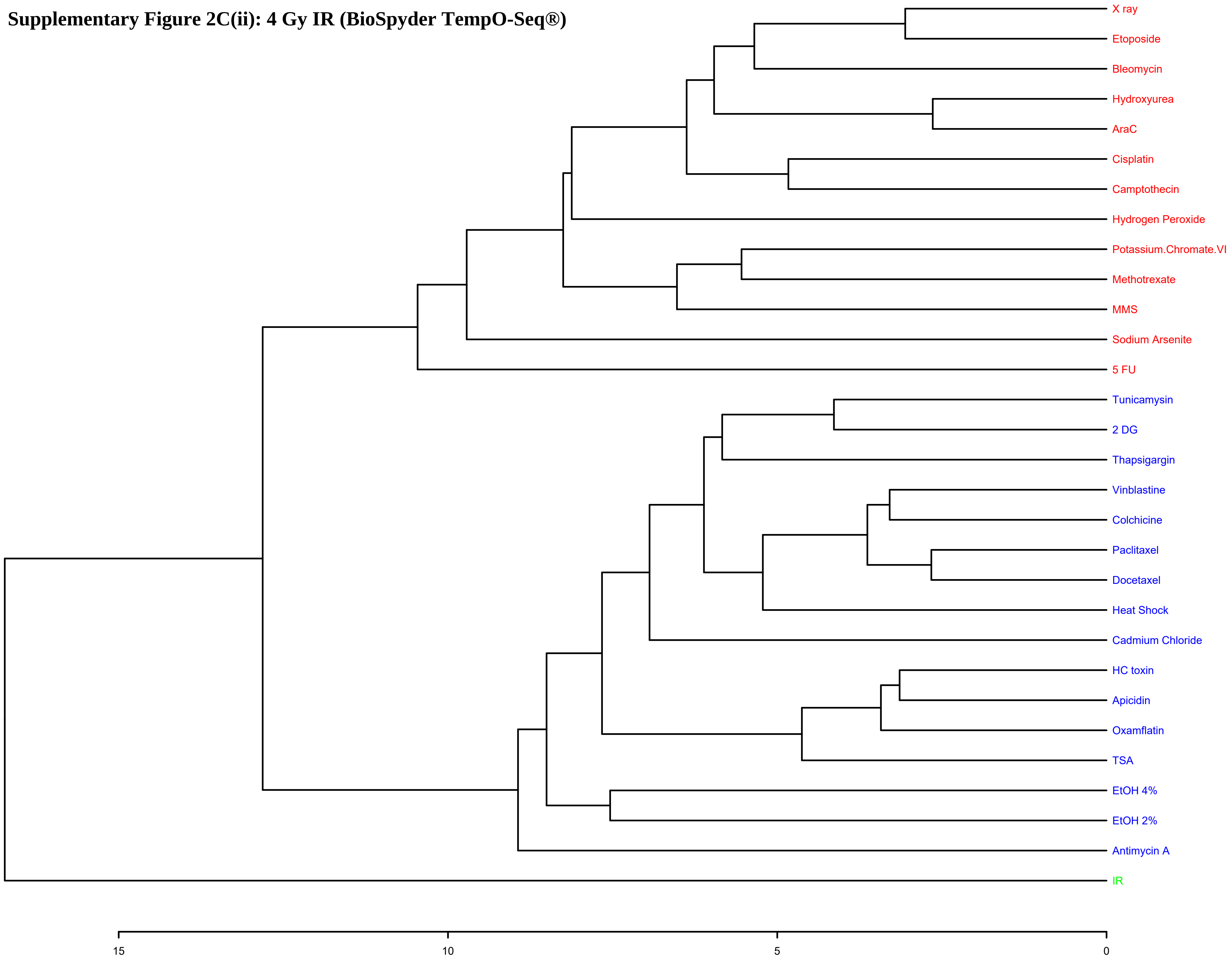

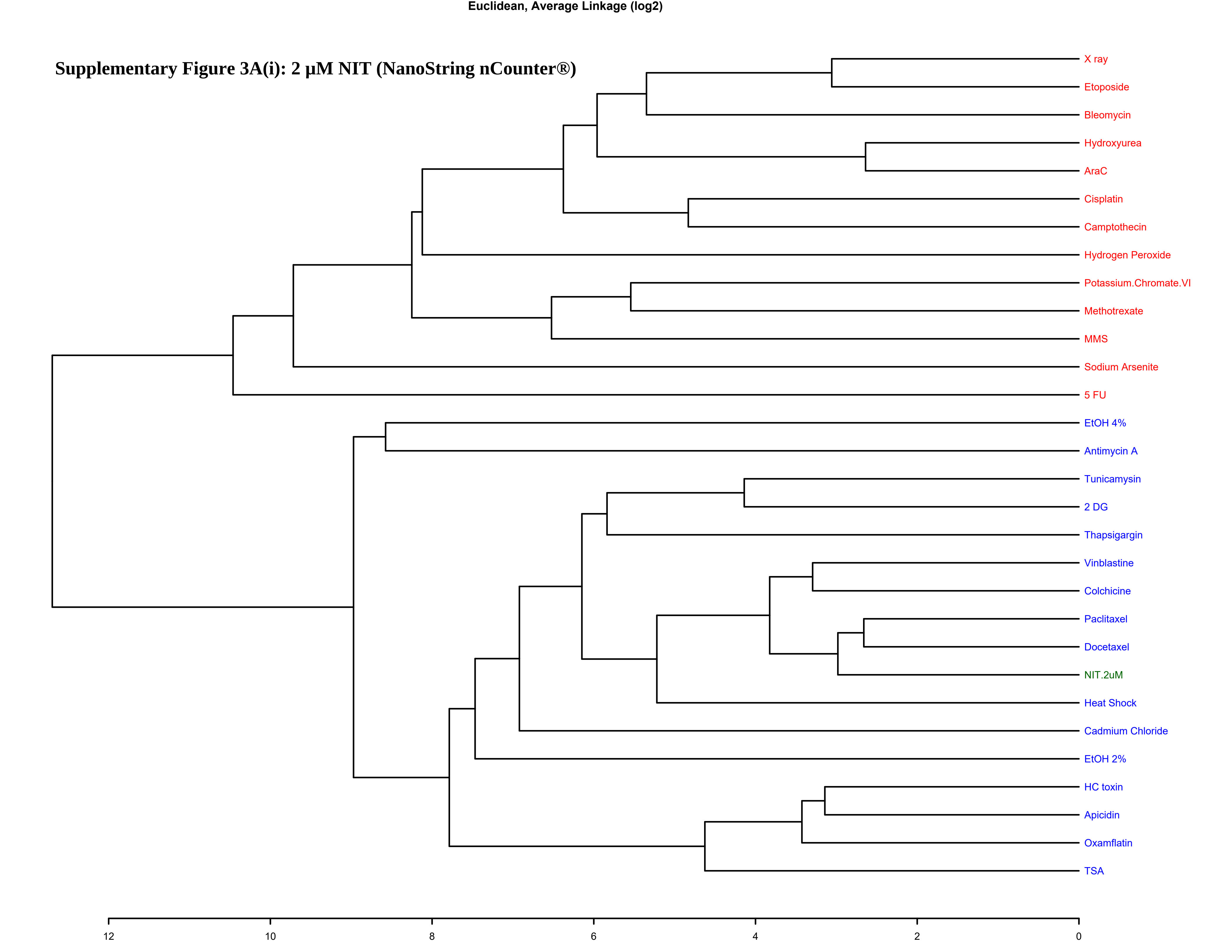
Euclidean, Average Linkage (log2)
Supplementary Figure 3A(i): 2 µM NIT (NanoString nCounter®)
X ray
Etoposide
Bleomycin
Hydroxyurea
AraC
Cisplatin
Camptothecin
Hydrogen Peroxide
Potassium.Chromate.VI
Methotrexate
MMS
Sodium Arsenite
5 FU
EtOH 4%
Antimycin A
Tunicamysin
2 DG
Thapsigargin
Vinblastine
Colchicine
Paclitaxel
Docetaxel
NIT.2uM
Heat Shock
Cadmium Chloride
EtOH 2%
HC toxin
Apicidin
Oxamflatin
TSA
12
10
8
6
4
2
0

Supplementary Figure 3B (i): 3.9 µM NIT (NanoString nCounter®)

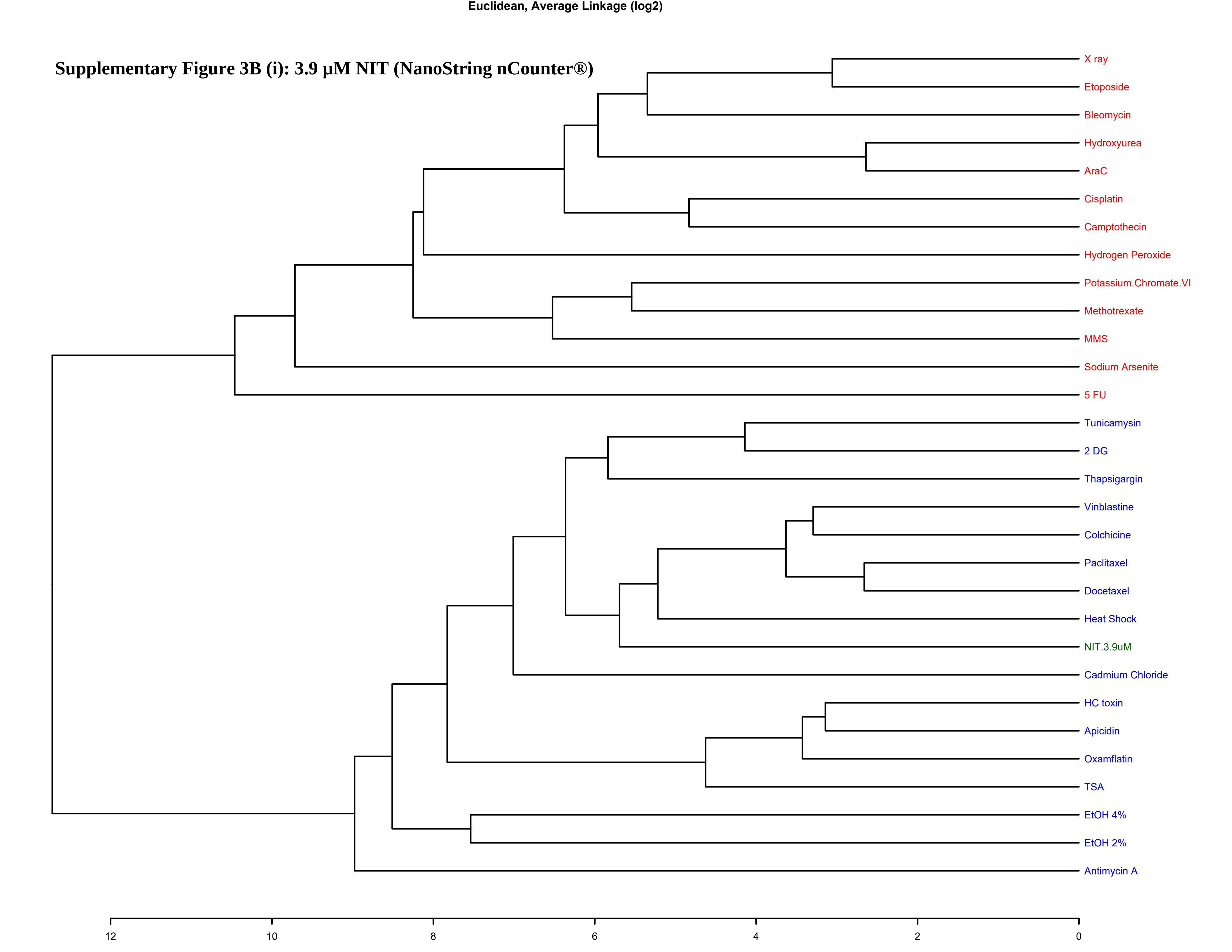

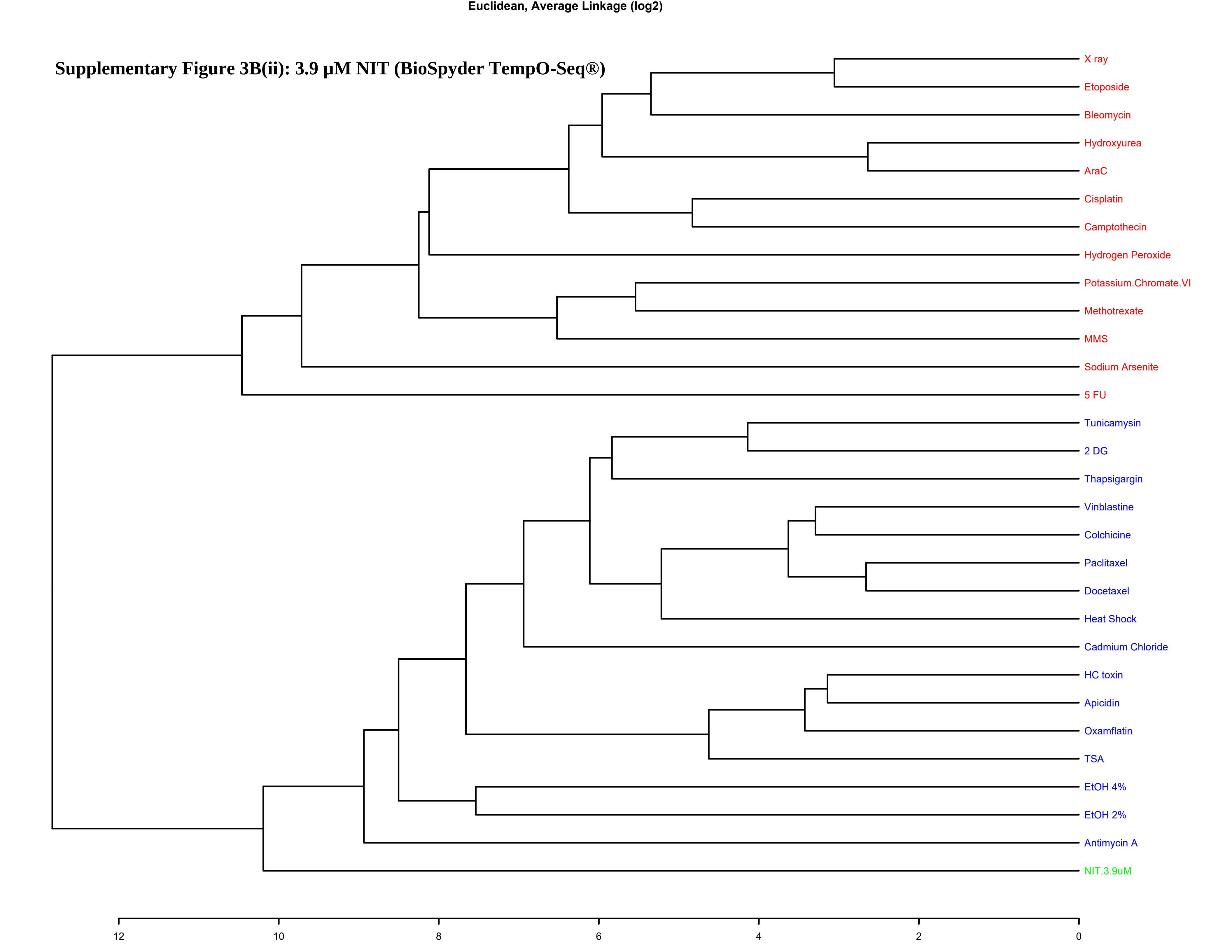
Euclidean, Average Linkage (log2)
Supplementary Figure 3B(ii): 3.9 µM NIT (BioSpyder TempO-Seq®)
X ray
Etoposide
Bleomycin
Hydroxyurea
AraC
Cisplatin
Camptothecin
Hydrogen Peroxide
Potassium.Chromate.VI
Methotrexate
MMS
Sodium Arsenite
5 FU
Tunicamysin
2 DG
Thapsigargin
Vinblastine
Colchicine
Paclitaxel
Docetaxel
Heat Shock
Cadmium Chloride
HC toxin
Apicidin
Oxamflatin
TSA
EtOH 4%
EtOH 2%
Antimycin A
NIT.3.9uM
12
10
8
6
4
2
0

Euclidean, Average Linkage (log2)

Supplementary Figure 3C(i): 7.8 μM NIT (NanoString nCounter®)

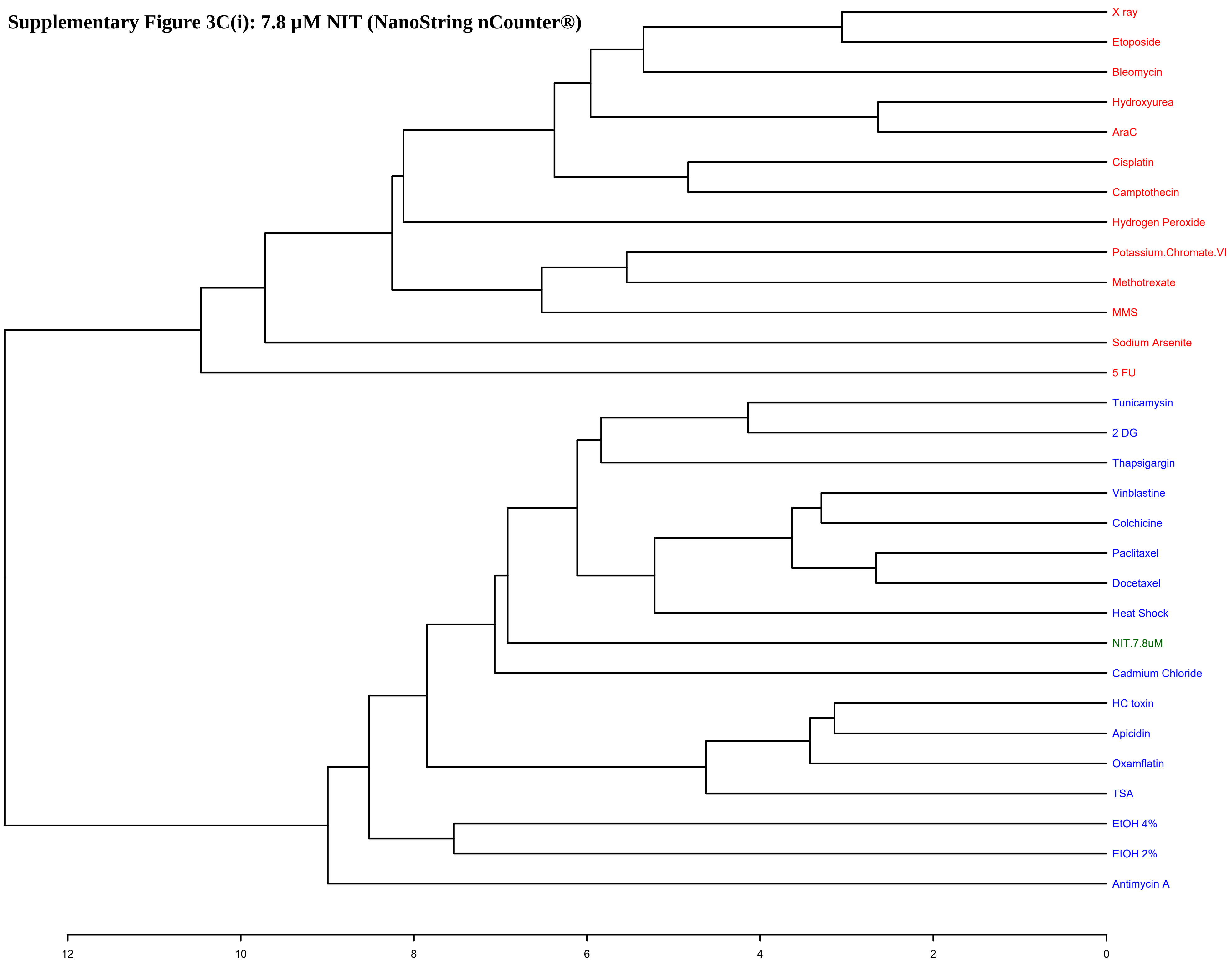

Euclidean, Average Linkage (log2)

Supplementary Figure 3C(ii): 7.8 μM NIT (BioSpyder TempO-Seq®)

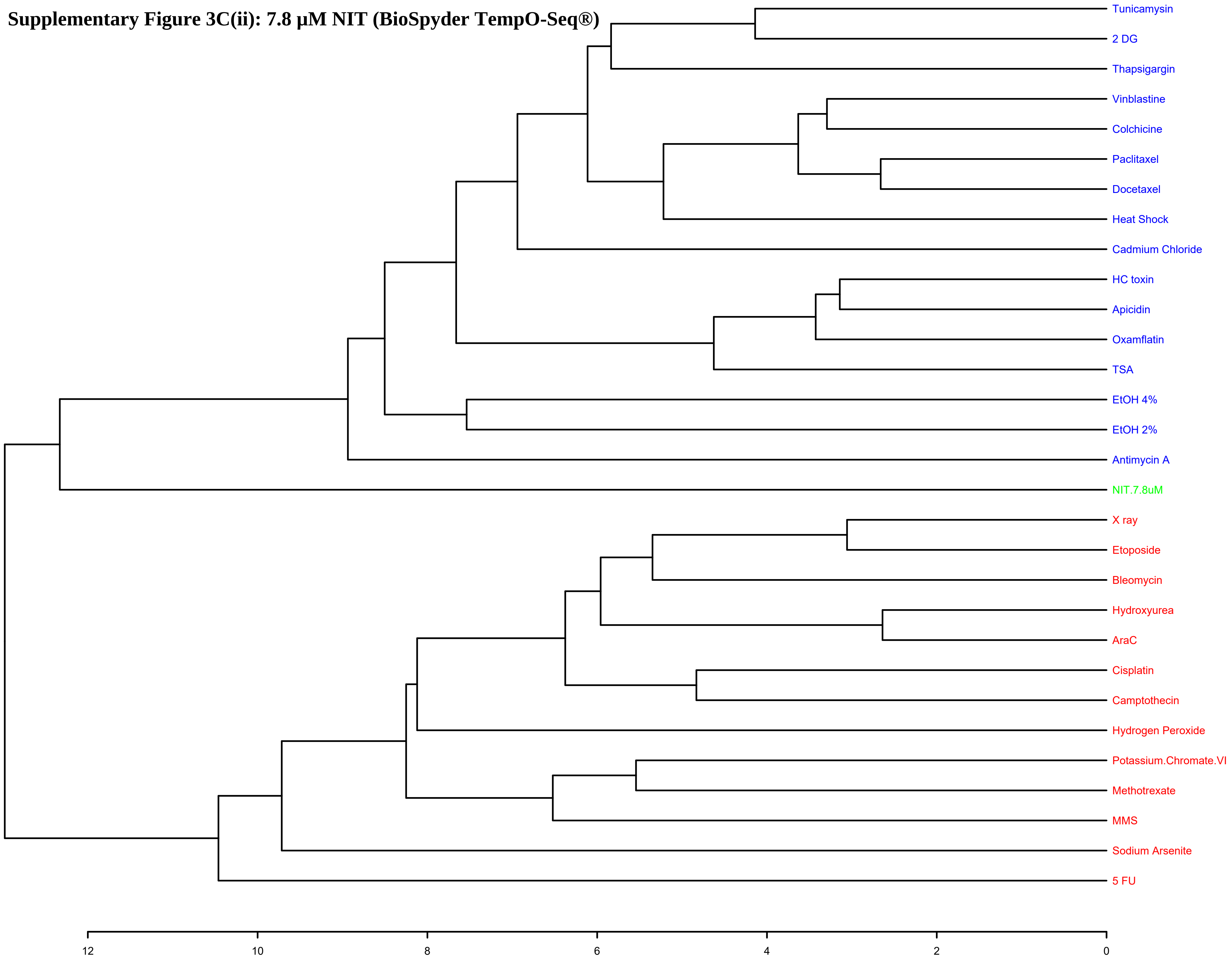

Euclidean, Average Linkage (log2)

Supplementary Figure 3D(i): 15.6 µM NIT (NanoString nCounter®)

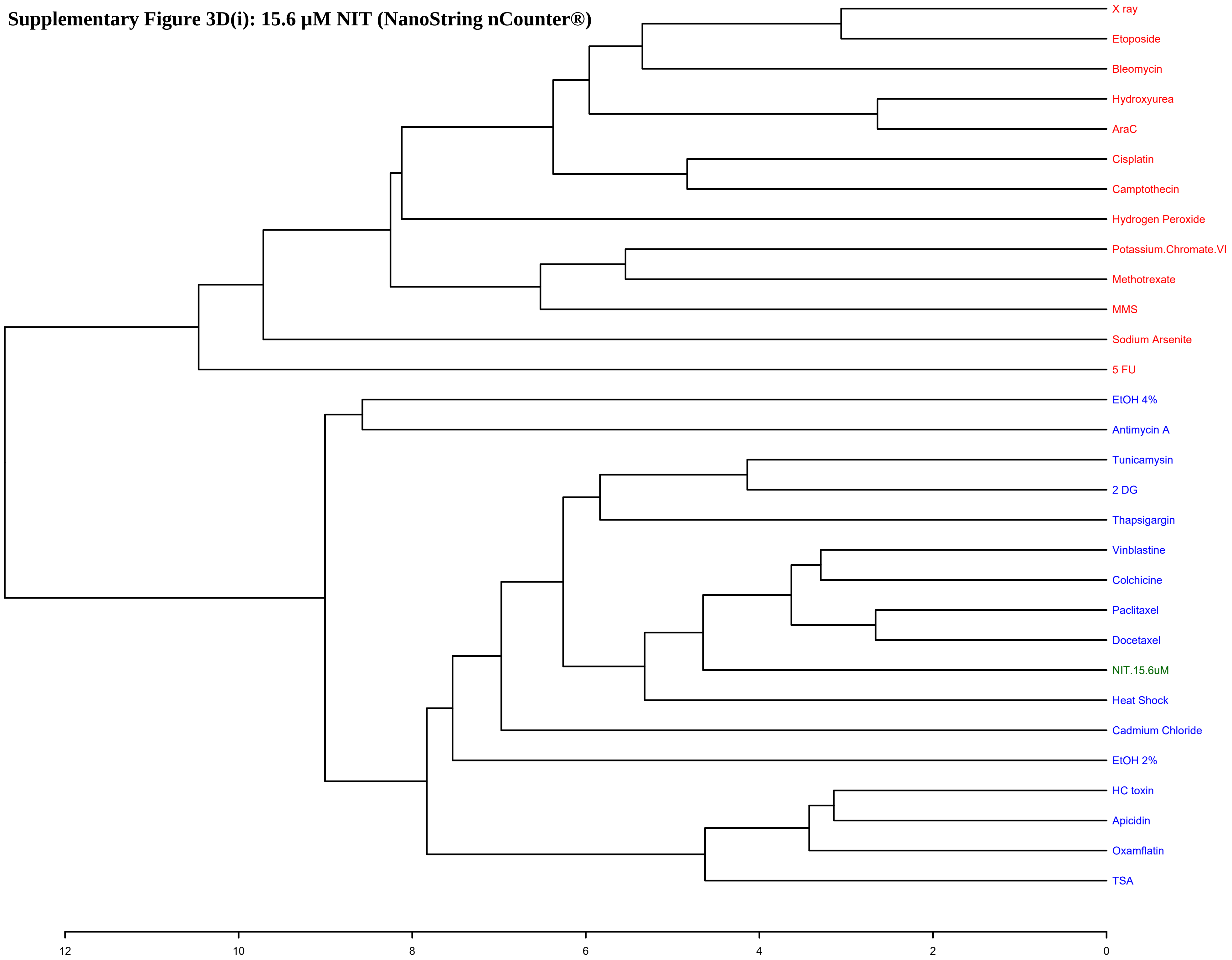

Euclidean, Average Linkage (log2)

Supplementary Figure 3D(ii): 15.6 μM NIT (BioSpyder TempO-Seq®)

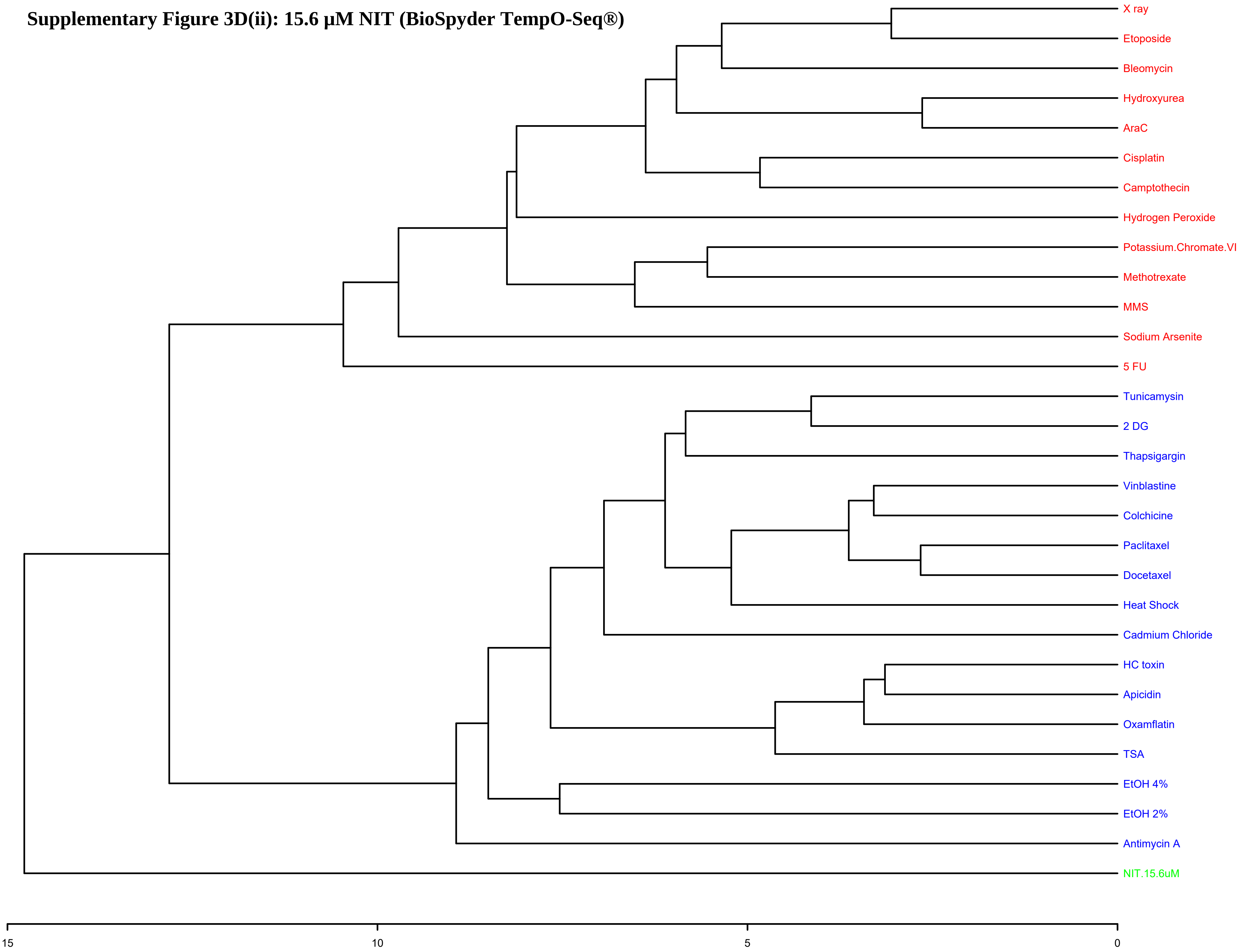

Euclidean, Average Linkage (log2)

Supplementary Figure 3E(i): 31.3 μM NIT (NanoString nCounter®)

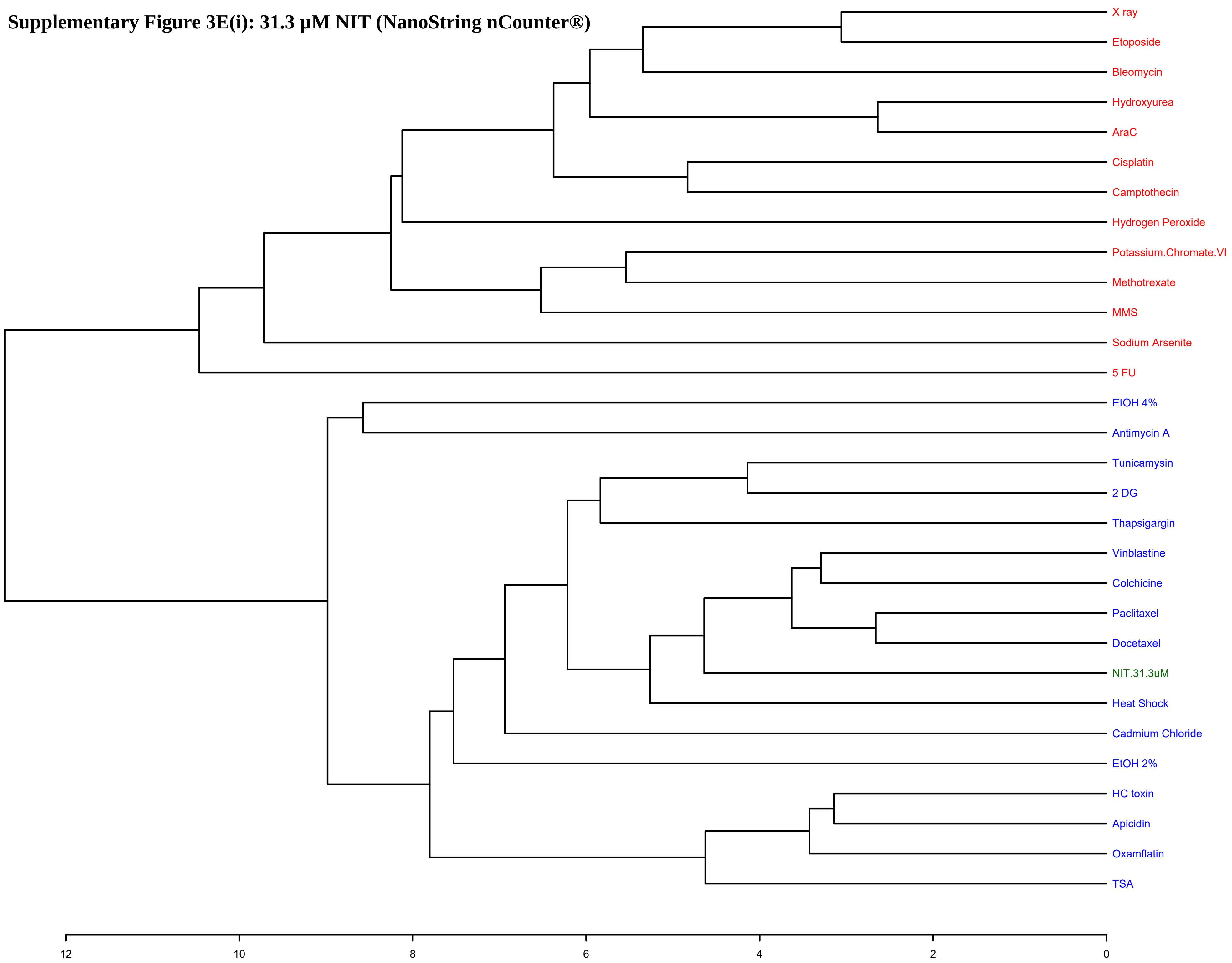

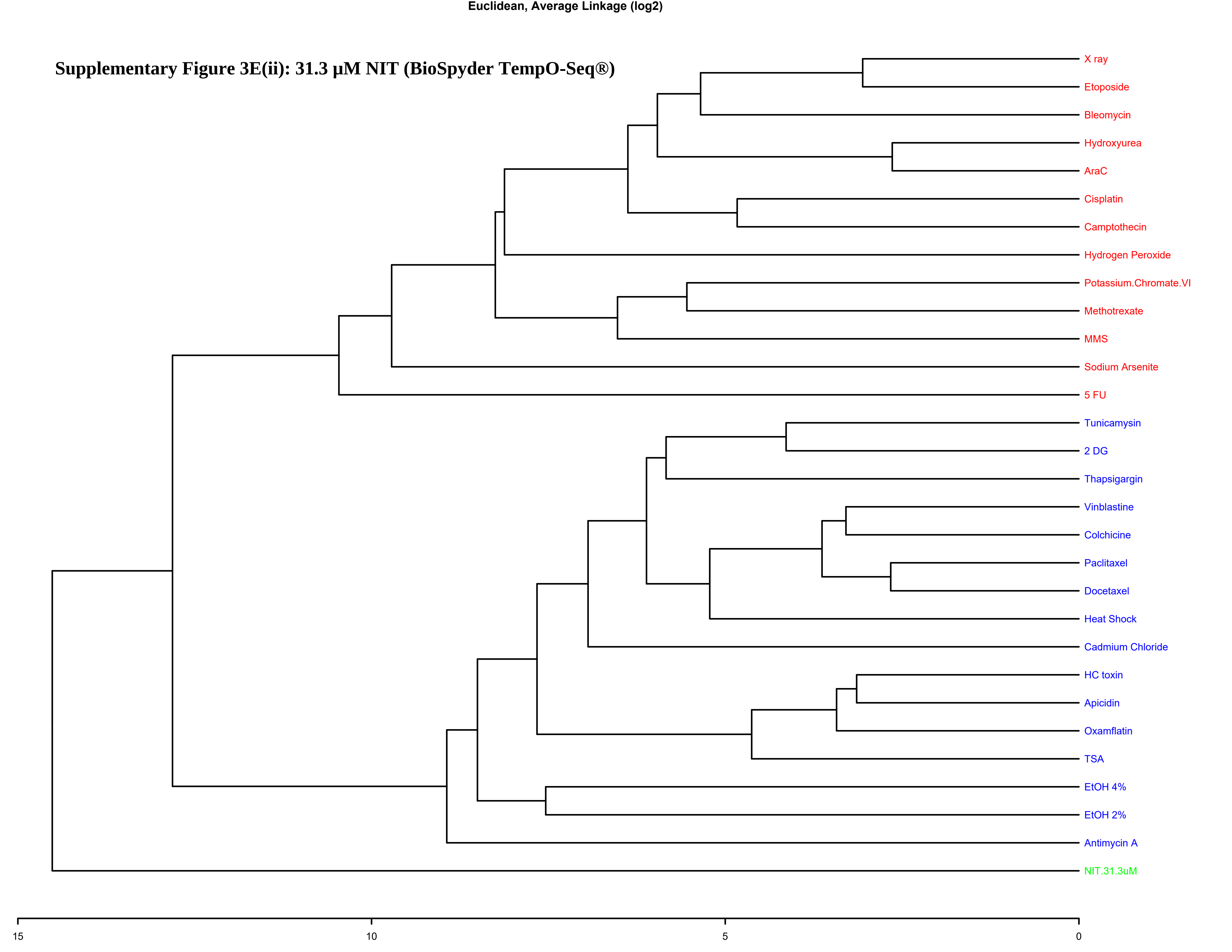
Euclidean, Average Linkage (log2)
Supplementary Figure 3E(ii): 31.3 µM NIT (BioSpyder TempO-Seq®)
X ray
Etoposide
Bleomycin
Hydroxyurea
AraC
Cisplatin
Camptothecin
Hydrogen Peroxide
Potassium.Chromate.VI
Methotrexate
MMS
Sodium Arsenite
5 FU
Tunicamysin
2 DG
Thapsigargin
Vinblastine
Colchicine
Paclitaxel
Docetaxel
Heat Shock
Cadmium Chloride
HC toxin
Apicidin
Oxamflatin
TSA
EtOH 4%
EtOH 2%
Antimycin A
NIT.31.3uM
15
10
5
0

Supplementary Figure 3F(i): 62.5 µM NIT (NanoString nCounter®)

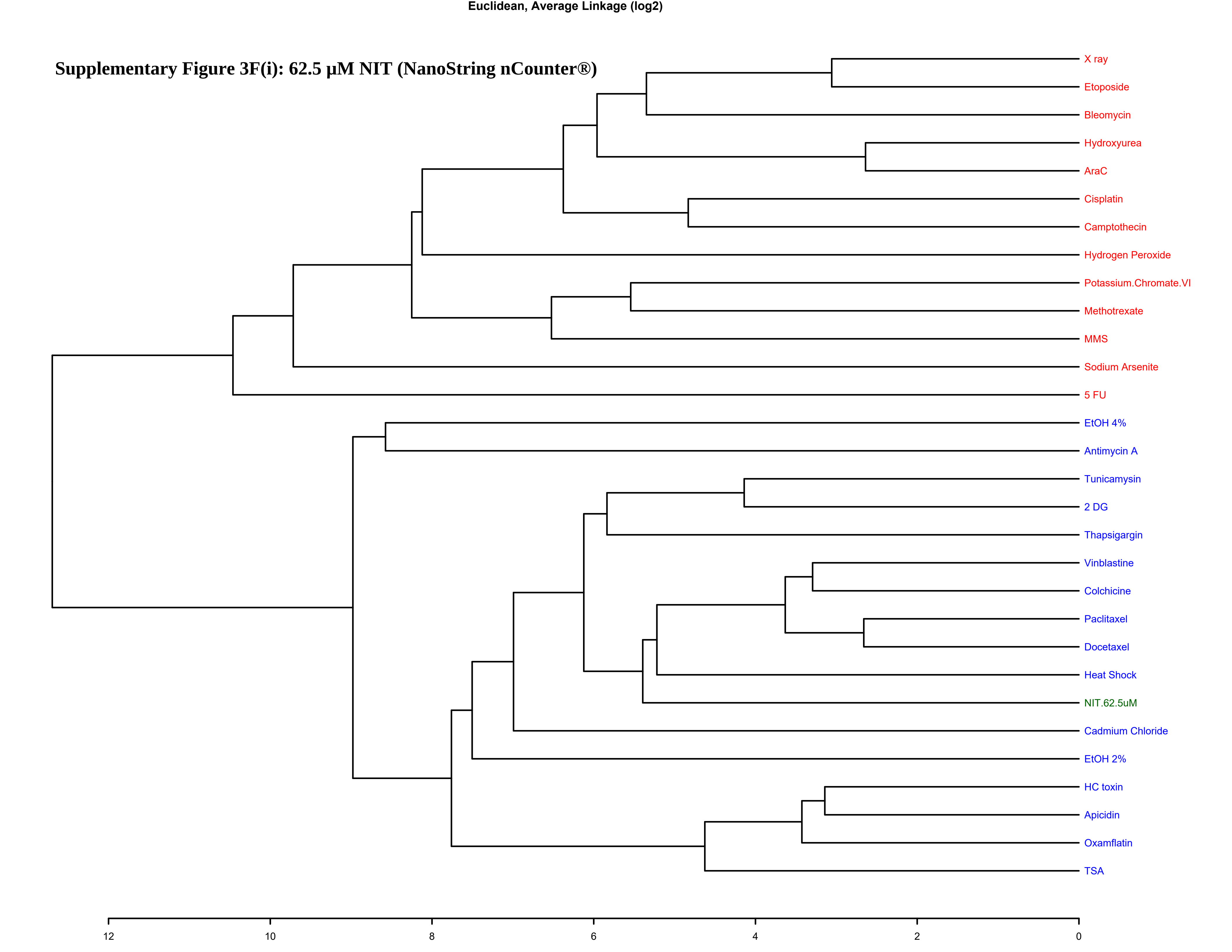

Euclidean, Average Linkage (log2)

Supplementary Figure 3F(ii): 62.5 µM NIT (BioSpyder TempO-Seq®)

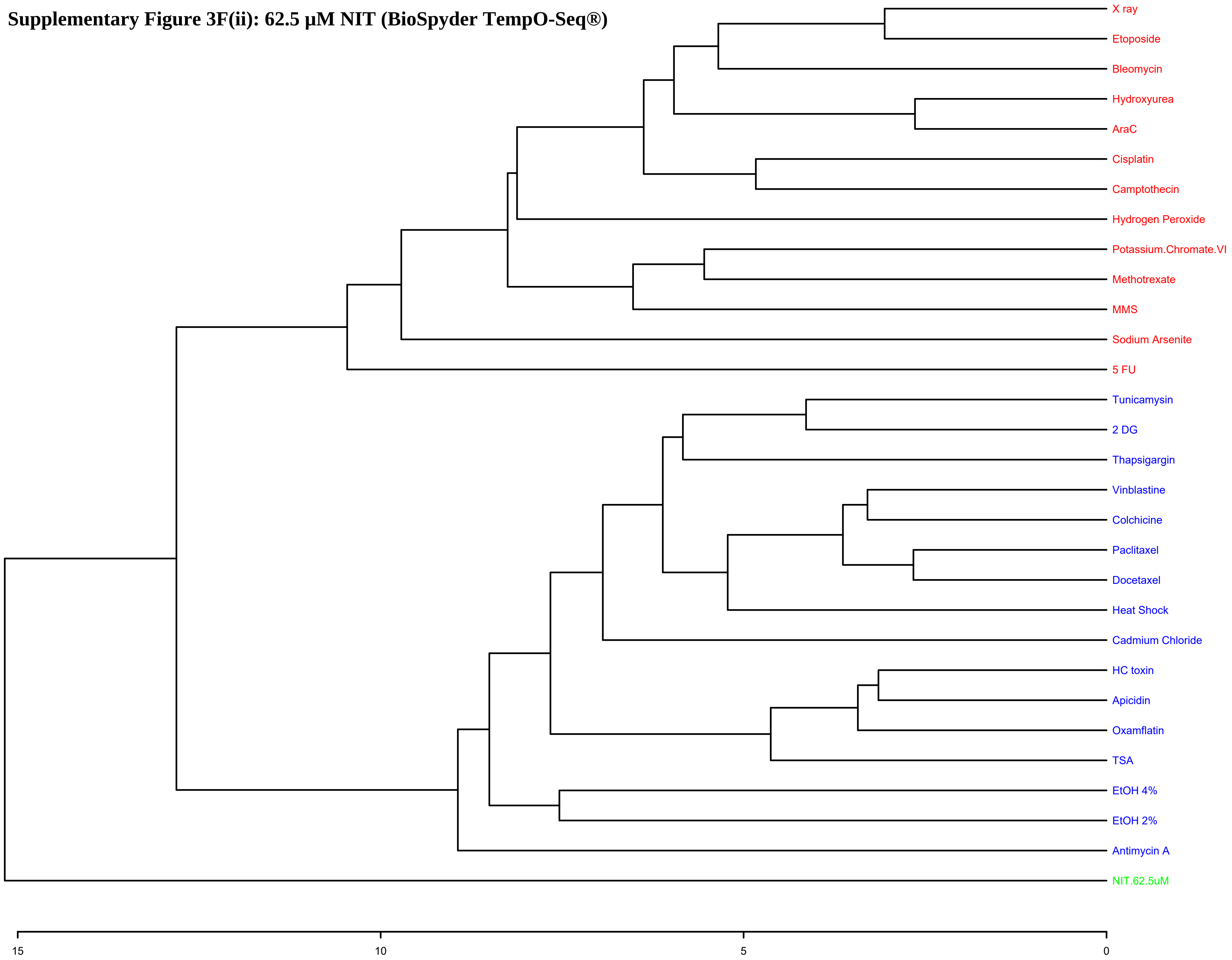

Euclidean, Average Linkage (log2)

Supplementary Figure 3G(i): 125 µM NIT (NanoString nCounter®)

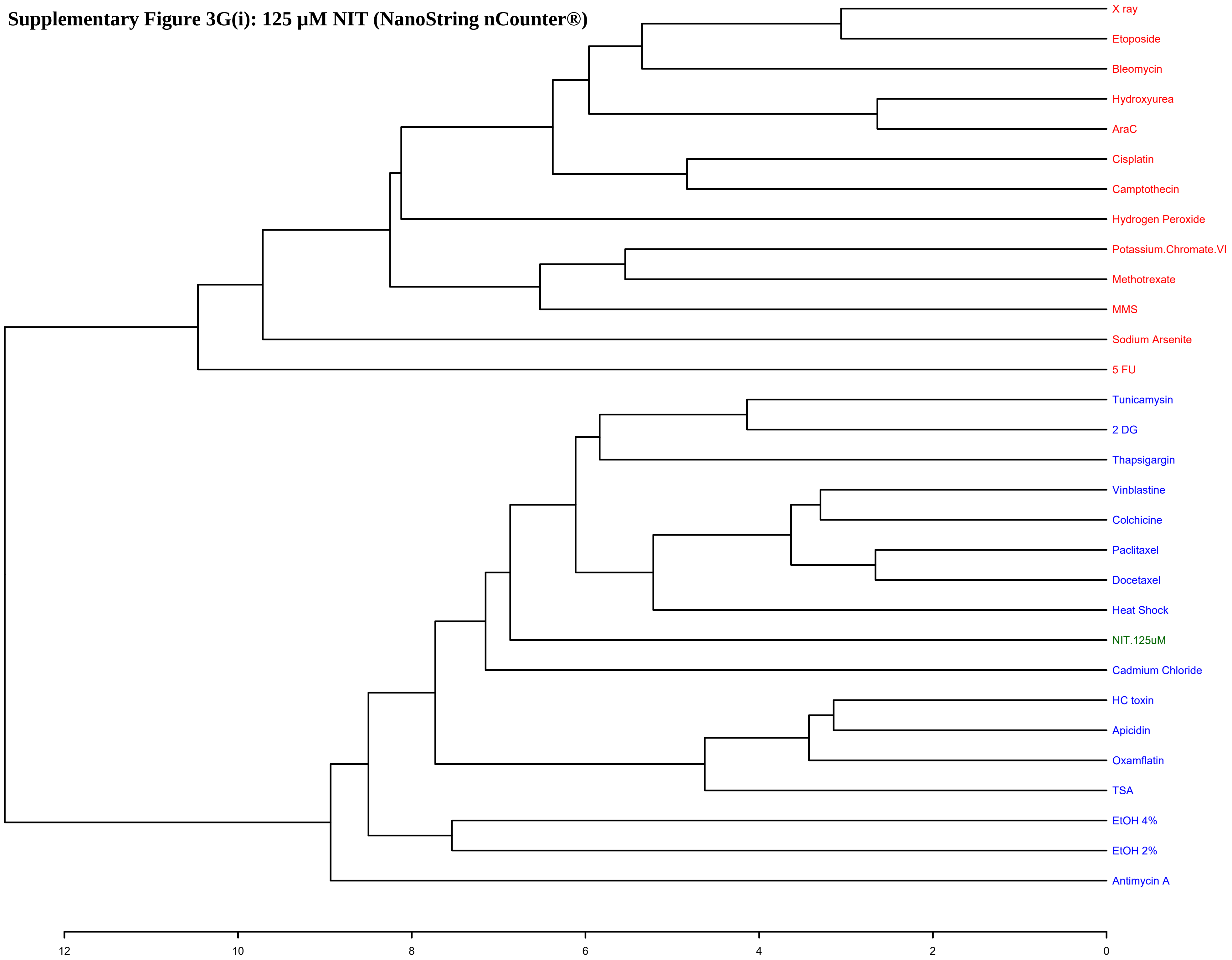

Supplementary Figure 3G(ii): 125 µM NIT (BioSpyder TempO-Seq®)

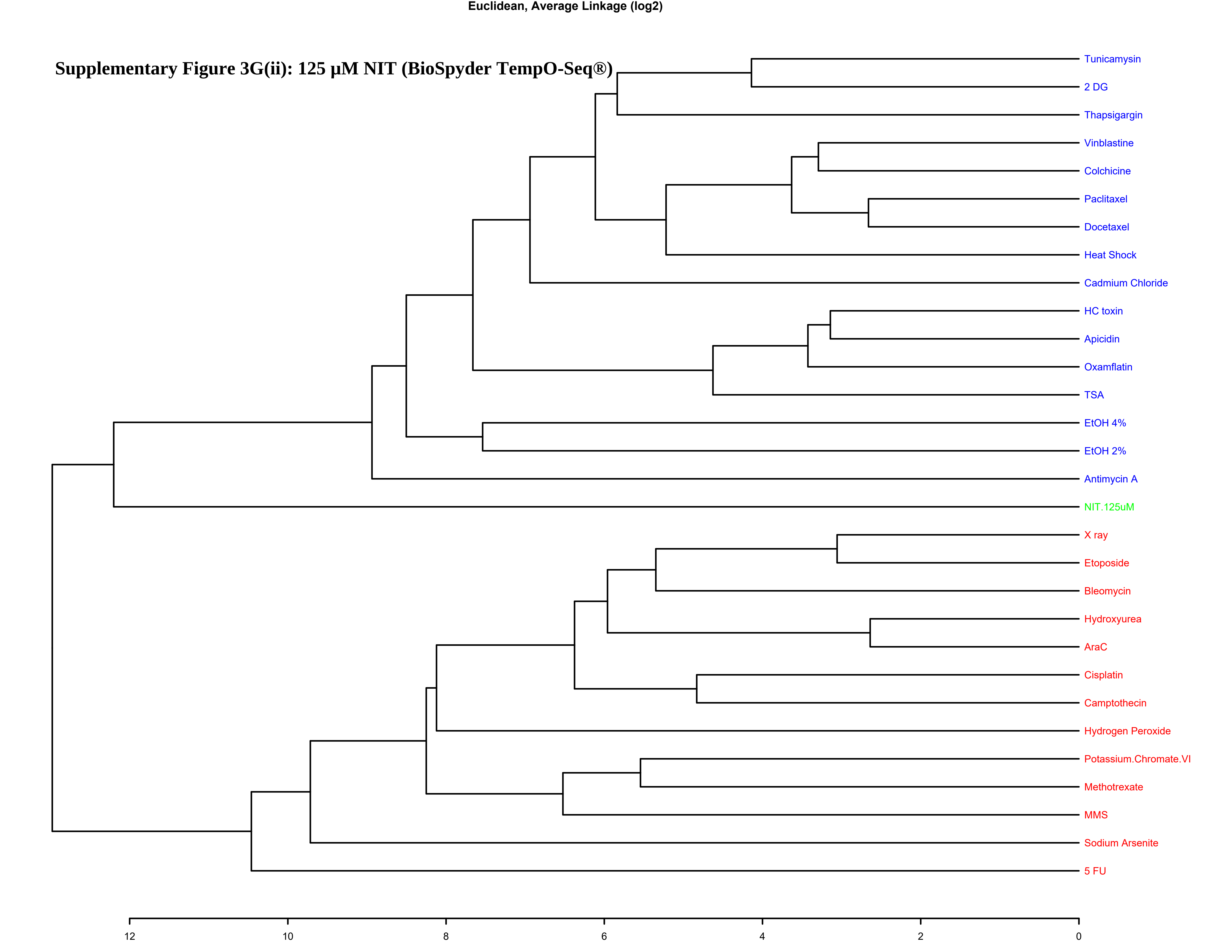

Euclidean, Average Linkage (log2)

Supplementary Figure 3H(i): 250 µM NIT (NanoString nCounter®)

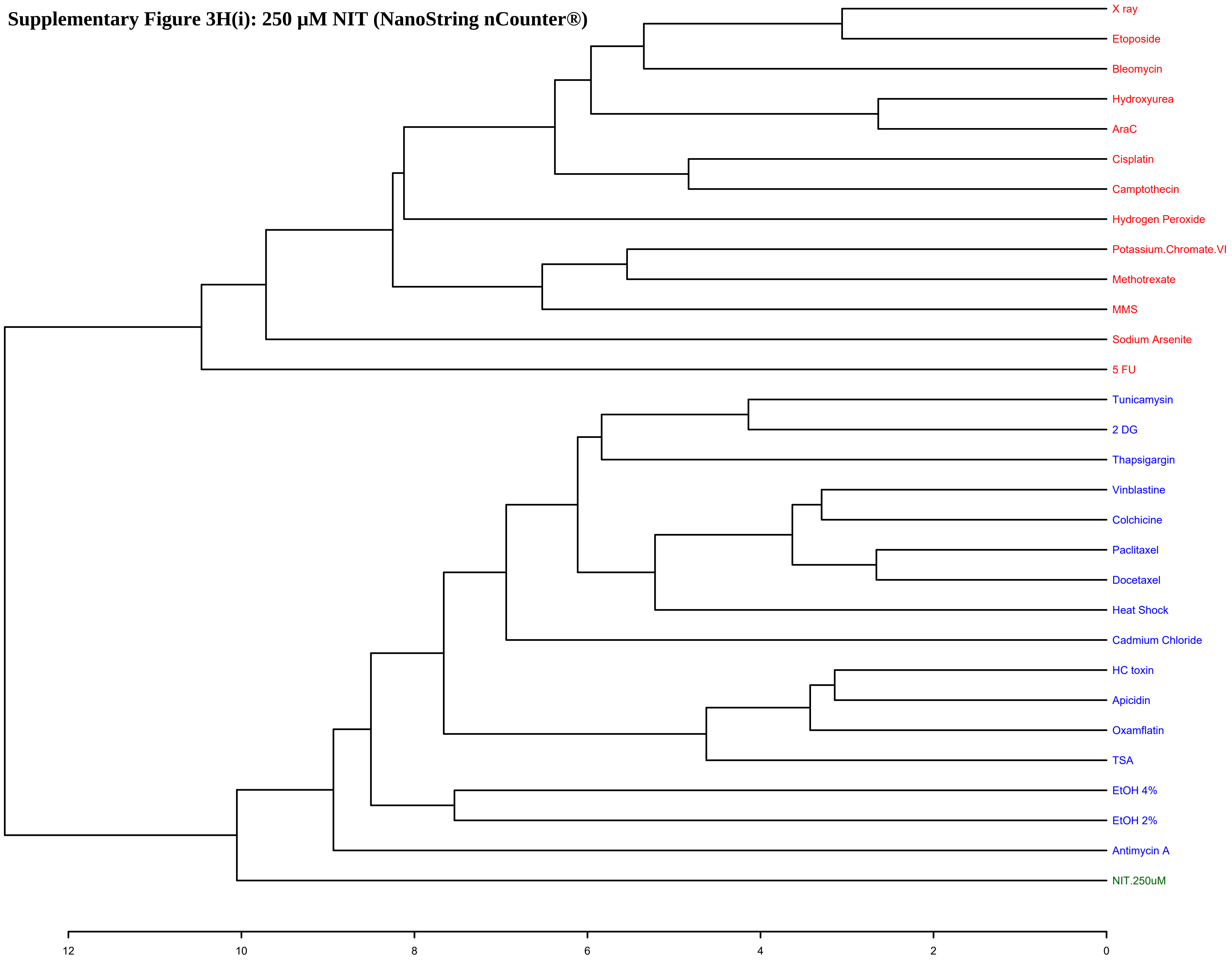

Supplementary Figure 3H(ii): 250 μM NIT (BioSpyder TempO-Seq®)

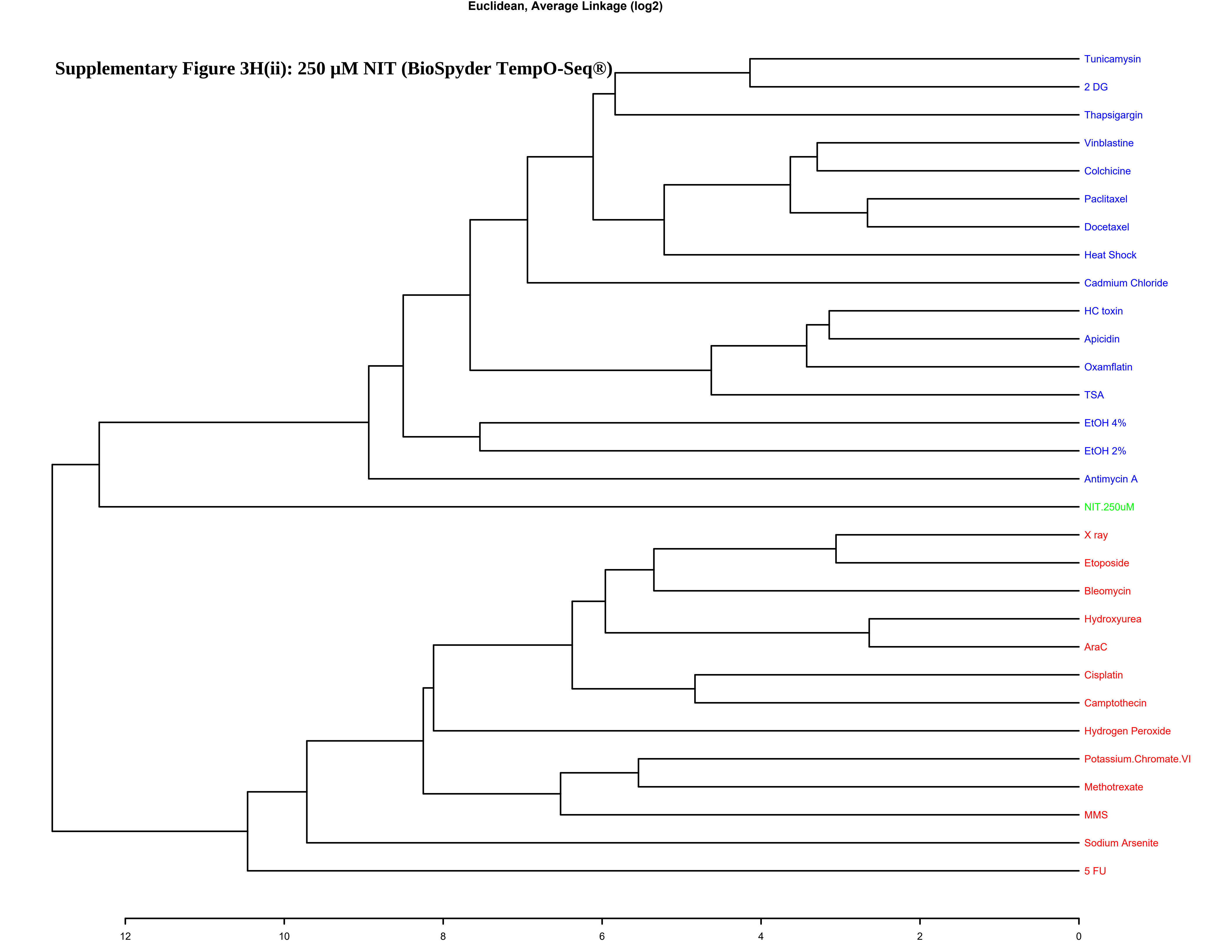

Euclidean, Average Linkage (log2)

Supplementary Figure 4A(i): 2 μM MTZ (NanoString nCounter®)

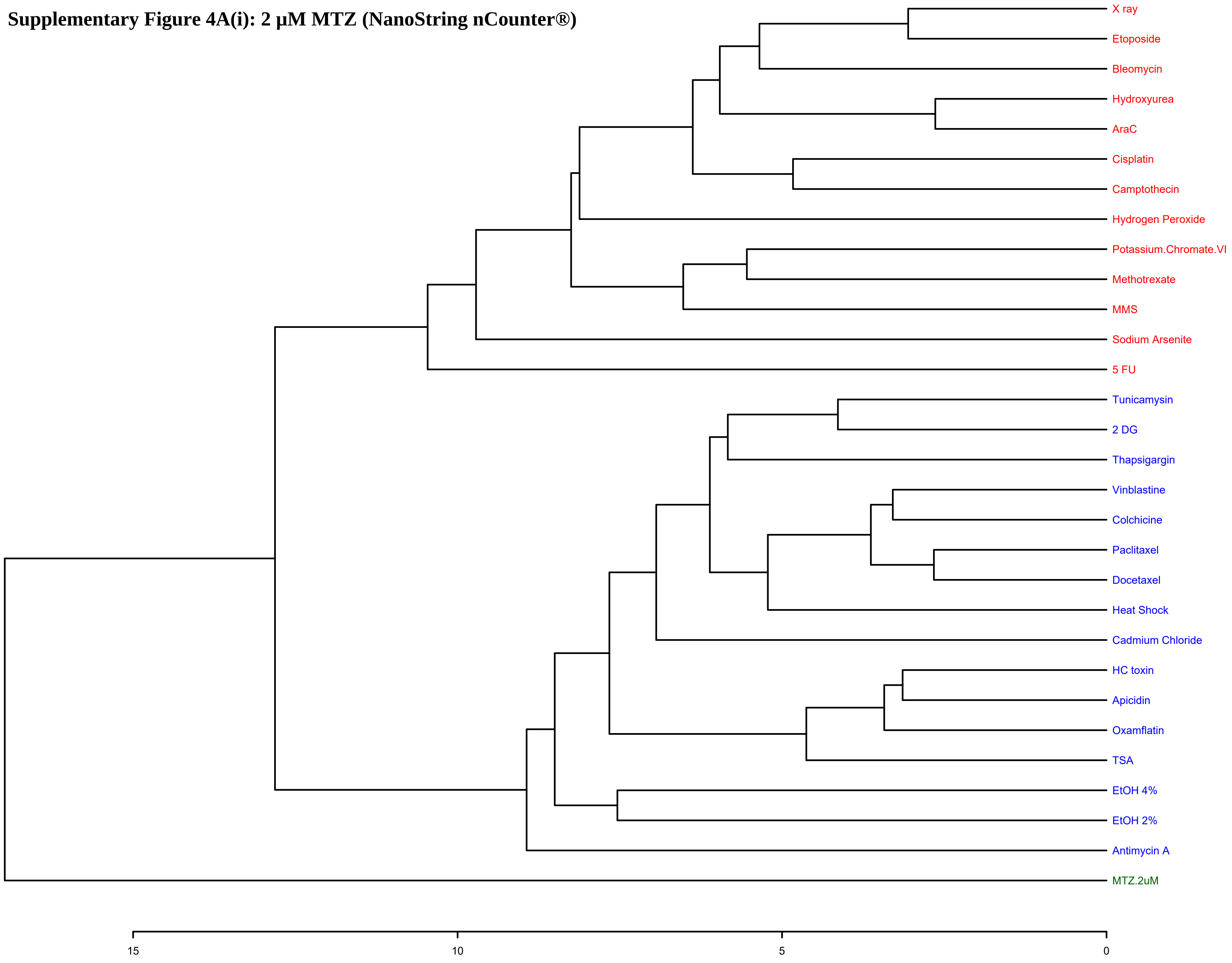

Supplementary Figure 4B(i): 3.9 µM MTZ (NanoString nCounter®)

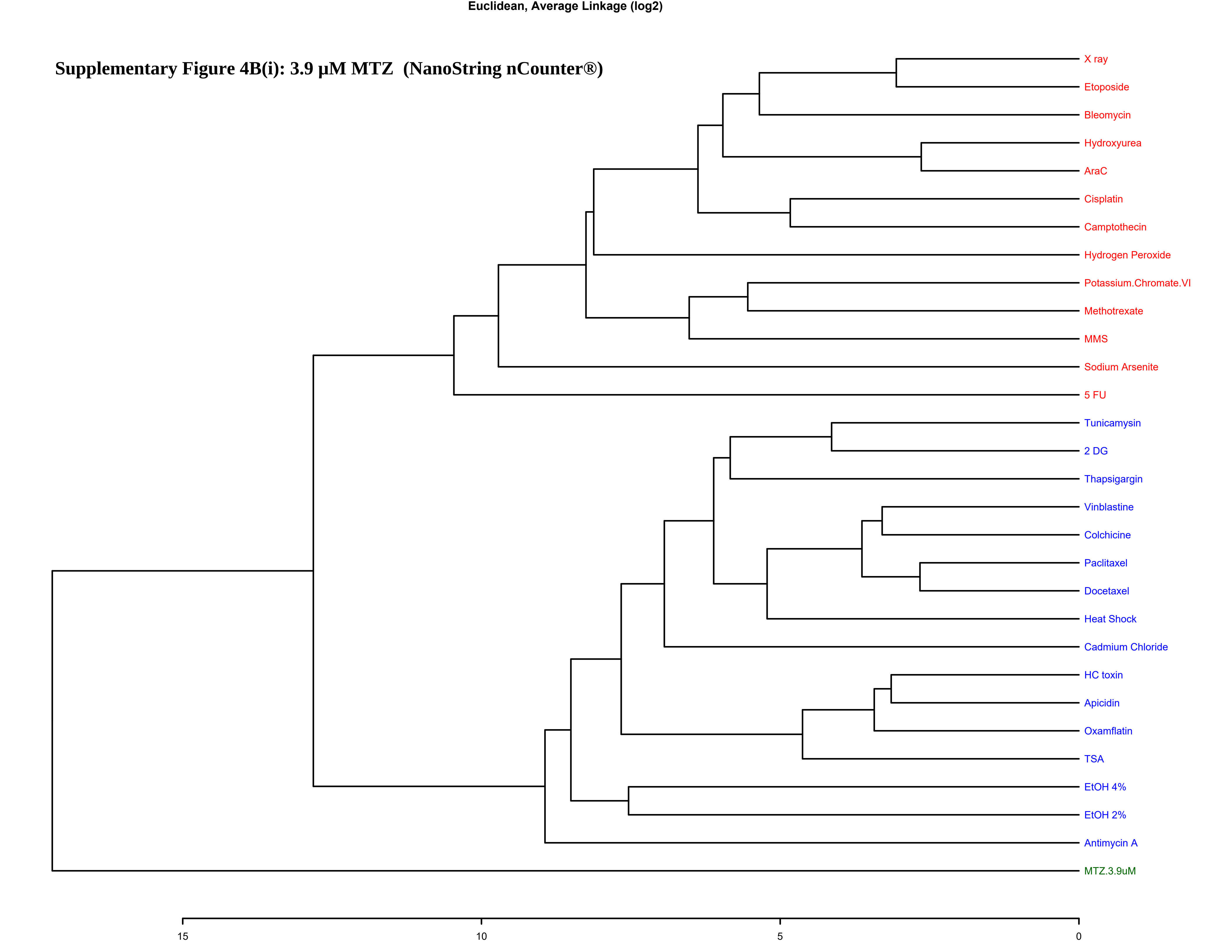

Supplementary Figure 4B(ii): 3.9 µM MTZ (BioSpyder TempO-Seq®)

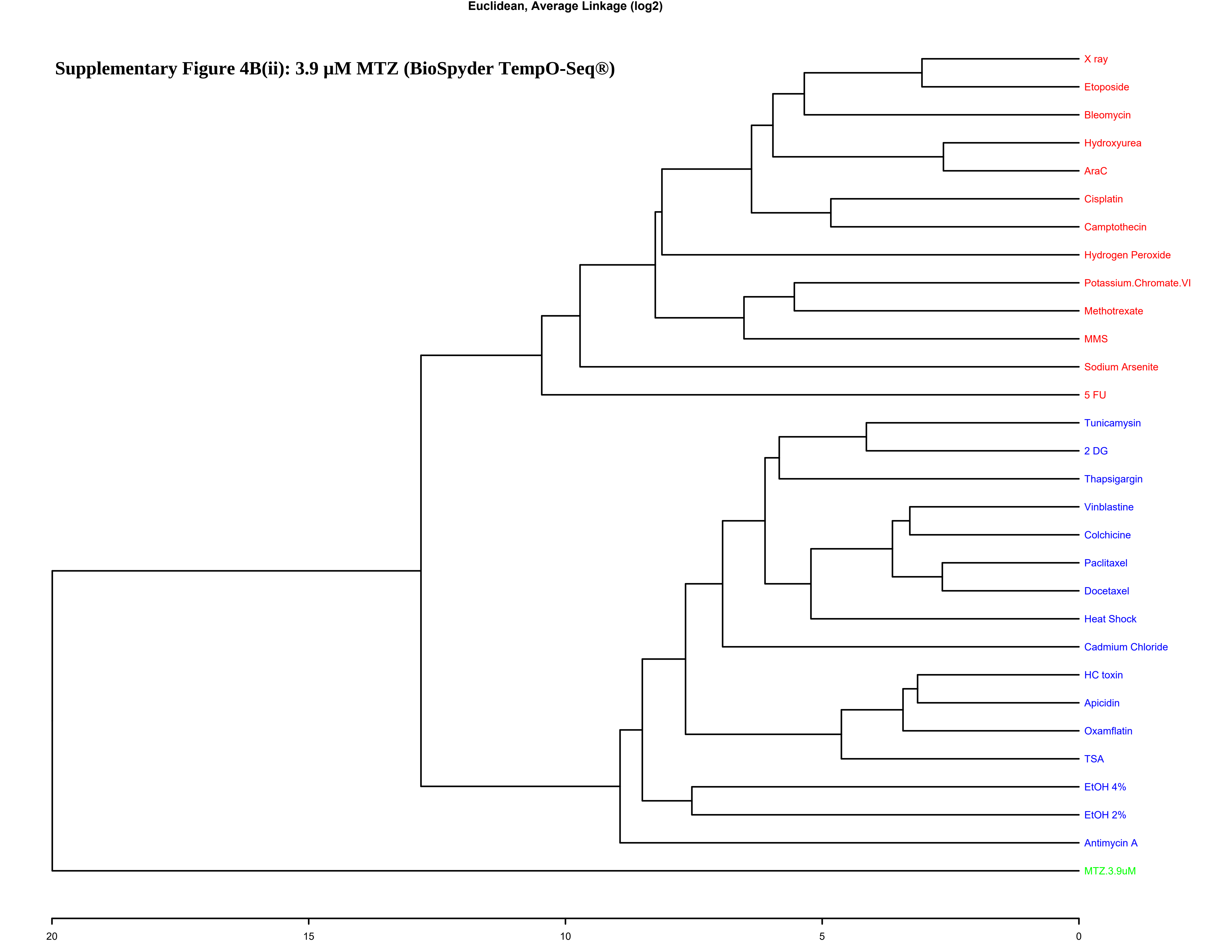

Supplementary Figure 4C(i): 7.8 µM MTZ (NanoString nCounter®)

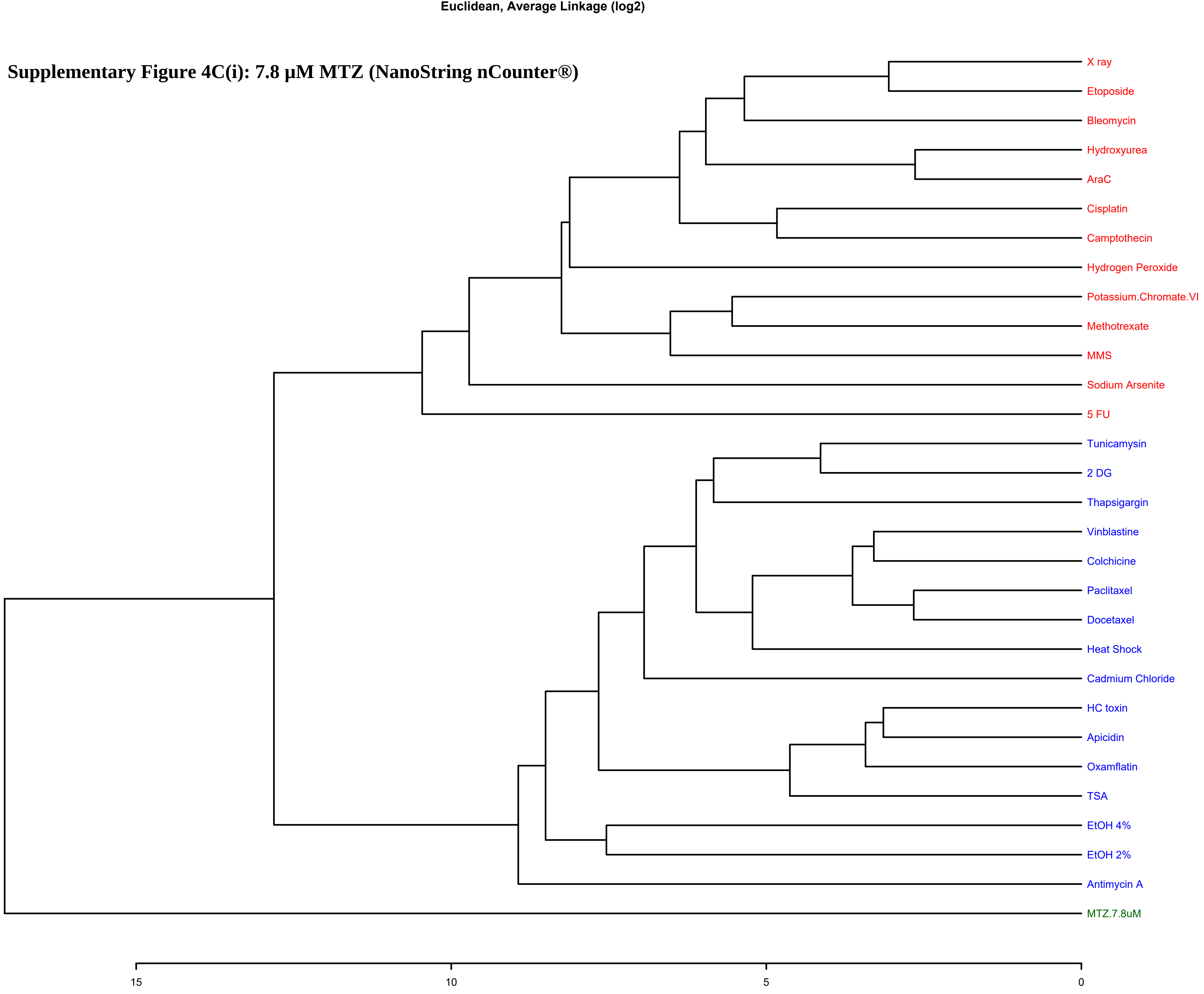

Euclidean, Average Linkage (log2)

Supplementary Figure 4C(ii): 7.8 µM MTZ (BioSpyder TempO-Seq®)

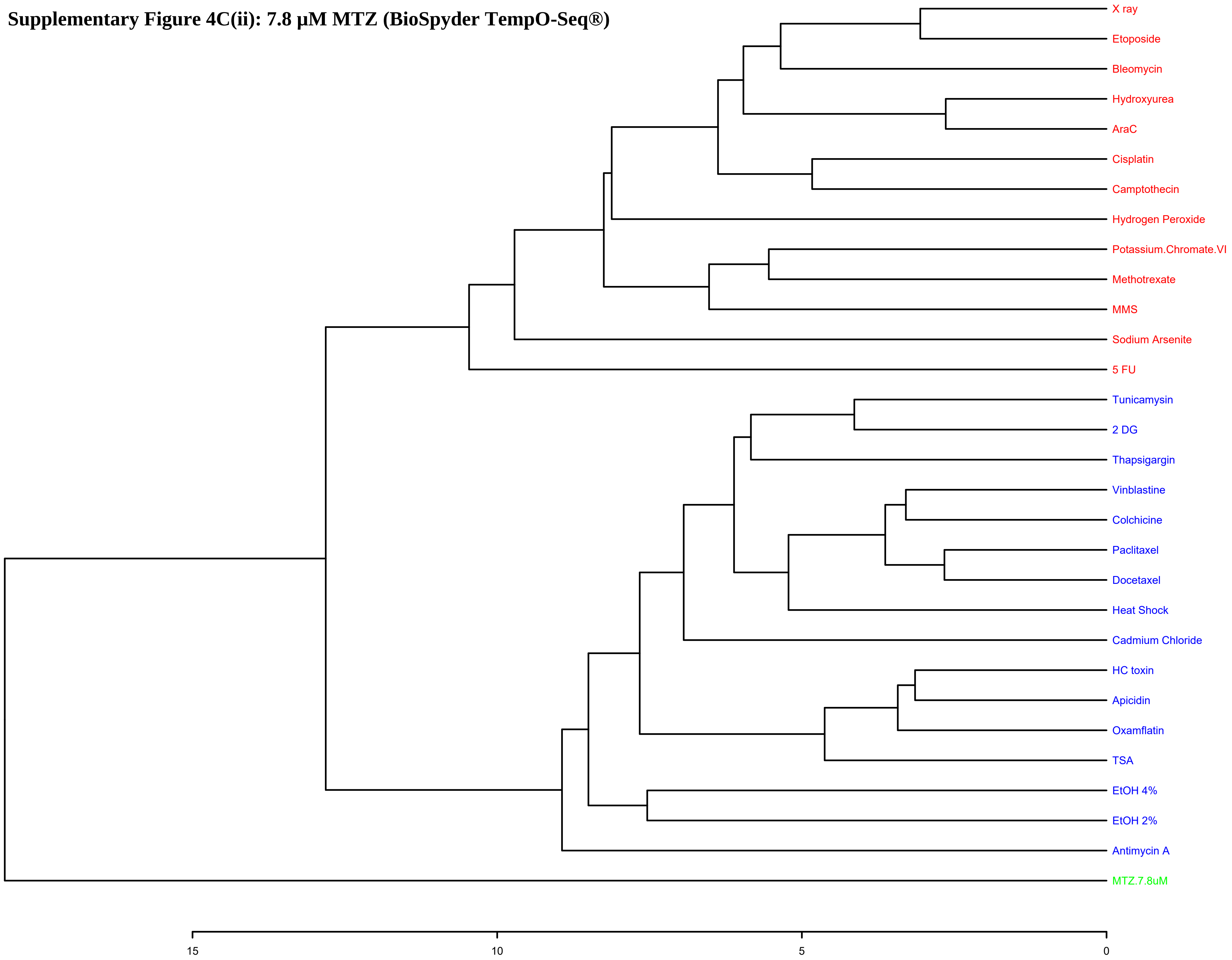

Supplementary Figure 4D(i): 15.6 μM MTZ (NanoString nCounter®)

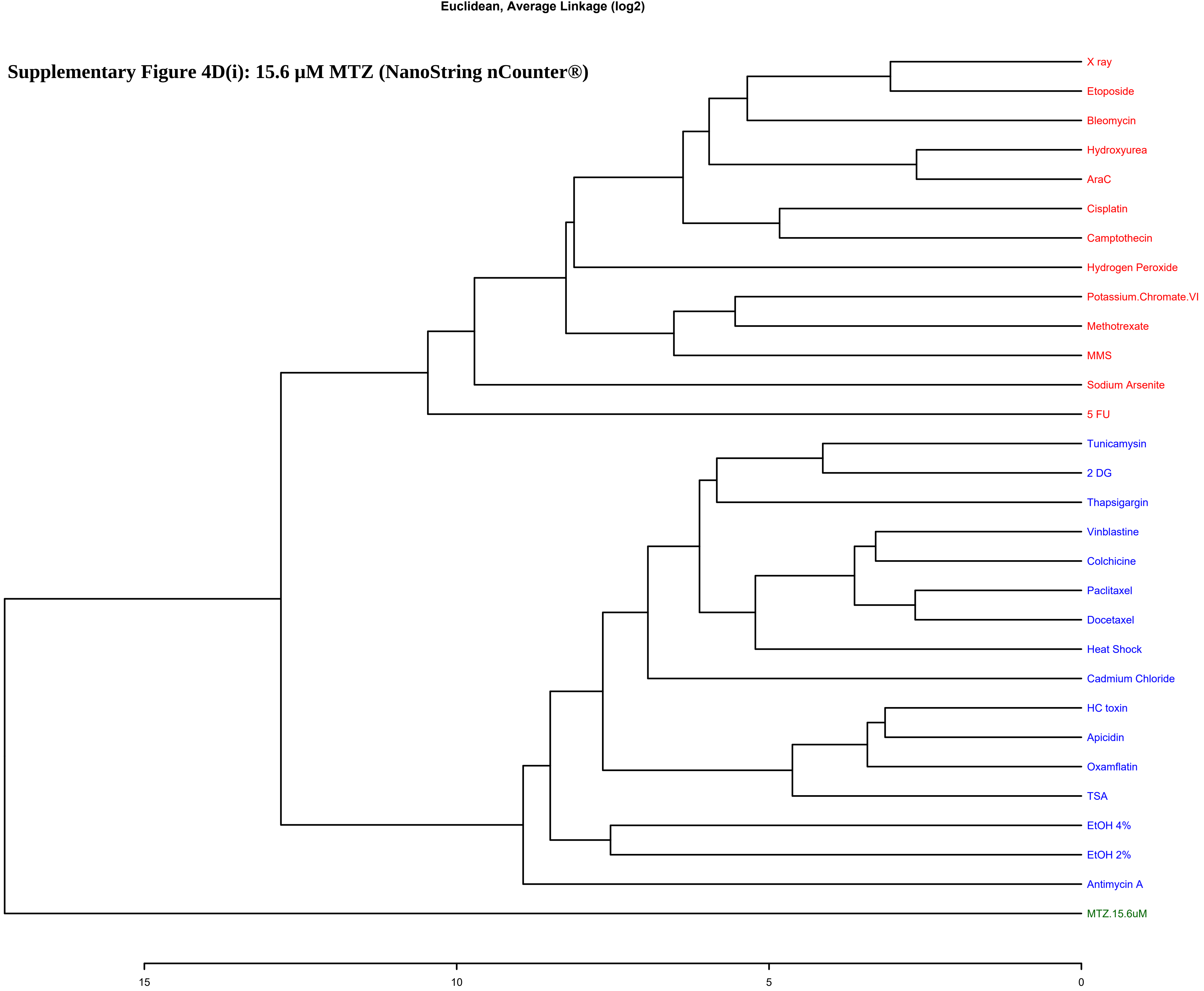

Supplementary Figure 4D(ii): 15.6 µM MTZ (BioSpyder TempO-Seq®)

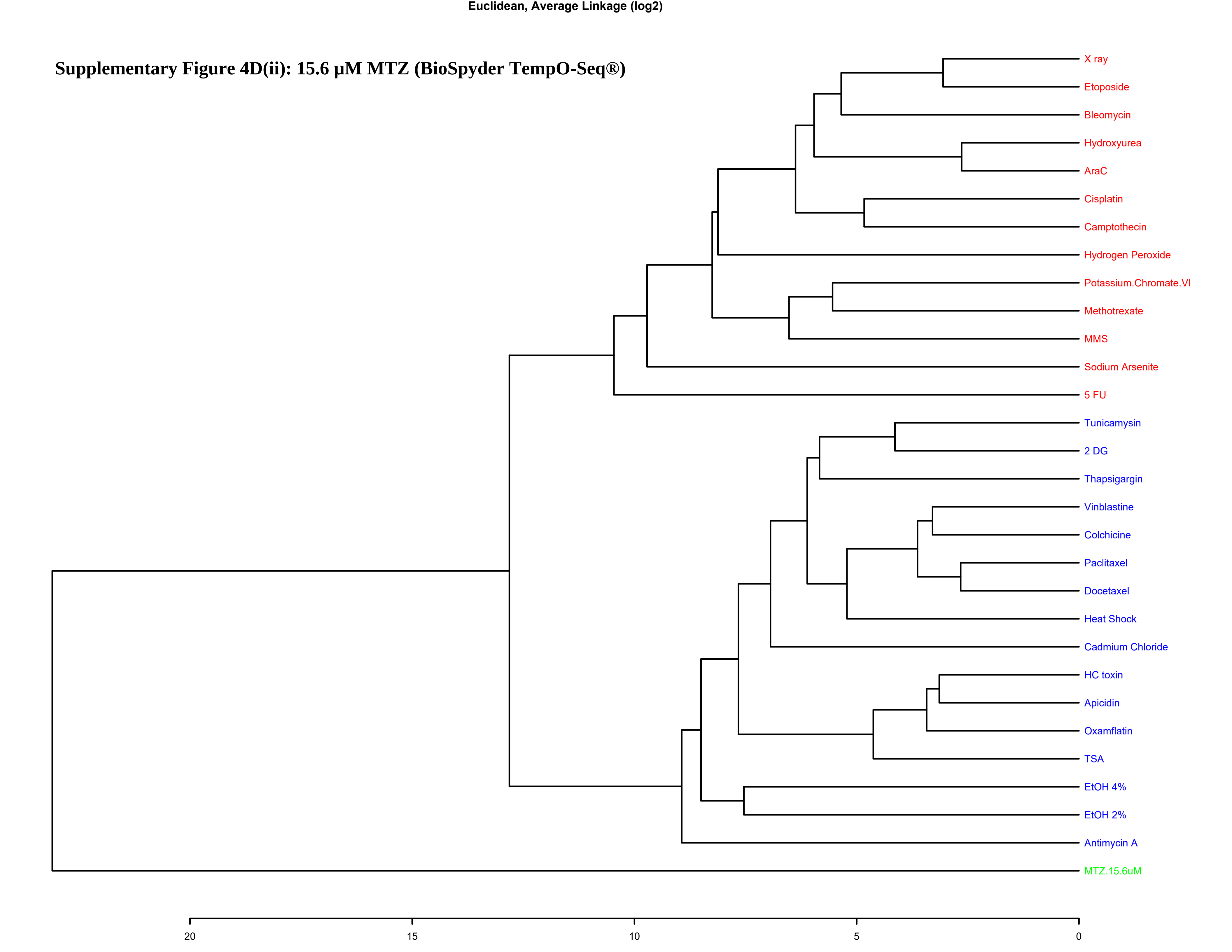

Euclidean, Average Linkage (log2)

Supplementary Figure 4E(i): 31.3 μM MTZ (NanoString nCounter®)

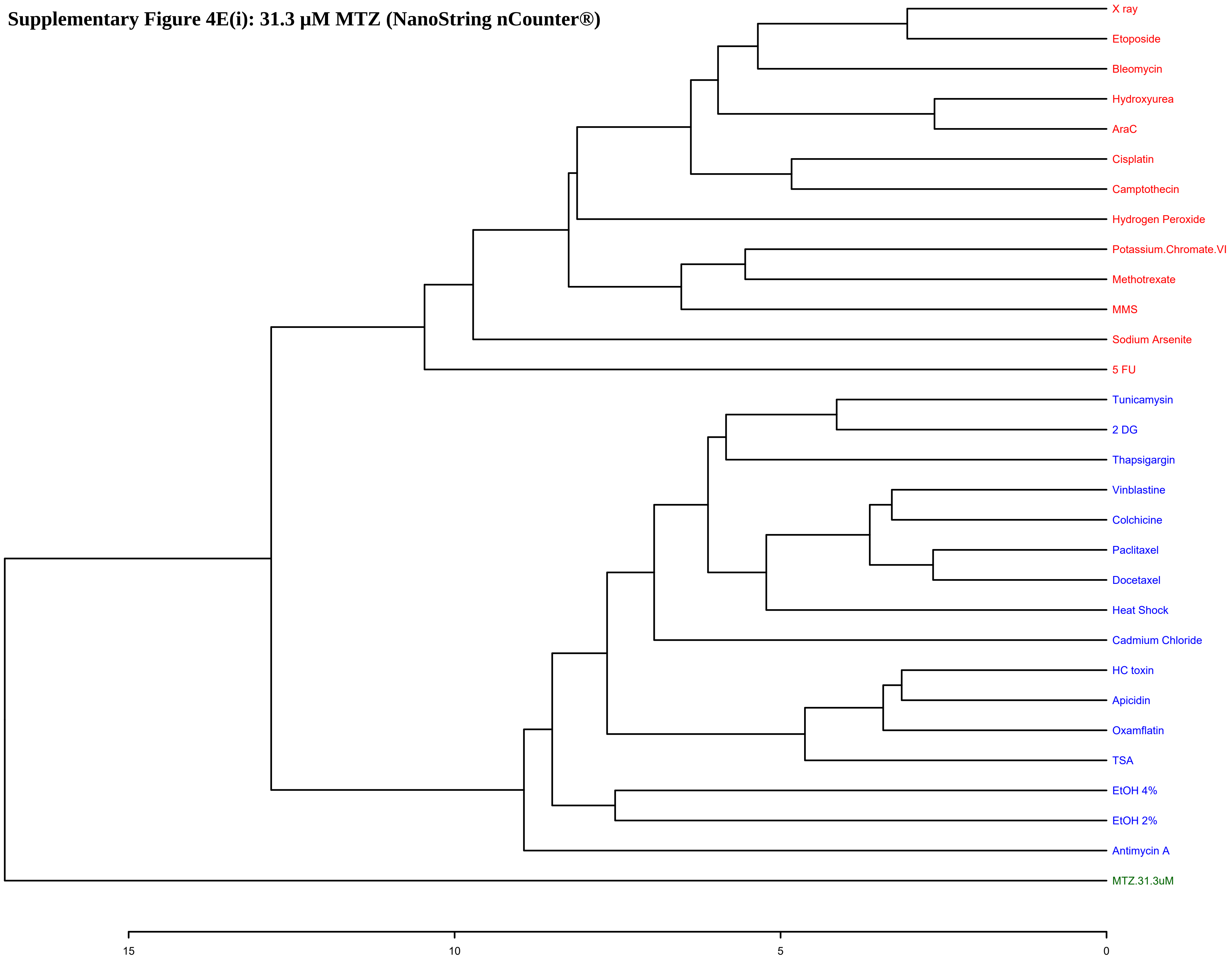

Supplementary Figure 4E(ii): 31.3 µM MTZ (BioSpyder TempO-Seq®)

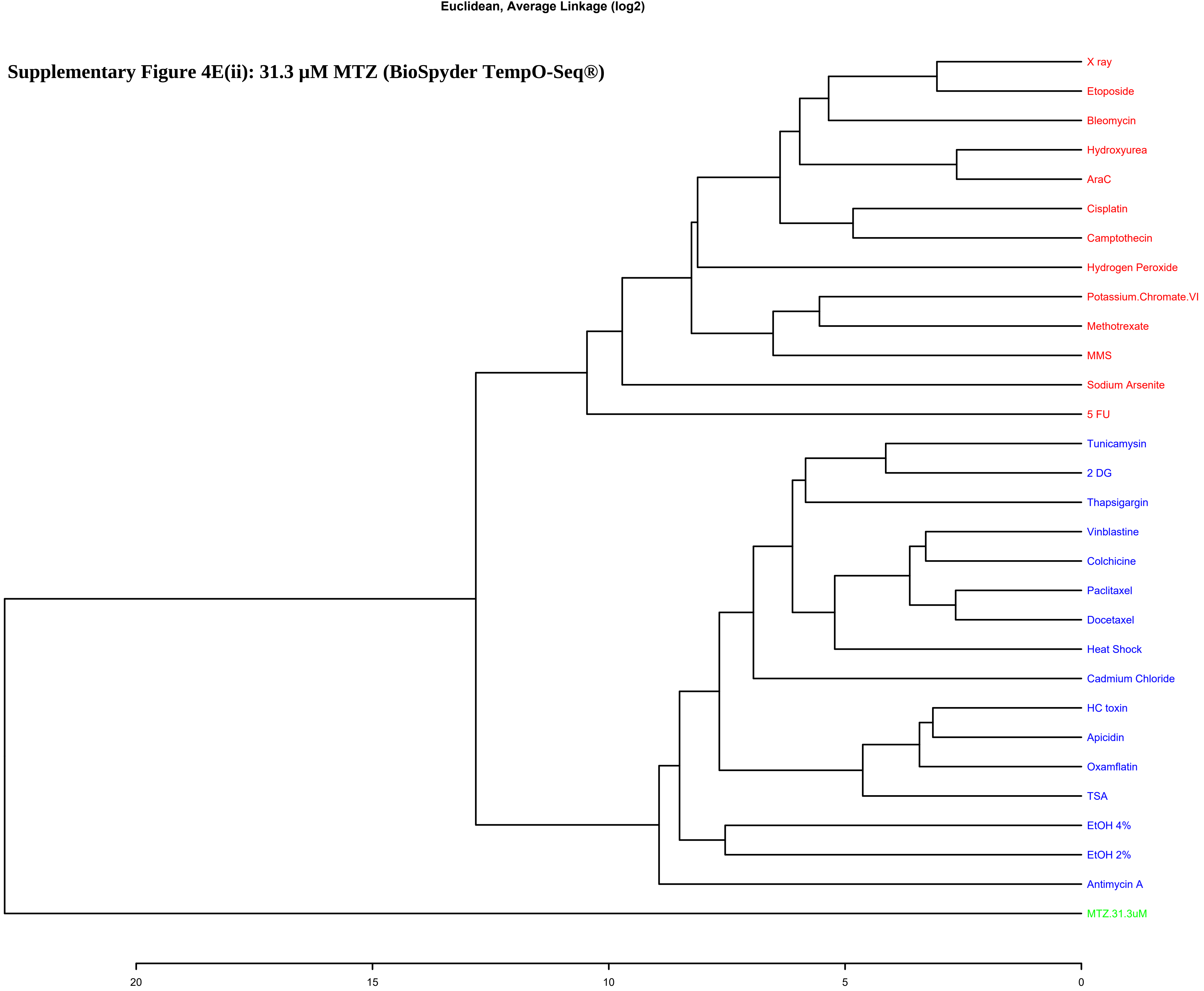

Supplementary Figure 4F(i): 62.5 µM MTZ (NanoString nCounter®)

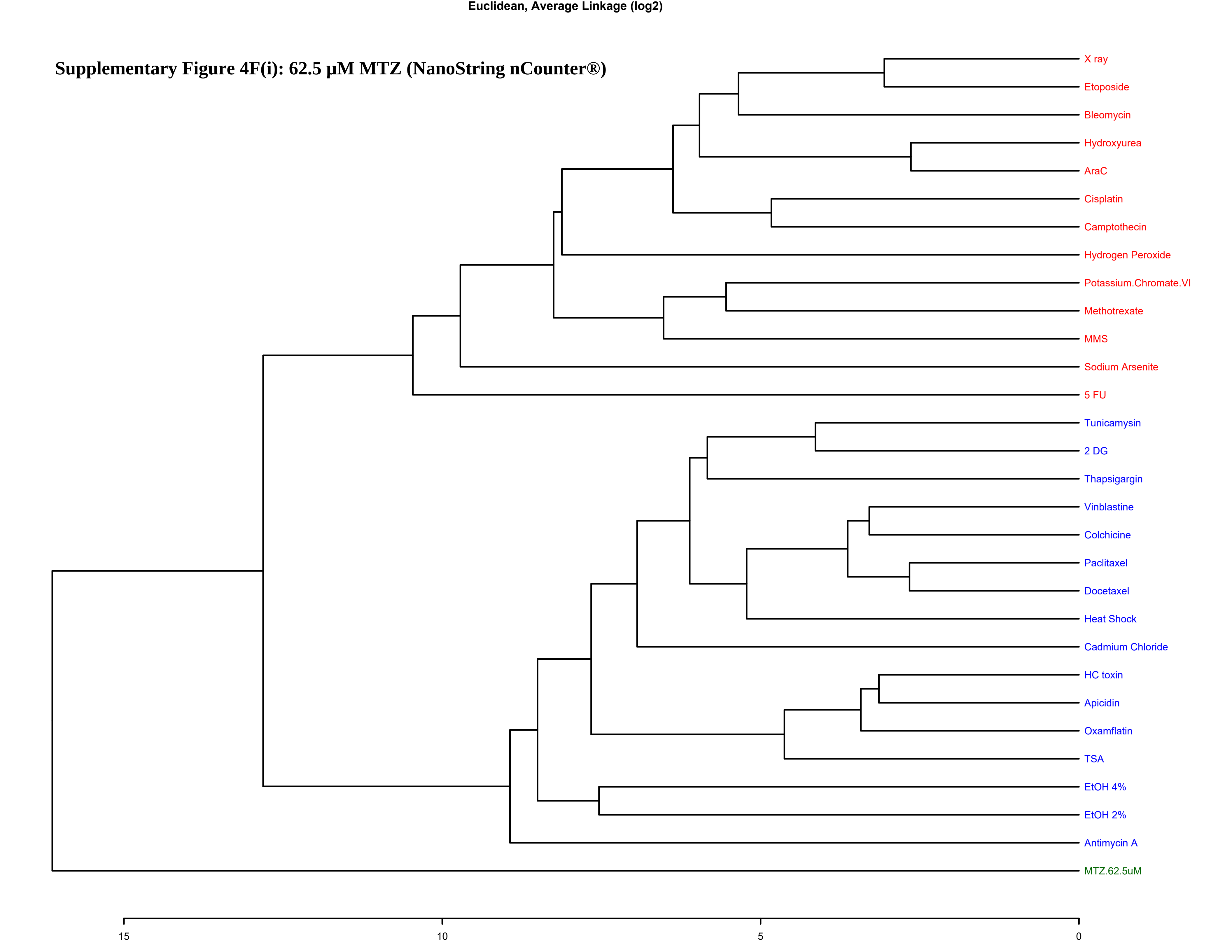

Supplementary Figure 4F(ii): 62.5 µM MTZ (BioSpyder TempO-Seq®)

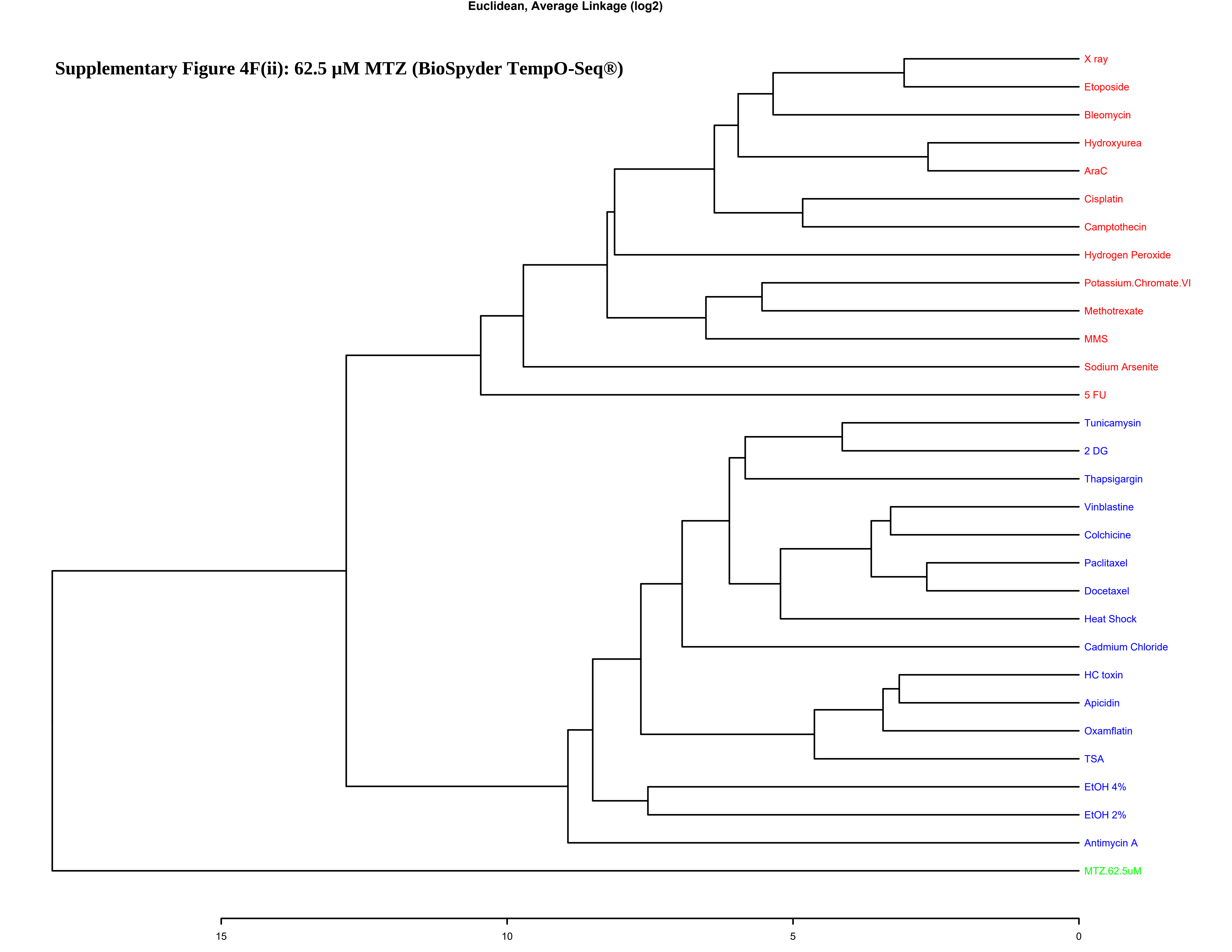

Euclidean, Average Linkage (log2)

Supplementary Figure 4G(i): 125 µM MTZ (NanoString nCounter®)

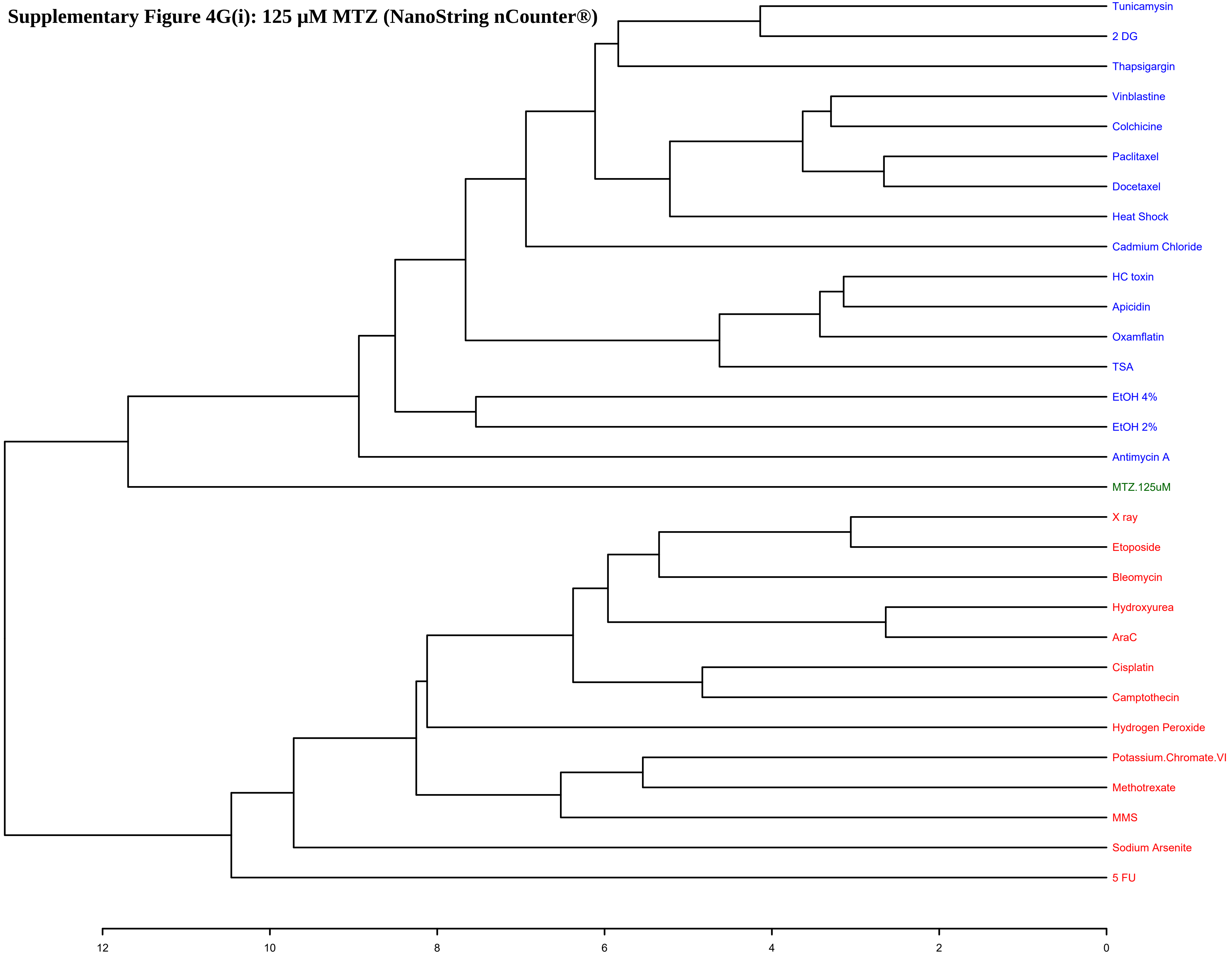

Euclidean, Average Linkage (log2)

Supplementary Figure 4G(ii): 125 µM MTZ (BioSpyder TempO-Seq®)

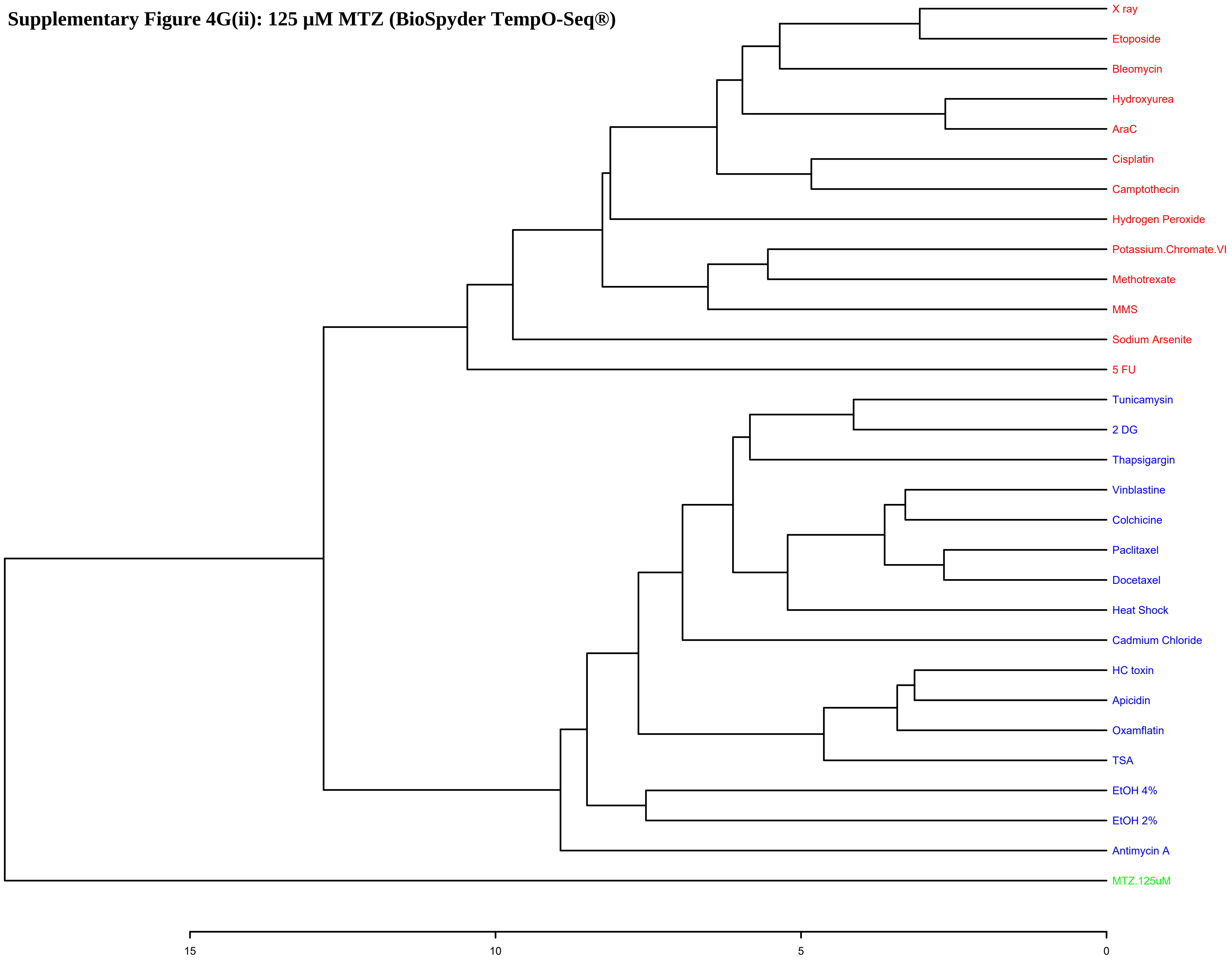

Supplementary Figure 4H(i): 125 µM MTZ (NanoString nCounter®)

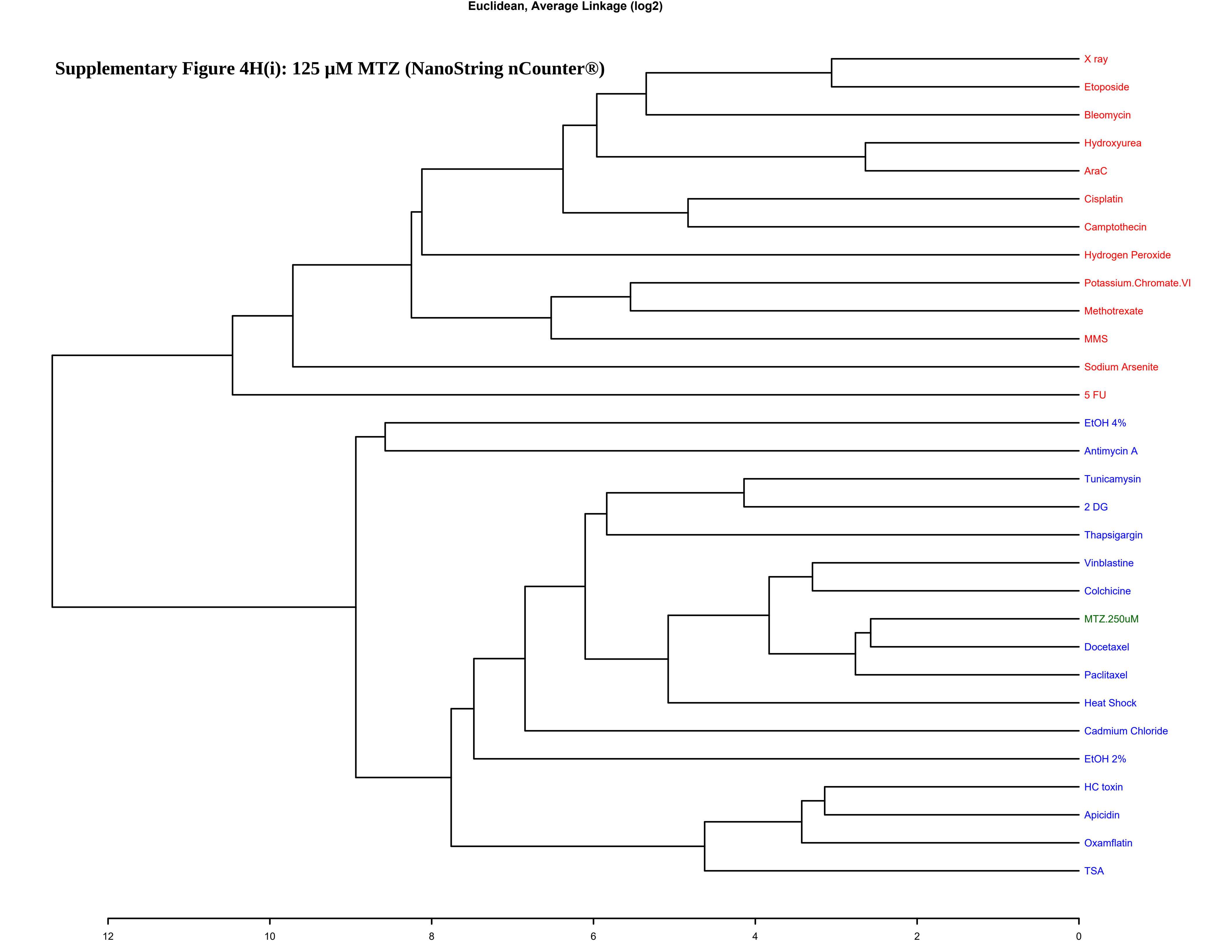

Euclidean, Average Linkage (log2)

Supplementary Figure 4H(ii): 125 µM MTZ (BioSpyder TempO-Seq®)

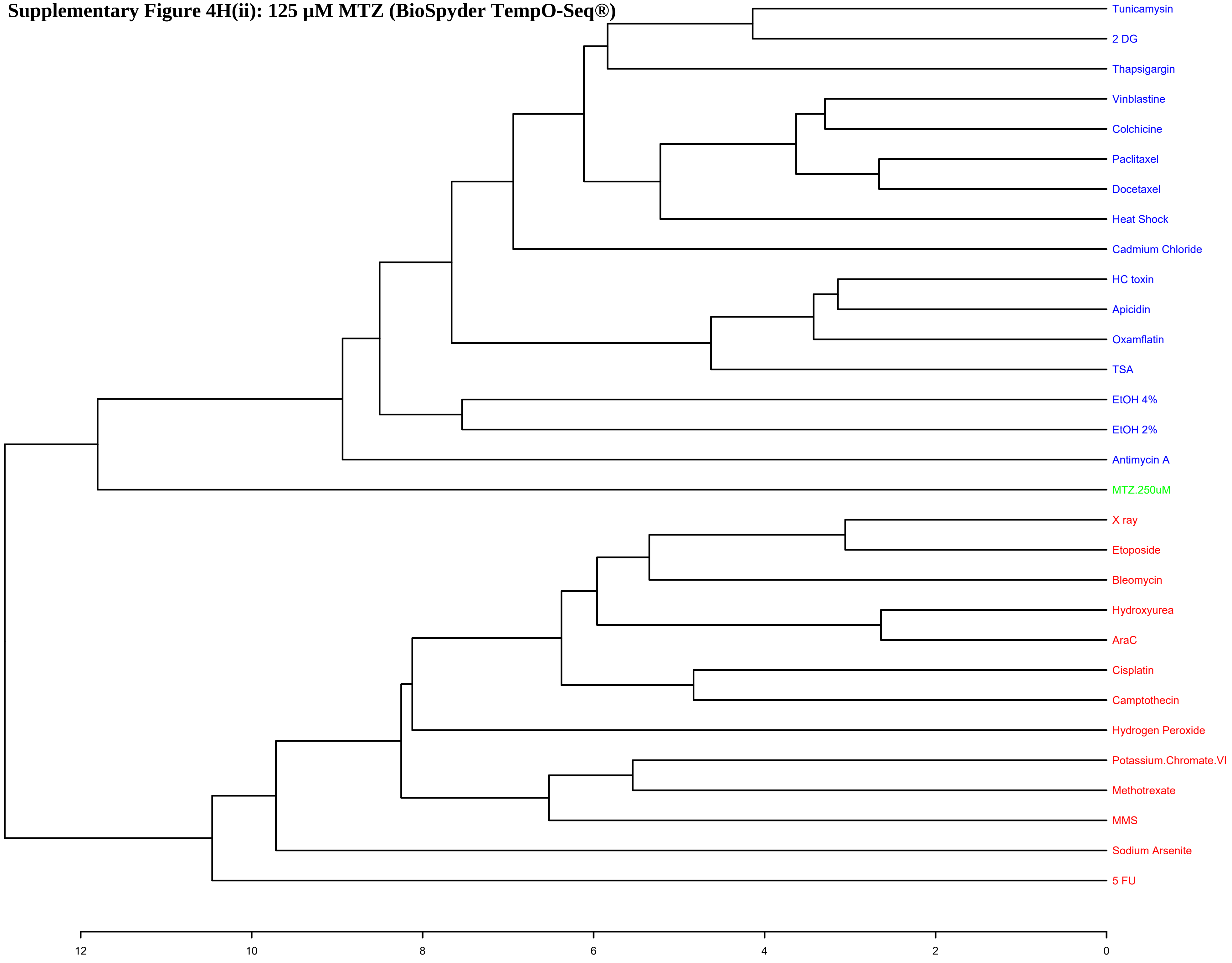

Supplementary Figure 5A(i): 2 µM NOV (NanoString nCounter®)

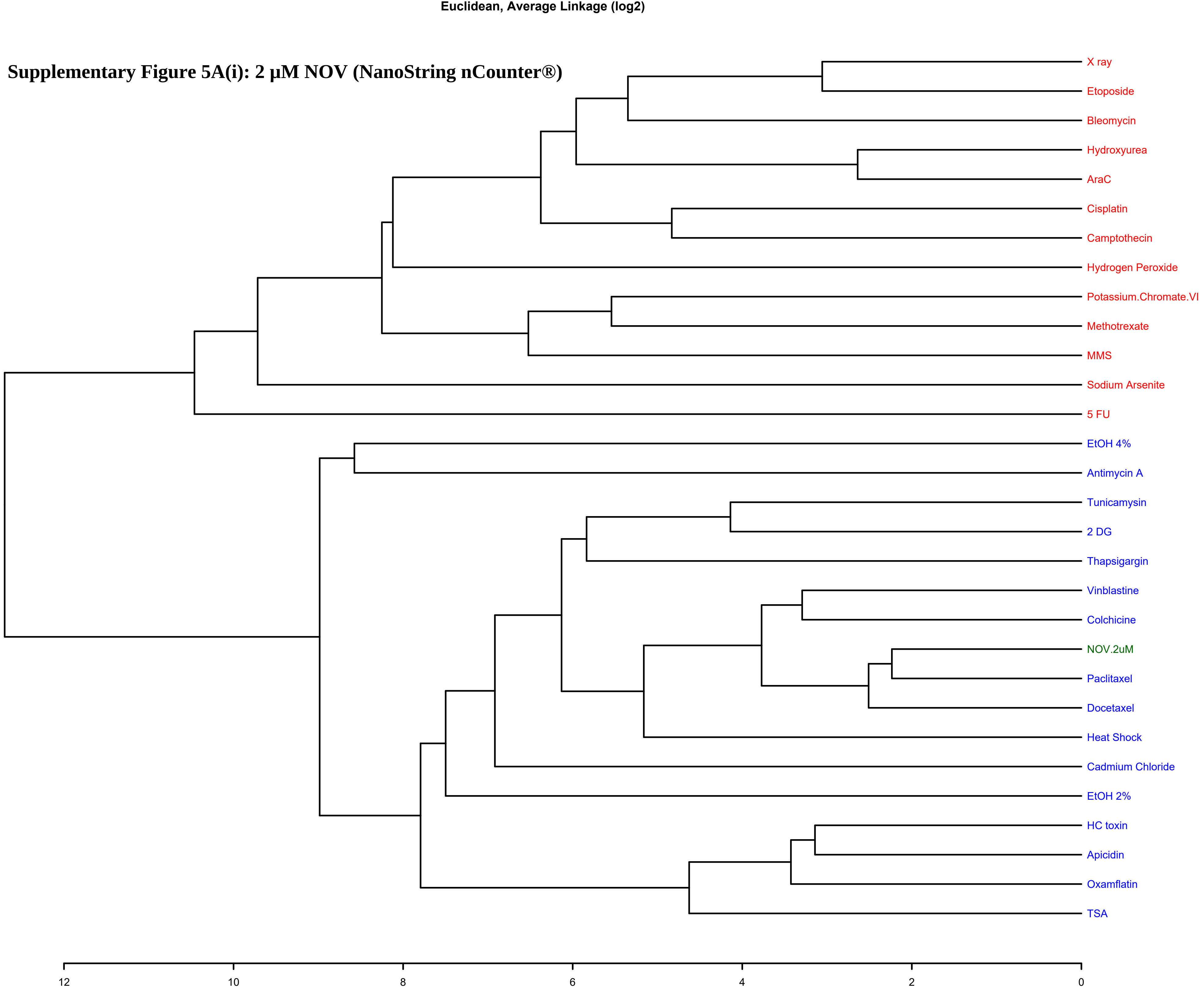

Supplementary Figure 5B(i): 3.9 µM NOV (NanoString nCounter®)

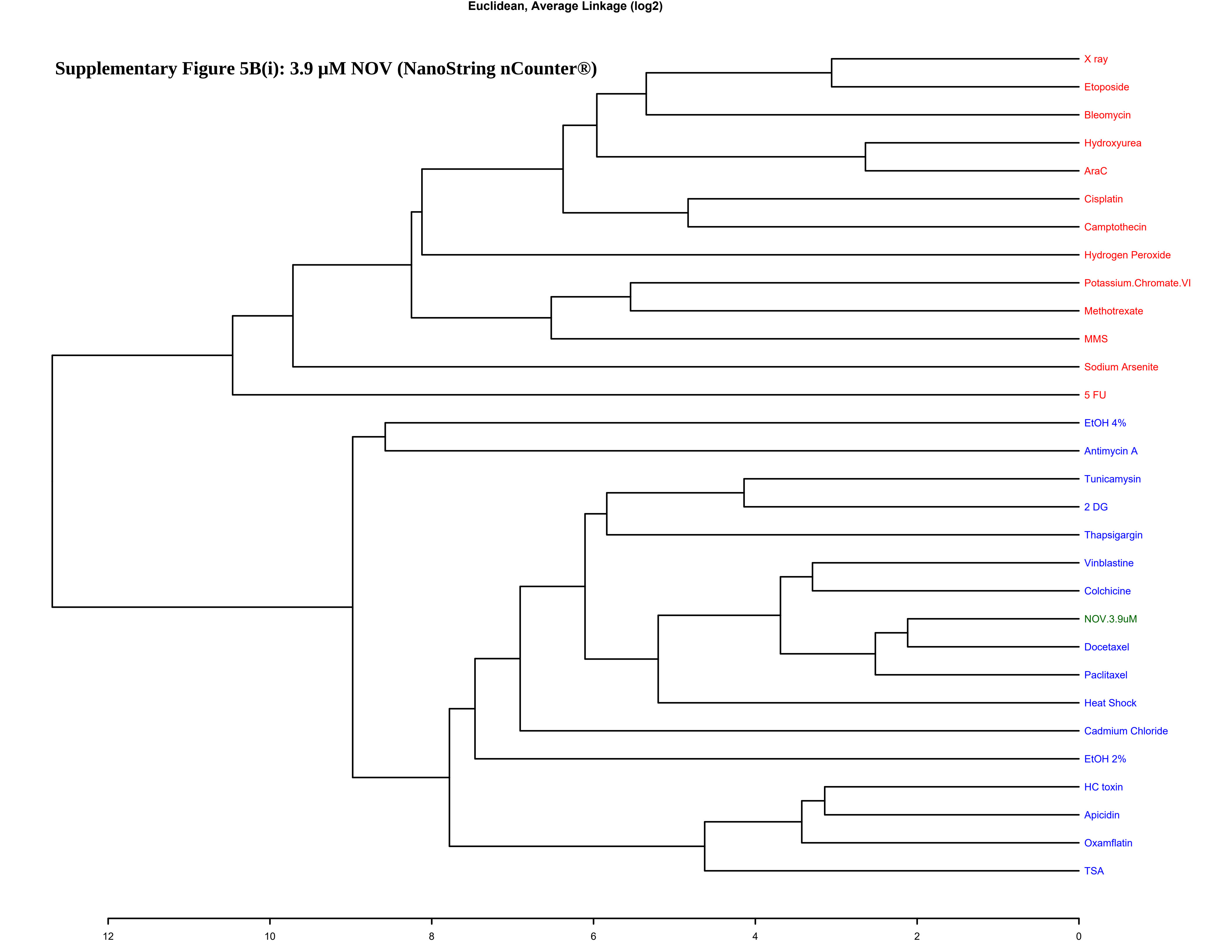

Supplementary Figure 5B(ii): 3.9 µM NOV (BioSpyder TempO-Seq®)

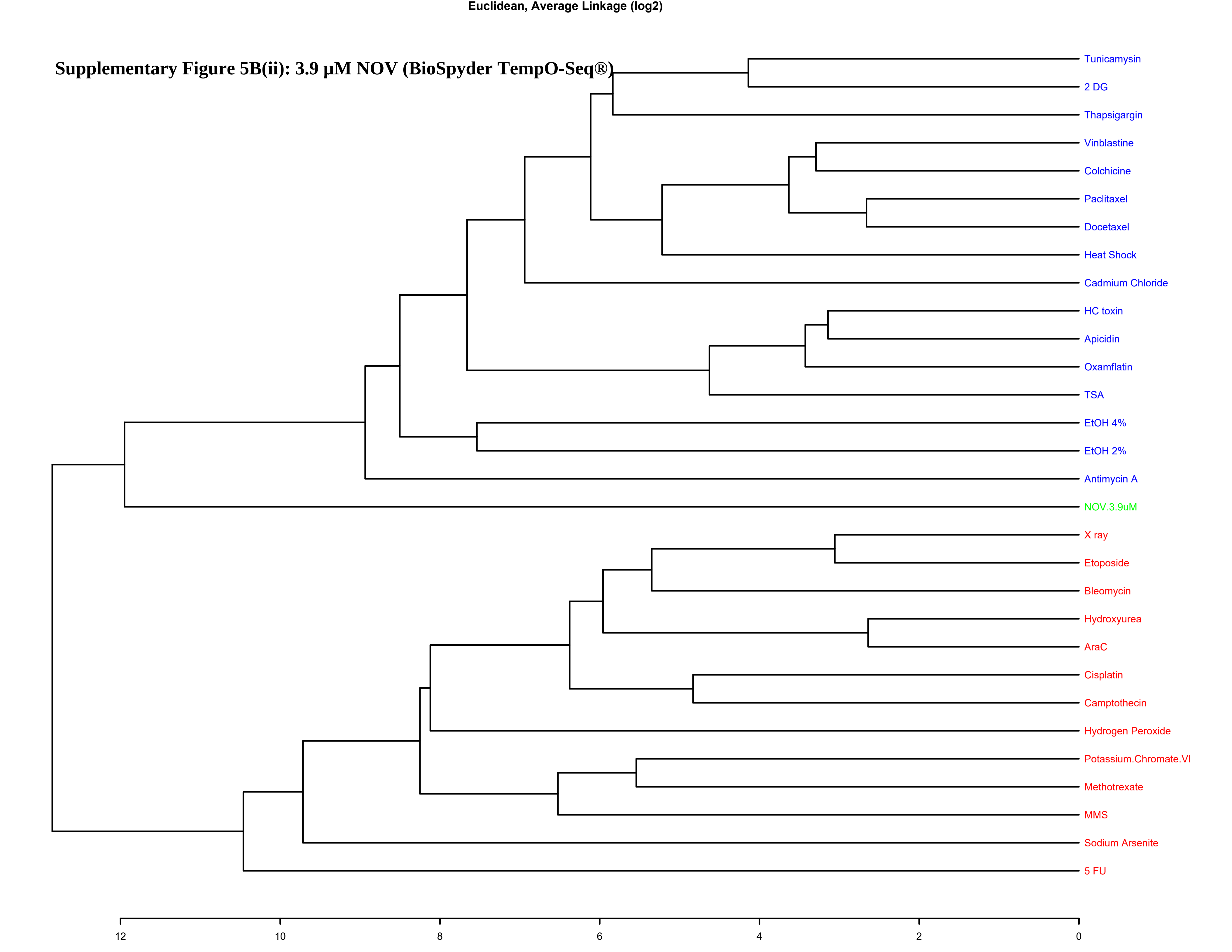

Euclidean, Average Linkage (log2)

Supplementary Figure 5C(i): 7.8 μM NOV (NanoString nCounter®)

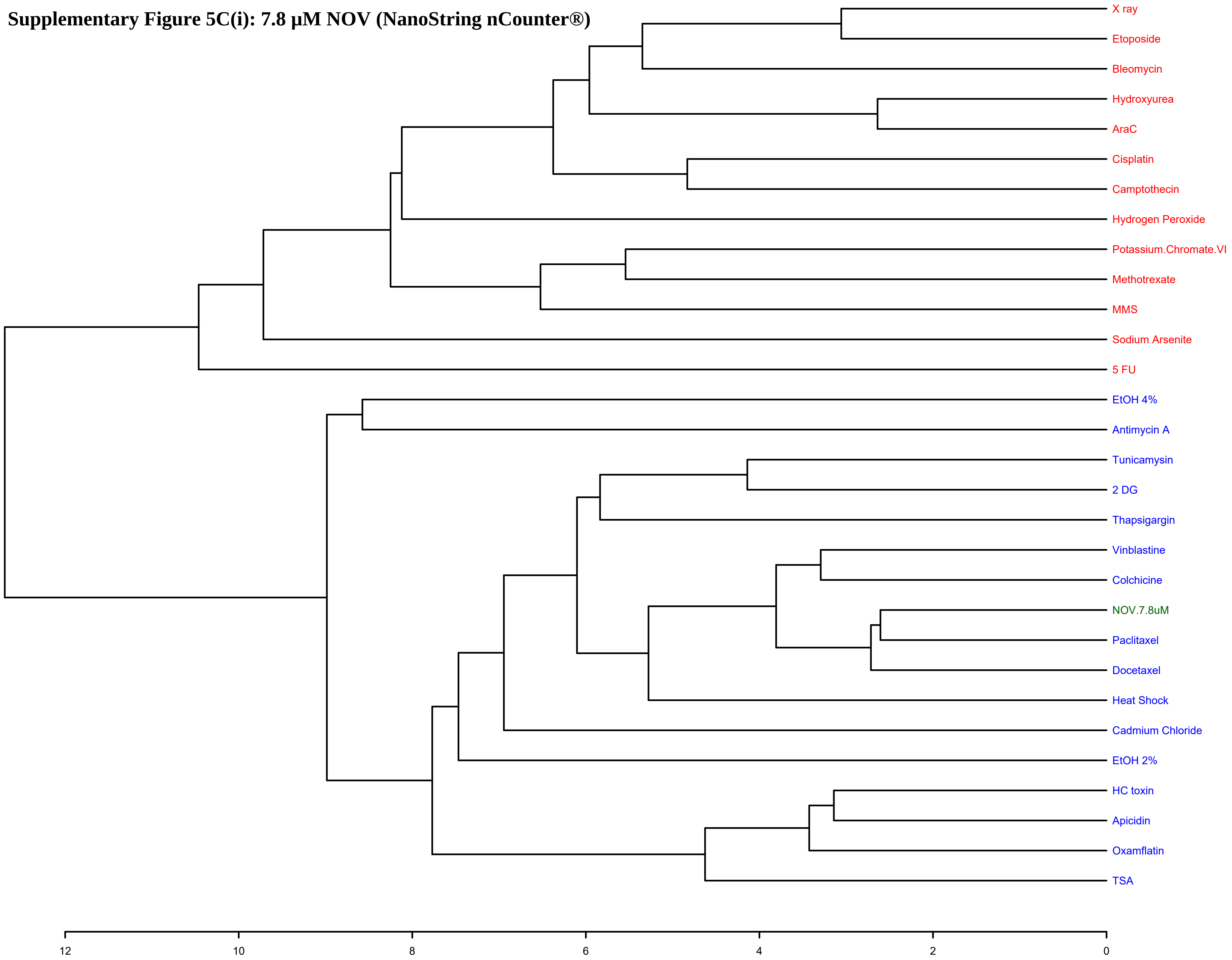

Supplementary Figure 5C(ii): 7.8 µM NOV (BioSpyder TempO-Seq®)

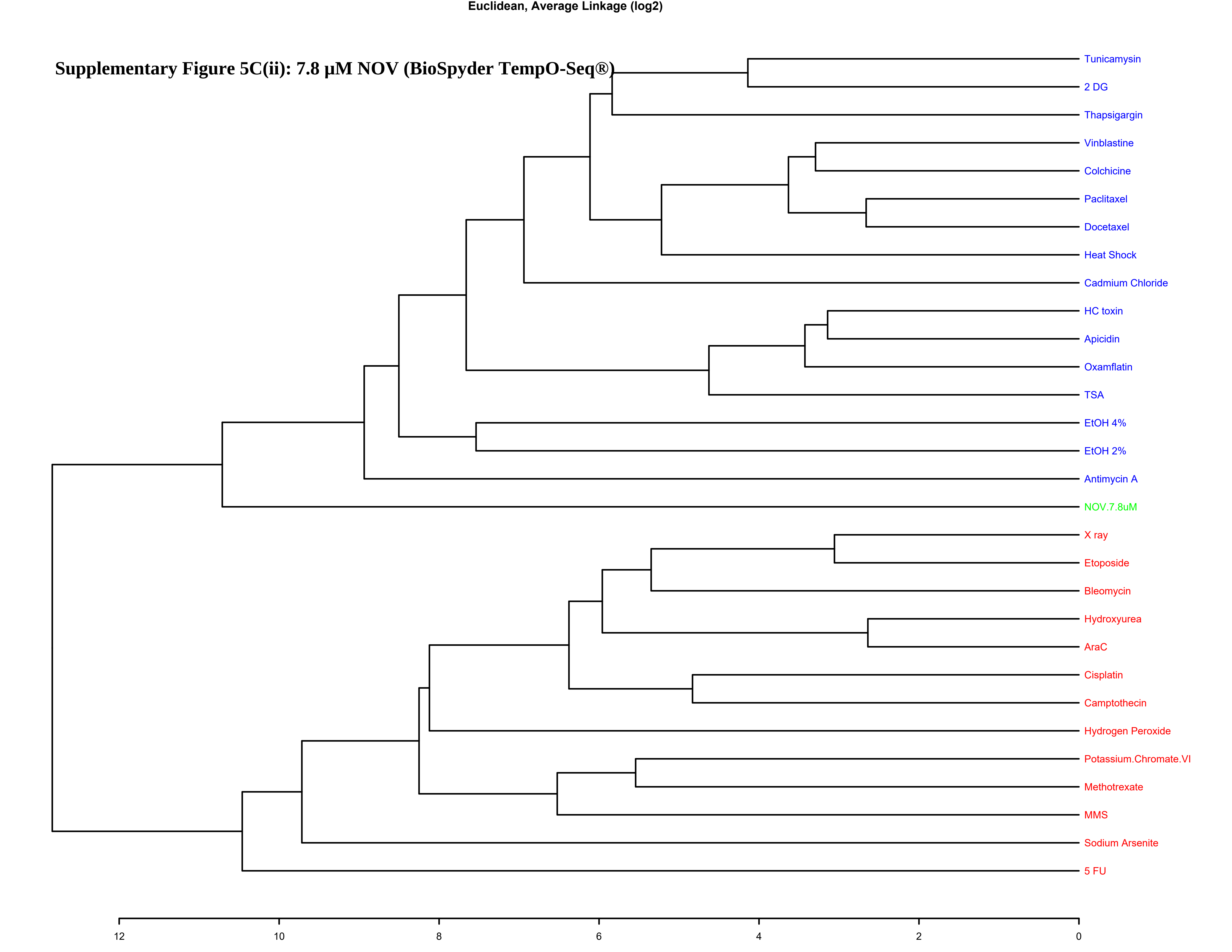

Euclidean, Average Linkage (log2)

Supplementary Figure 5D(i): 15.6 μM NOV (NanoString nCounter®)

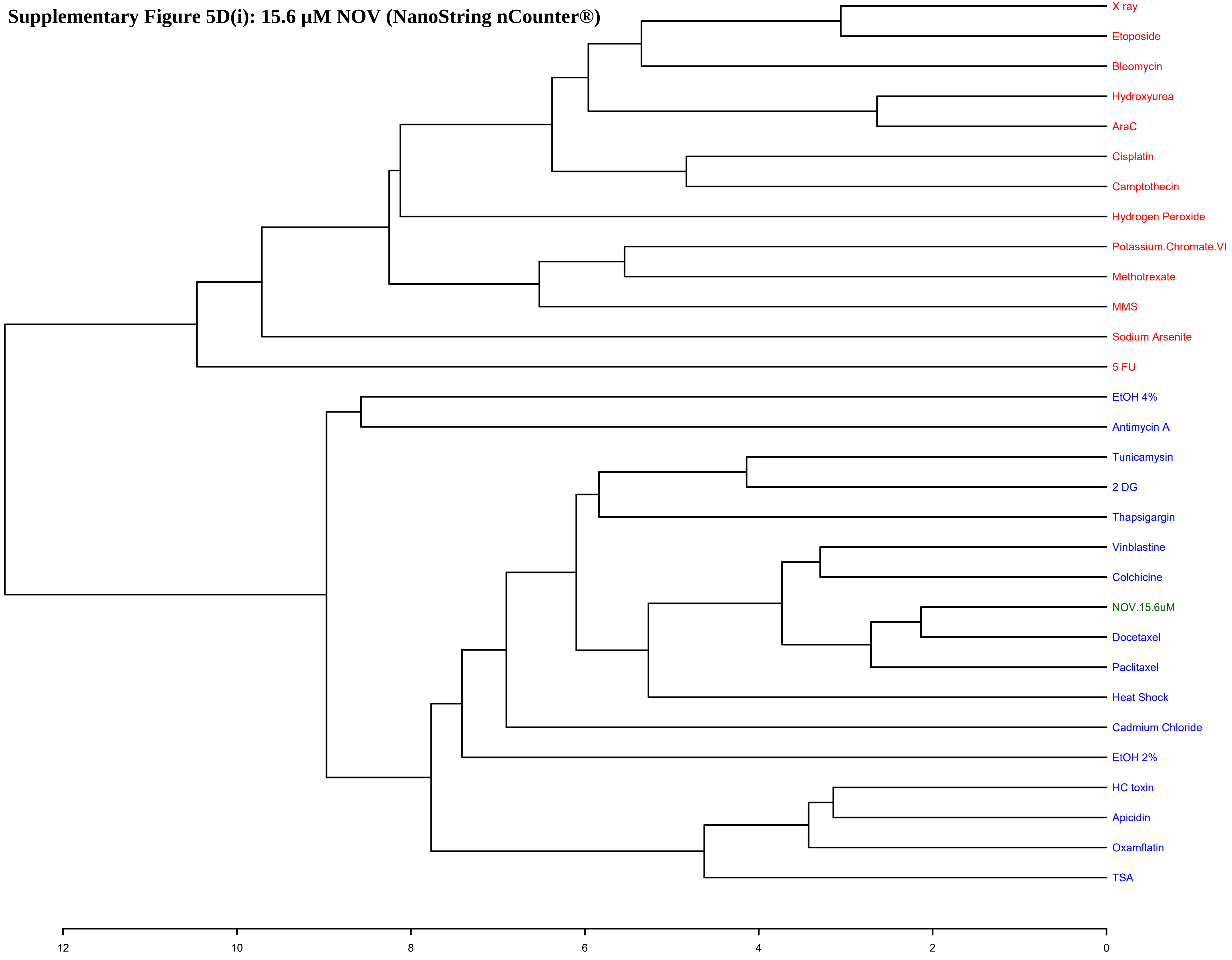

Supplementary Figure 5D(ii): 15.6 μM NOV (BioSpyder TempO-Seq®)

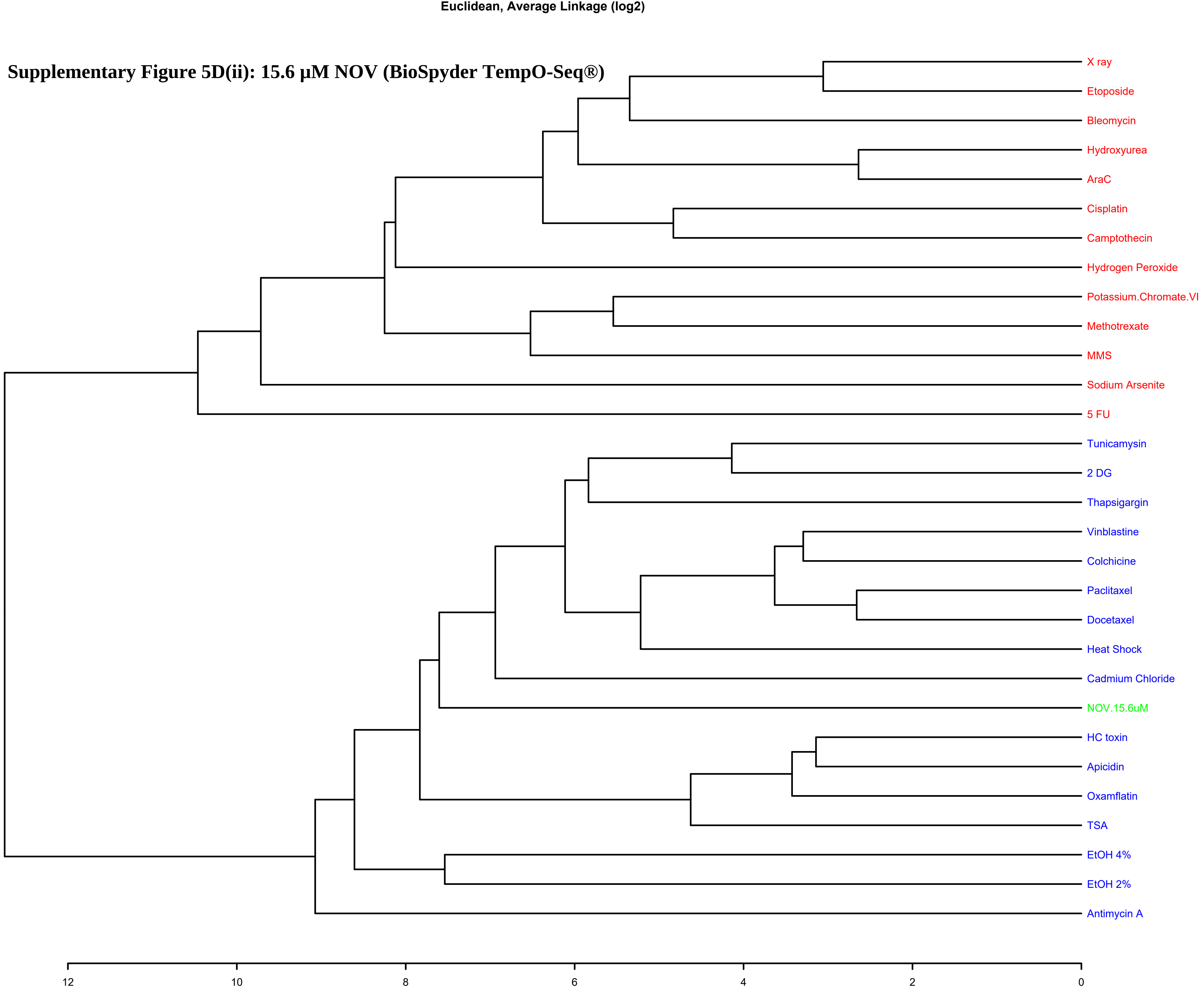

Supplementary Figure 5E(i): 31.3 μM NOV (NanoString nCounter®)

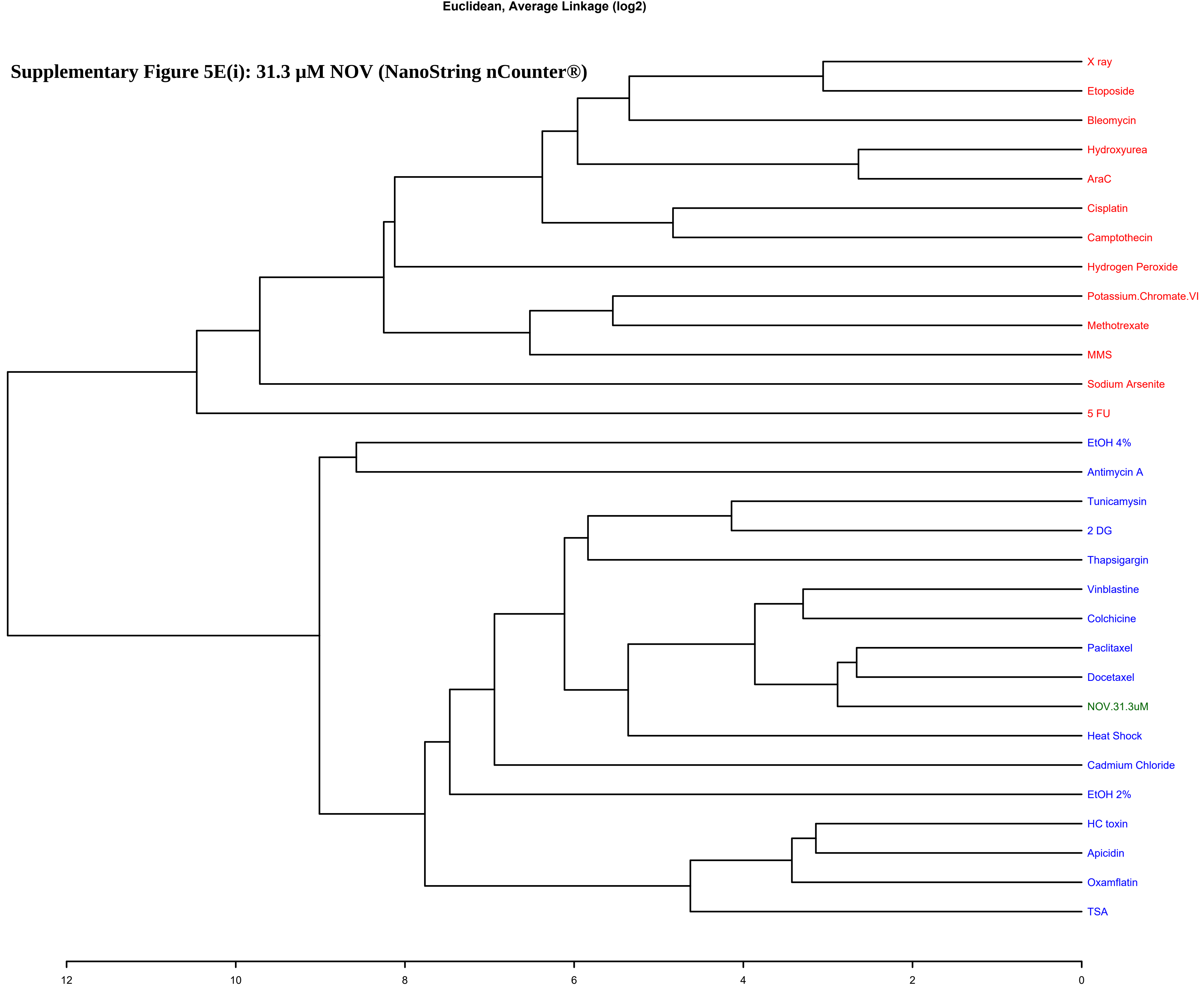

Supplementary Figure 5E(ii): 31.3 μM NOV (BioSpyder TempO-Seq®)

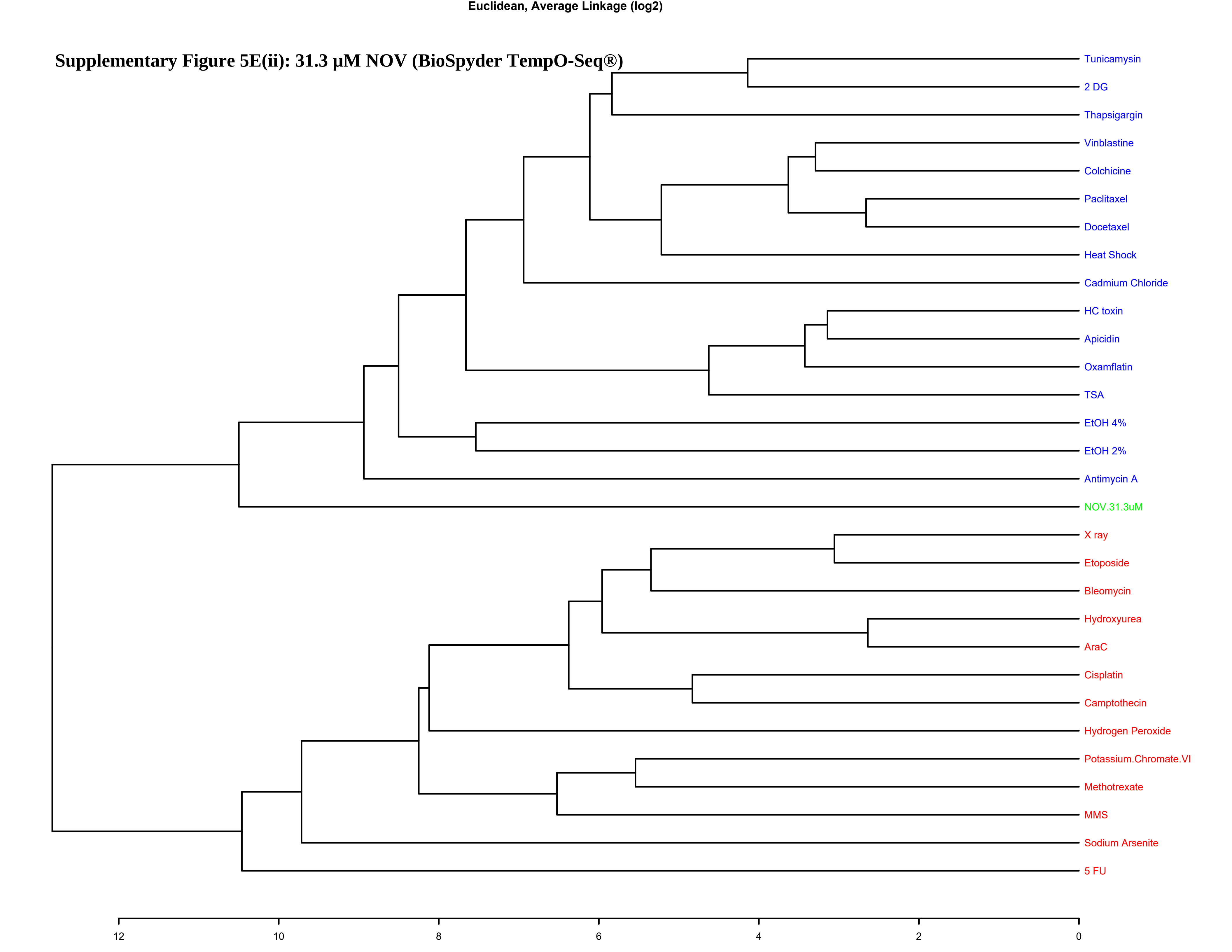

Supplementary Figure 5F(i): 62.5 µM NOV (NanoString nCounter®)

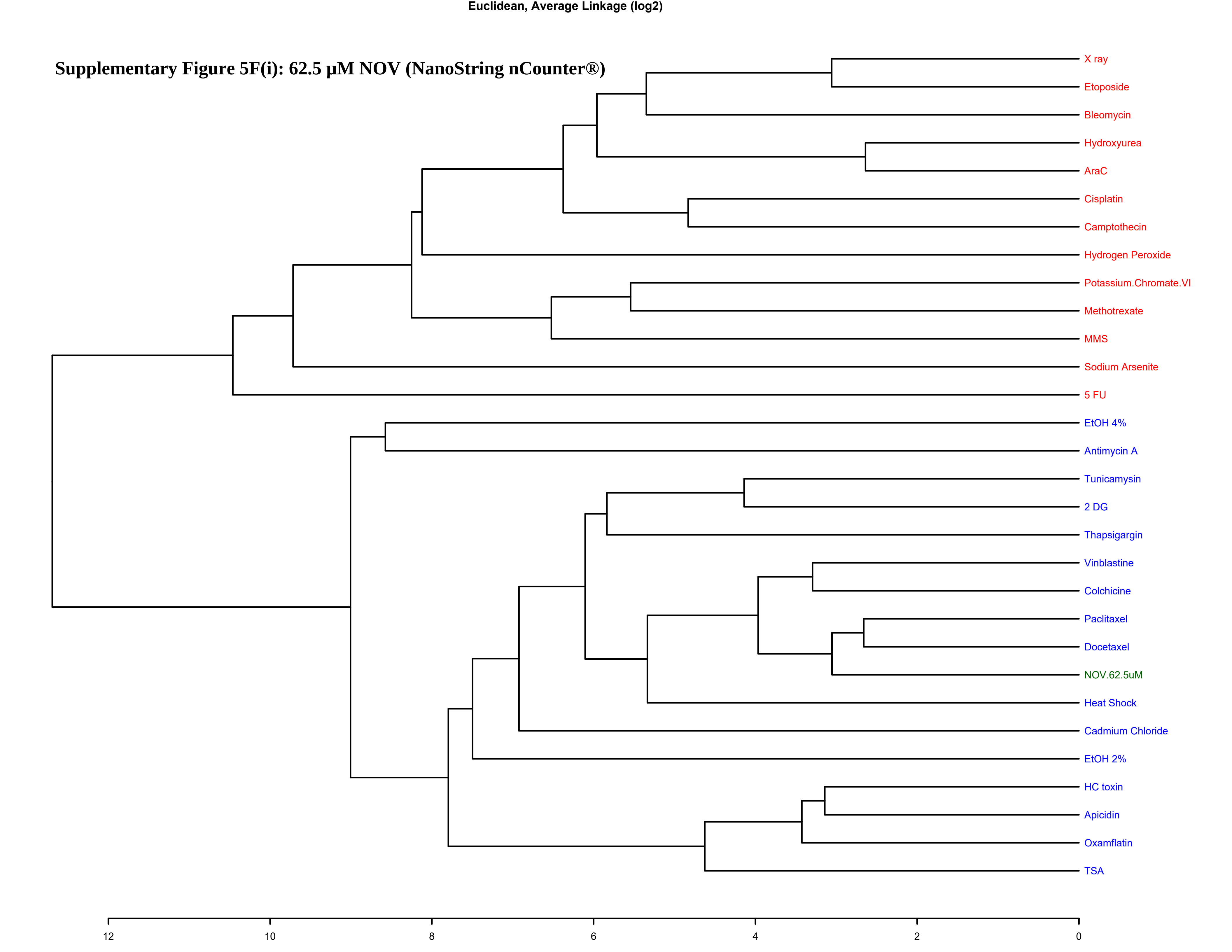

Euclidean, Average Linkage (log2)

Supplementary Figure 5F(ii): 62.5 µM NOV (BioSpyder TempO-Seq®)

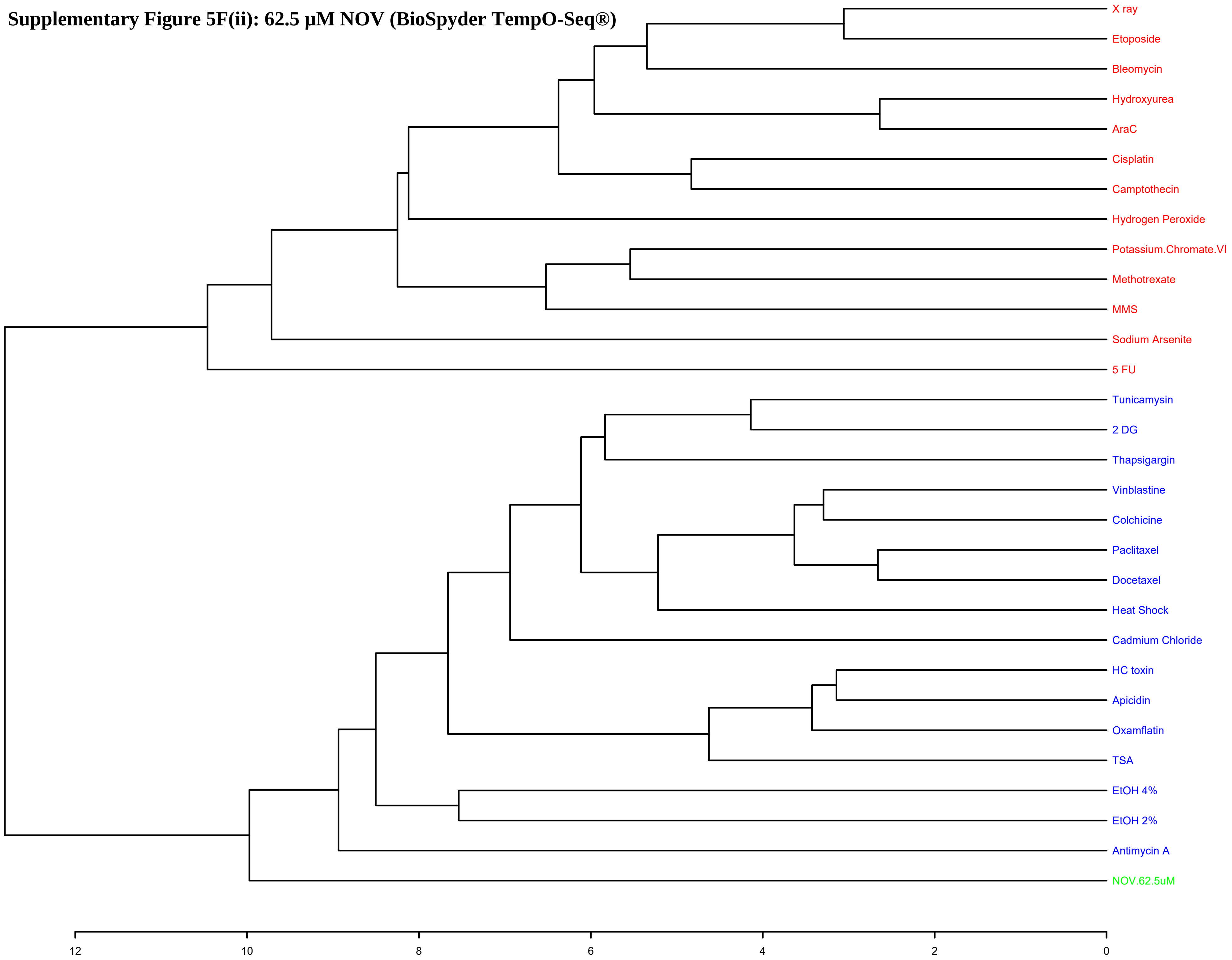

Supplementary Figure 5G(i): 125 µM NOV (NanoString nCounter®)

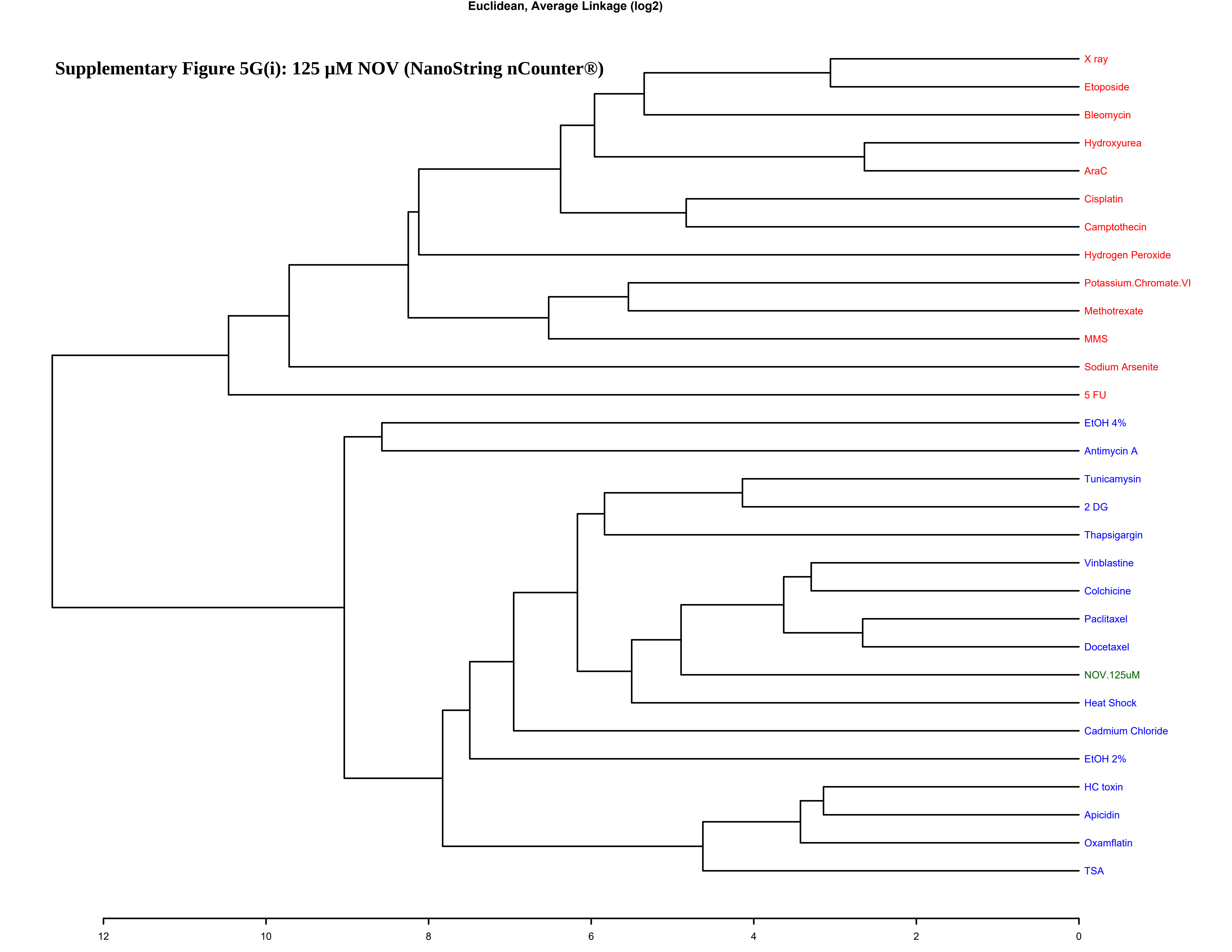

Euclidean, Average Linkage (log2)

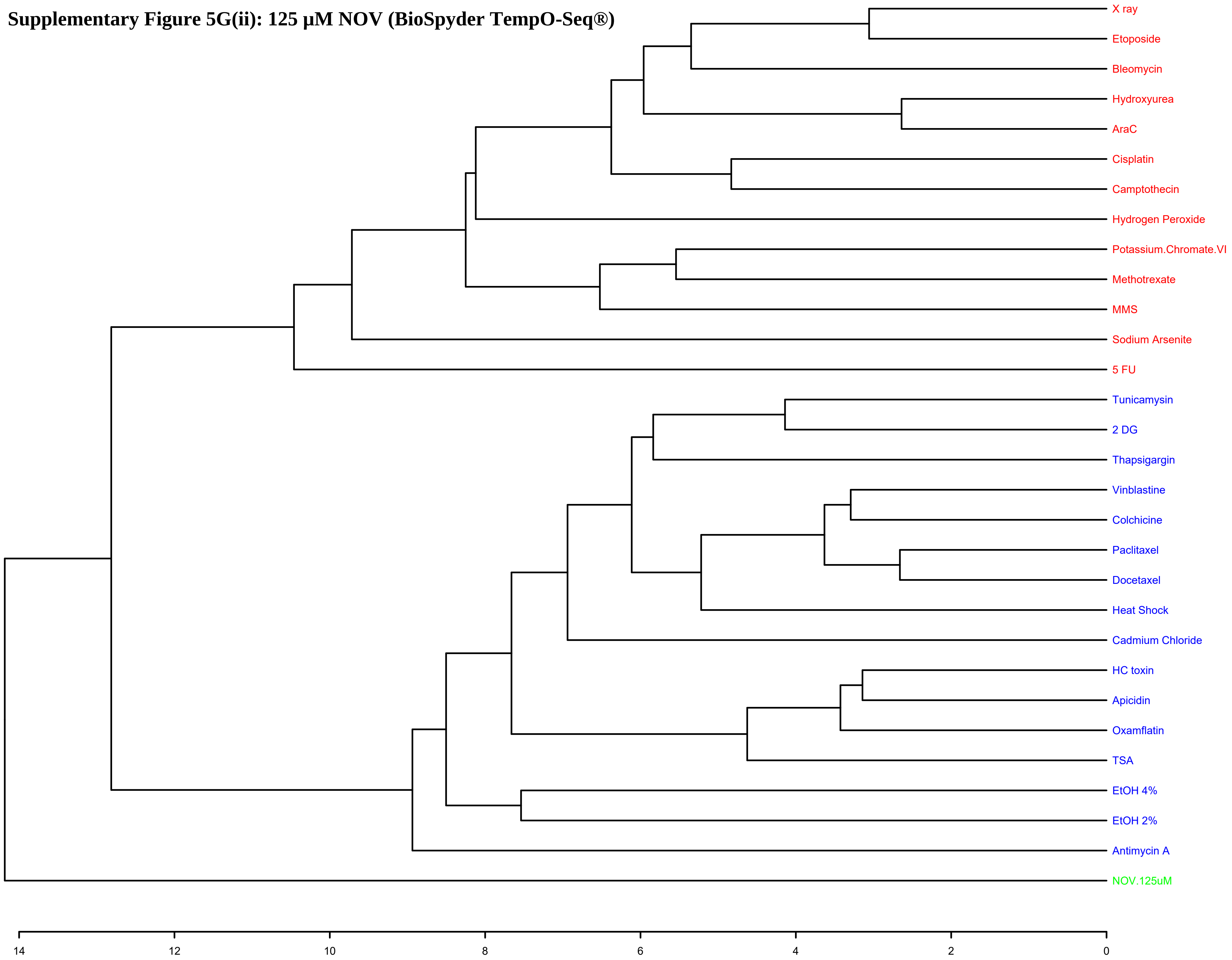

Euclidean, Average Linkage (log2)

Supplementary Figure 5H(i): 250 µM NOV (NanoString nCounter®)

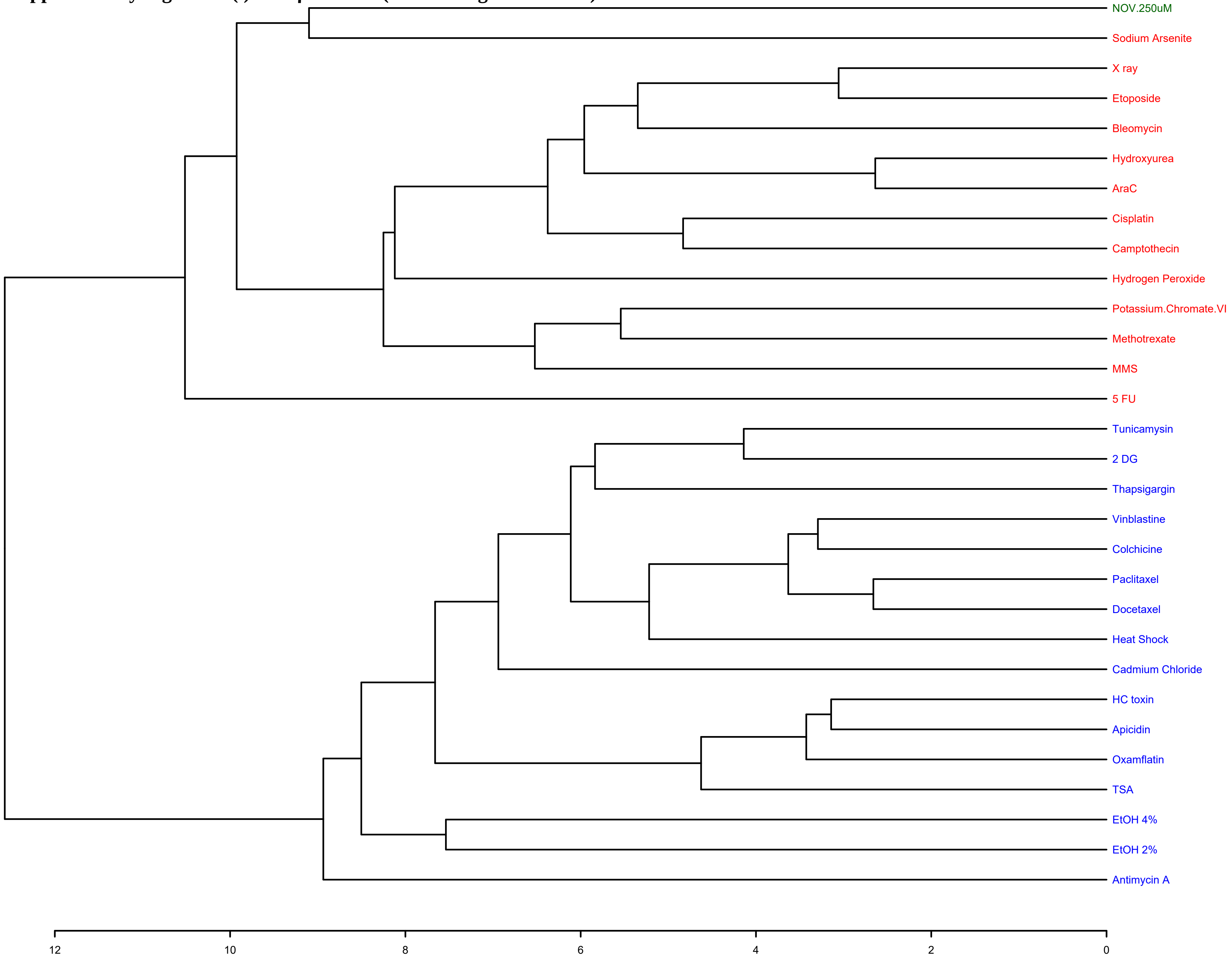

Euclidean, Average Linkage (log2)

Supplementary Figure 5H(ii): 250 µM NOV (BioSpyder TempO-Seq®)

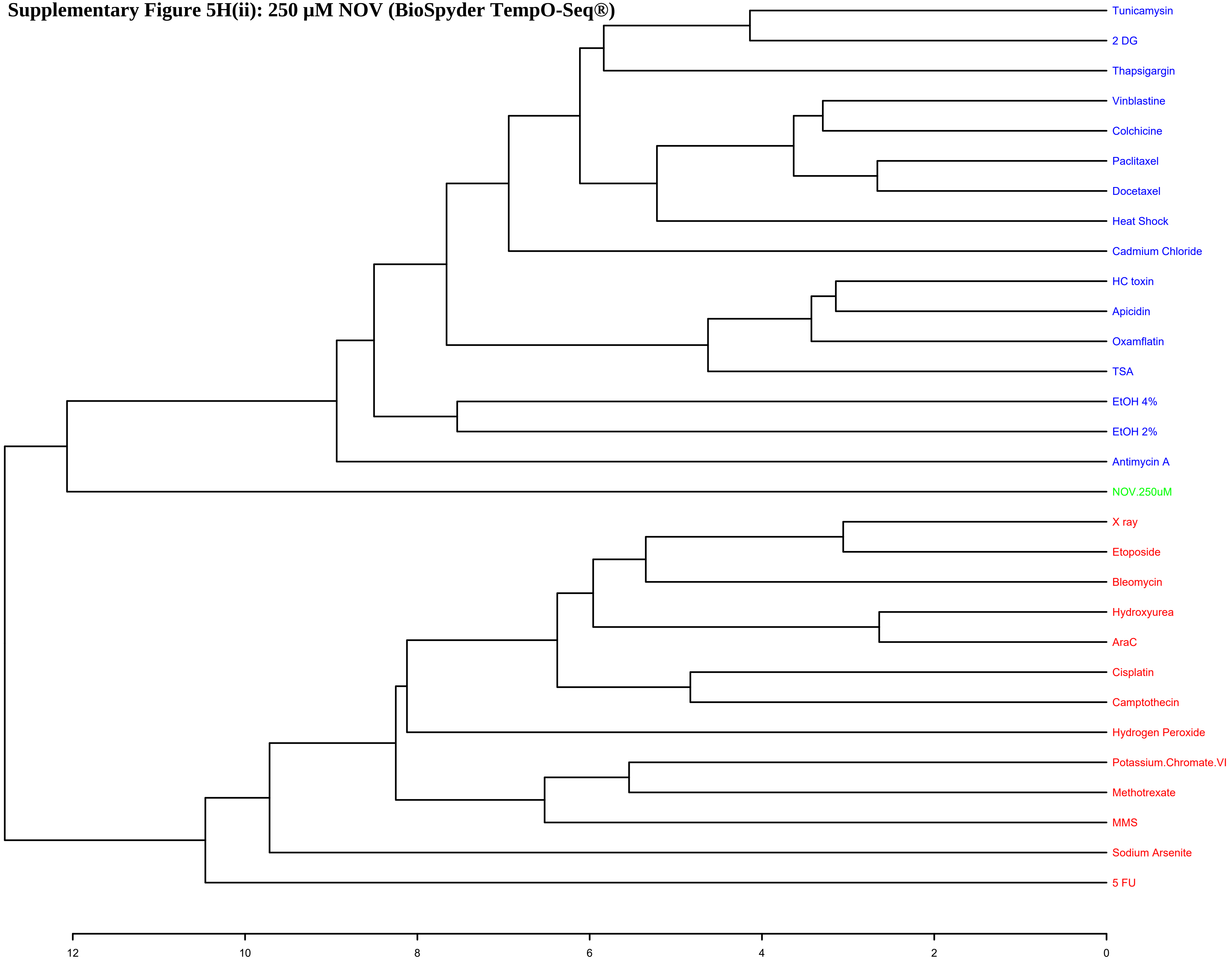

Euclidean, Average Linkage (log2)

Supplementary Figure 5I(i): 500 μM NOV (NanoString nCounter®)

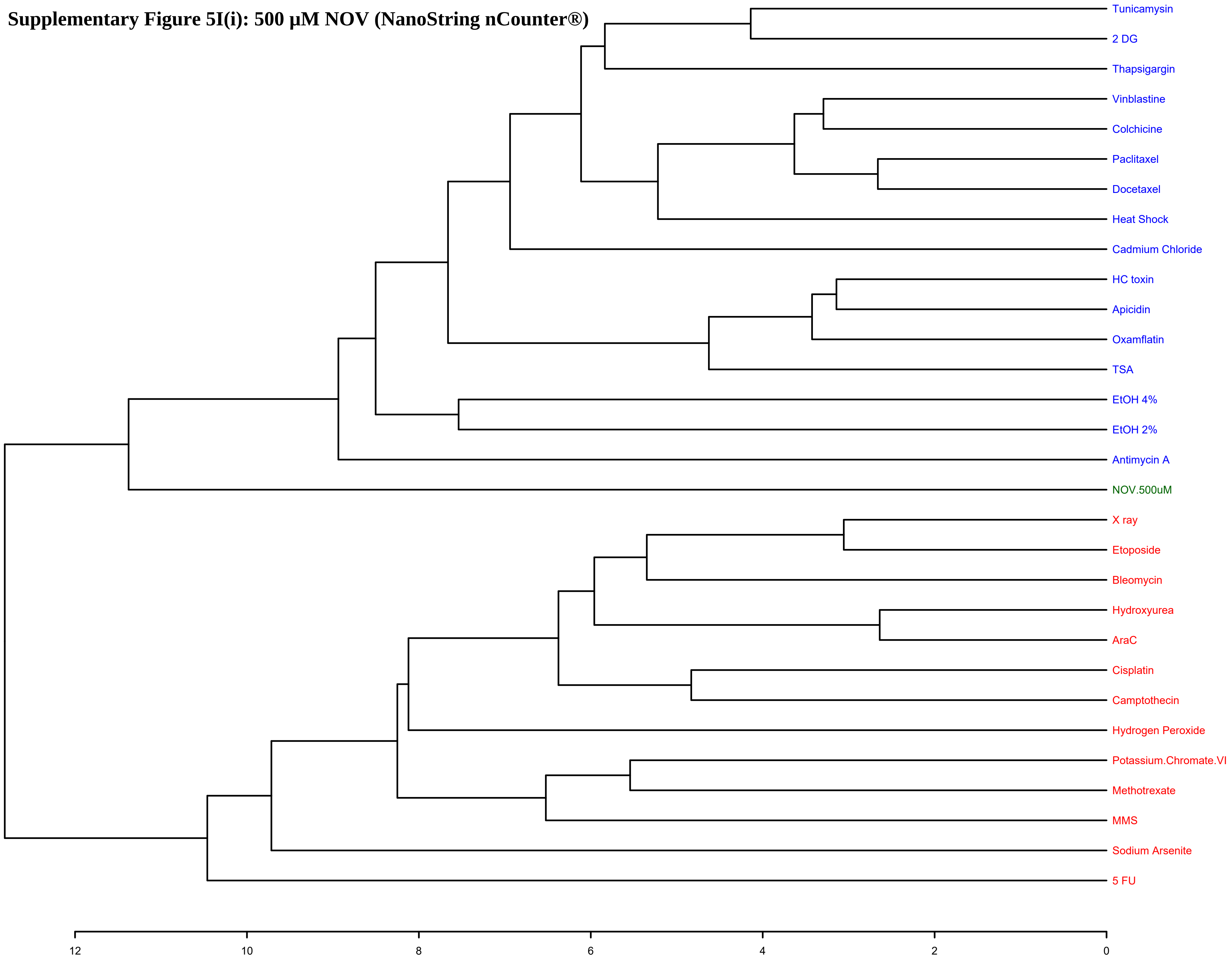

Euclidean, Average Linkage (log2)

Supplementary Figure 5I(ii): 500 µM NOV (BioSpyder TempO-Seq®)

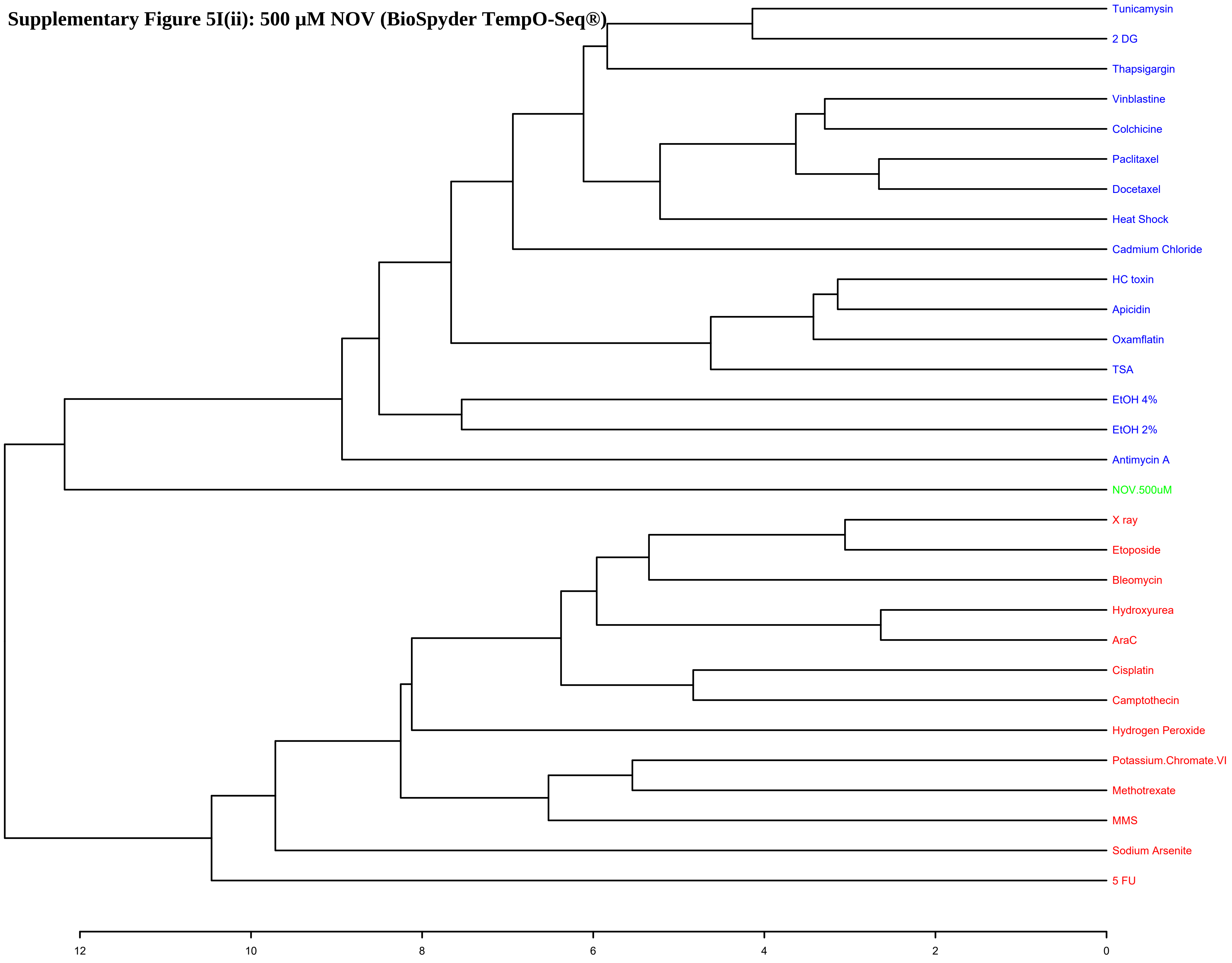

Supplementary Figures 2-5: Hierarchical clustering (HC) depicting TGx-DDI classification results for three case study chemicals shown in supplementary figures: (2A-H) nitrofurantoin (NIT), (3A-H) metronidazole (MTZ), and (4A-I) novobicin (NOV). The negative control (2 mM caffeine (CA)) and positive controls, including 10 µM bleomycin (BL) and 4 Gy ionizing radiation (IR) are shown in supplementary figure 5A-C. HC analysis using the TGx-DDI biomarker for TK6 cells exposed to the training set of chemicals (red font = DDI training set; blue font = non-DDI training set) and to three anti-infective drugs at increasing concentrations (2µM - 250 µM for NIT and MTZ and 2 µM - 500 µM for NOV) 4 hr after the last exposure (green font = replicates of test agent). Hierarchical clustering of the chemicals based on TGx-DDI classification analysis are shown in panel (i) for NanoString nCounter® data (dark green font) and panel (ii) BioSpyder TempO-Seq® data (neon green font). The main branch on the dendrogram separates the DDI and non-DDI agents and was used for classification of the test agent.
